# Oxidative [4+2] annulation of styrenes with alkynes under external-oxidant-free conditions

**DOI:** 10.1038/s41467-018-03534-z

**Published:** 2018-03-26

**Authors:** Guoting Zhang, Yulin Lin, Xu Luo, Xia Hu, Cong Chen, Aiwen Lei

**Affiliations:** 1 College of Chemistry and Molecular Sciences, the Institute for Advanced Studies (IAS), Wuhan University, Wuhan, Hubei 430072 China; 2 State Key Laboratory and Institute of Elemento-Organic Chemistry, Nankai University, Tianjin, 300071 China

## Abstract

The sequenced Diels–Alder/oxidation reaction represents a powerful route for the construction of aromatic compounds in organic synthesis. The oxidative Diels–Alder reaction with H_2_ evolution would be a more ideal approach that can avoid the additional oxidation procedure and stoichiometric oxidant. Herein, an oxidative [4 + 2] annulation reaction of styrene derivatives with electron-rich dienophiles accompanying the H_2_ generation has been developed by using the synergistic merger of photoredox and cobaloxime catalyst. With respect to atom and step-economy ideals, this dual catalytic system enables the formation of high-value molecules from feedstock chemicals in a single step under room temperature.

## Abstract (web summary)

A sequence of a Diels–Alder reaction and oxidation is a powerful route to valuable aromatic compounds. Here, the authors report a more atom-economical oxidant-free strategy involving a Diels–Alder with H_2_ evolution under noble-metal-free photoredox conditions.

## Introduction

**Fig. 1:**
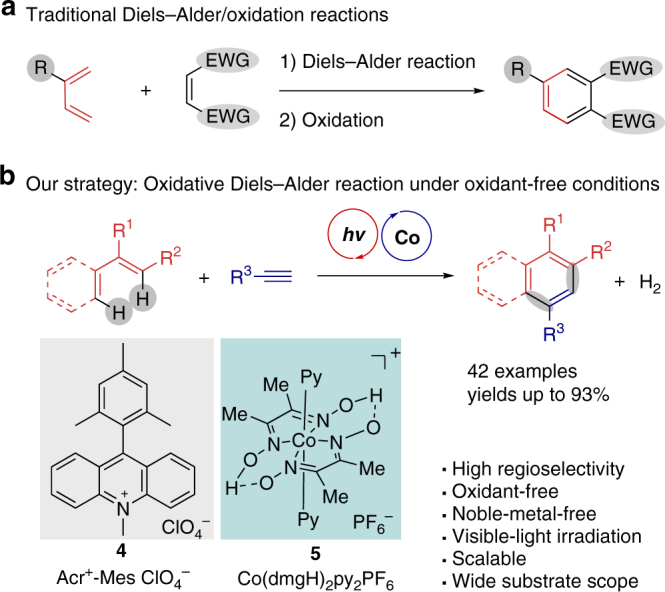
The formation of six-membered aromatic rings through Diels–Alder reactions. **a** Traditional D–A/oxidation reaction strategy. **b** Oxidative [4 + 2] annulation with H_2_ evolution

Six-membered aromatic rings are valuable structural motifs that widely existed in natural products, pharmaceuticals, and functional materials. The regioselective synthesis of multi-substituted arenes has long been attracted significant attention in industrial and academic society. Diels–Alder (D–A) reaction represents one of the most powerful tools for the C–C bond-constructing reactions in organic synthesis^1–5^. Followed by sequenced oxidative aromatization, D–A reaction has be utilized into the preparation of multi-substituted aromatic compounds by building up a new six-membered carbocycle (Fig 1a) ^6–9^. By this approach, the regioselective introduction of functional groups on arenes can be guaranteed, since the partial substituents of aromatics are preinstalled into the starting materials.

In the classic D–A reaction, butadiene reacts with ethylene to form cyclohexene, and, overall, one unsaturated bond functionality is lost. The six-membered aromatic rings can be formed by the D–A reaction between the pre-dehydrogenative high-valence reactants. For instance, alkyne and allenyne combine to produce benzene in tetradehydro-Diels–Alder (TDDA) reaction^10^. Beyond that, Hoye et al. have also developed new types of D–A reactions, hexadehydro-Diels–Alder (HDDA) and pentadehydro-Diels–Alder (PDDA) reactions, which are powerful tools for the synthesis of arenes^11,12^. Undoubtedly, a dehydrogenative D–A reaction that can retain unsaturated bond during the formation of a six-membered ring would be a more ideal approach from the view of step- and atom-economy^13^, since the pre-oxidation and stoichiometric oxidants can be avoided. A microwave-assisted intramolecular dehydrogenative D–A reaction of styrene-yne to form naphthalene derivatives without oxidant was developed by Brummond^14^, which was generically called as the dehydro-Diels–Alder (DDA) reaction^8,15–17^. The dehydrogenative [4 + 2] annulation under mild conditions still remains challenging, because high reaction temperature was generally required to overcome the high activation energy in the thermal D–A reaction.

Thus, a suitable catalytic system that can facilitate the dehydrogenative [4 + 2] annulation reaction under ambient temperature would be highly desirable. We hypothesized that this goal is attainable through an oxidation-induced radical-cation D–A reaction pathway. Recently, the alkene radical cation species has been increasingly utilized as key intermediate in the organic synthesis^18–20^. The radical cation D–A reaction has already emerged as a useful synthetic method, which can be carried out under milder conditions with high regioselectivity and reactivity than the analogous thermal [4 + 2] cycloaddition involving neutral or electron-rich dienophiles^21–24^. The photoredox catalysis provides a convenient tool to access such highly reactive odd-electron species under mild conditions^25–36^. In particular, the photo-induced radical cation oxidative D–A reaction of 1,1-diphenylene with alkenes by using stoichiometric 1,4-dicyanobenzene as the sensitizer and oxidant was reported respectively by Arnold and Farid^37,38^. Recently, Huang and Nicewicz have developed a visible-light-mediated [4 + 2] cycloaddition of styrenes by using acridinium photosensitizer and hydrogen atom transfer catalyst^24^. To our best knowledge, the dehydrogenative photo-induced oxidative [4 + 2] annulation process that requires no external oxidant remains elusive. The photoredox/cobaloxime dual catalytic system has emerged as a powerful tool for the oxidative cross-coupling reactions with H_2_ evolution^39–45^.

In this work, we developed a highly regioselective oxidative D–A reaction with H_2_ evolution by using a photo/cobaloxime dual catalytic system (Fig. 1b). A variety of six-membered aromatic rings can be successfully afforded from [4 + 2] annulation of styrene derivatives with electron-rich dienophiles under room temperature. The alkenes radical cation species was proposed as the key intermediate for the annulation reaction. It is appealing to further apply this method for the synthesis of the important functional compounds because of its mildness and high atom economy features of this method.

## Results

### Optimization of the reaction conditions

Our initial investigation began with the [4 + 2] annulation of 1,1-diphenylethlene with 4-ethynylanisole. This electronically dismatched D–A cycloaddition would not proceed upon thermal activation (200 °C, 24 h, see Supplementary Fig. 3). Encouragingly, the reaction was conducted by employing the Fukuzumi’s acridinium^46^ as the photosensitizer and Co(dmgH)_2_pyCl (**5S-1**) as the co-catalyst (Supplementary Table 1, entry 1), the desired naphthalene product **3aa** can be isolated in 66% yield after the irradiation of blue LEDs light for 24 h. The single regioselectivity and only a little of dimerization side-product of alkene can be observed. The H_2_ can be determined by gas chromatography with thermal conductivity detector (GC-TCD) and some amount of hydrogenation of olefin can be obtained. The exploration of various cobaloxime catalysts revealed that a modified cobaloxime Co(dgmH)_2_py_2_PF_6_ (**5**) would improve the reactivity of this transformation (75%). However, chemoselectivity became particularly problematic when the conditions were used for other easily oxidizable olefin, such as prop-1-ene-1,1-diyldibenzene. It was found that both [2 + 2] cycloaddition and [4 + 2] dehydrogenative annulation can occur. The [4 + 2] pathway is favored by decreasing concentration of reaction (Supplementary Table 2). Control experiments indicated no desired reaction would be observed when each component was individually omitted (Supplementary Table 3).

### Substrate scope of the reaction

**Fig. 2:**
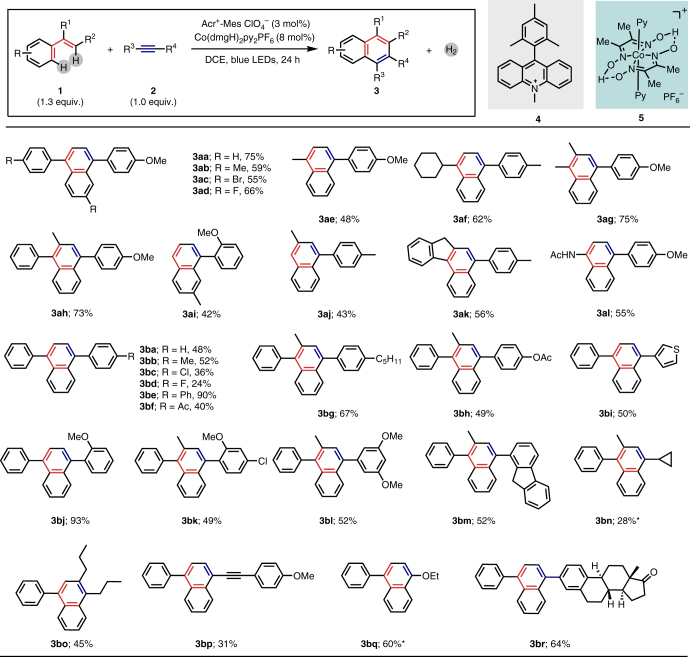
Substrate scope of oxidative [4 + 2] annulation of styrenes with alkynes. Reaction conditions: alkene **1** (0.26 mmol), alkyne **2** (0.2 mmol), Acr^+^-Mes ClO_4_^−^ (3 mol%), Co(dmgH)_2_py_2_PF_6_ (8 mol%) in DCE (14 mL), 3W blue LEDs, 24 h; isolated yields are shown. ^*^0.2 mmol alkene and 0.6 mmol alkyne were used

With this dual photoredox/cobalt catalytic system in hand, we then examined the scope and limitation of this protocol. This dual catalytic system allows this oxidative [4 + 2] annulation of a wide range of styrenes with alkynes (Fig. 2). The 1,1-diphenylethene derivatives bearing electron-donating or electron-withdrawing substituents underwent the reaction smoothly with 4-methoxyphenylacetylene to produce the desired naphthalenes (**3aa**–**3ad**). When one of the aryl groups was replaced by the alkyl group (**3ae**, **3af**), the transformation process could also proceed with moderate to good yields. The procedure was also applicable for trisubstituted aryl alkenes (**3ag**, **3ah**). A mono-substituted aryl alkene, 4-methylstyrene participated in the reaction to provide the desired product in moderate yield (**3ai**). Internal alkenes, such as β-methyl styrene can produce the corresponding naphthalene **3aj** in moderate yield with 1-ethynyl-4-methylbenzene. When *N*-(1-phenylvinyl)acetamide was subjected to the same reaction conditions, transformation occurred smoothly to yield the 1-naphthylamine derivative (**3al**) in 55% yield.

Next, the scope of the reaction with respect to alkynes was investigated. Various substituted alkynes changing with the electronic and steric properties of substituents were applicable for this protocol (**3ba**–**3br**). Electrically neutral phenyl acetylene provided the 1,4-diphenylnaphthalene in 48% yield (**3ba**). Generally, an electron-rich dienophile promotes this oxidative radical cation D–A reaction due to the higher nucleophilicity. The presence of electron-donating groups (–Me, –OMe, 4-C_5_H_11_, 4-OAc) would increase the reactivity (**3bb**, **3aa**, **3bg**, **3bh**), while electron-withdrawing groups, such as halides (**3bc**, **3bd**) and acetyl (**3bf**), provided relatively lower yields. Both *ortho-* and *meta-*substituted aromatic alkynes could provide the desired products (**3bj**, 93% and **3bl**, 52%). The 4-chloro-1-ethynyl-2-methoxybenzene gave the desired product **3bk** in moderate yield (49%). This method could be extended to the heteroaromatic alkyne (**3bi**). Furthermore, the aliphatic alkyne led to the corresponding products in modest yield, and the cyclopropyl group can be tolerated (**3bn**). The reactivity of this reaction would be almost shut down when 1,2-diphenylethyne was used as a dienophile might due to electro-withdrawing property of phenyl group. However, the transformation is suitable for the internal alkyne oct-4-yne to form the desired product (**3bo**). When a conjugated diyne, 1-(buta-1,3-diyn-1-yl)-4-methoxybenzene, was used as a substrate, the [4 + 2] annulation occurred in the terminal alkyne group (**3bp**). Alkynyl ether can also be compatible in this transformation to form the α-naphthylether compound (**3bq**). An estrone derivative (**3br**) was successfully prepared with moderate yield, which highlights the good functional group tolerance and potential applications of this method. Although the conversion of alkyne is high in some examples, the yields are low and other side-product can not be observed. We speculated that a part of the alkynes might polymerize under the light-illumination condition.

**Fig. 3:**
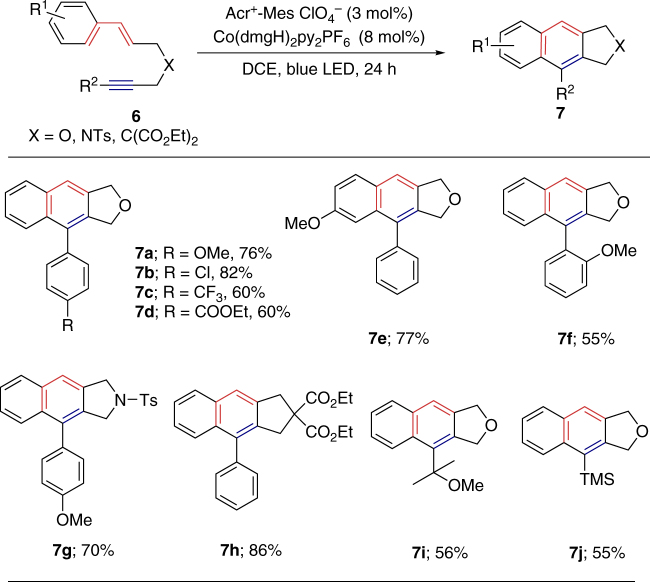
Intramolecular oxidative [4 + 2] annulation with respect to alkynes. Reaction conditions: styrene-yne **6** (0.2 mmol), Acr^+^-Mes ClO_4_^−^ (3 mol%), Co(dmgH)_2_py_2_PF_6_ (8 mol%) in DCE (14 mL), 3W blue LEDs, 24 h; isolated yields are shown

Notably, this procedure was also applicable for the intramolecular dehydrogenative [4 + 2] cycloaddition reaction of styrene-ynes (Fig. 3). Compared with the Brommund’s report^14^, this dual catalytic system allows the achievement of this transformation without the need of severe reaction temperature. The substrate scope of this photocatalyzed protocol is different from the previously reported thermal reaction. The electron-rich alkynes (**7a** and **7b**) would promote the reaction, while electron-deficient alkynes (**7c** and **7d**) decreased the reactivity. The electro-rich substituent on the para position of styrene can be tolerated to give the product in good yield (**7e**, 77%). 1-(3-(Cinnamyloxy)prop-1-yn-1-yl)-2-methoxybenzene gave the desired product with decreased reactivity due to the steric effect (**7f**, 55%). The toluenesulfonamide and diethyl malonate substrates can also afford the desired products (**7g** and **7h**). Furthermore, the substitution of the alkyne with alkyl groups could be carried out with ease in good yields (**7i** and **7j**).

**Fig. 4:**
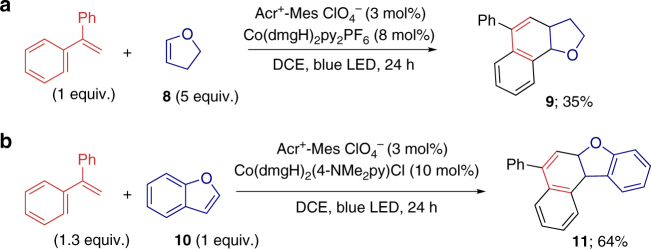
Oxidative D–A reactions of styrene with alkene or arene. Reaction conditions of **a** 1,1-diphenylethene (0.2 mmol), 2,3-dihydrofuran **8** (1 mmol), Acr^+^-Mes ClO_4_^−^ (3 mol%), Co(dmgH)_2_py_2_PF_6_ (8 mol%) in DCE (5 mL), 3W blue LEDs, 24 h. Reaction conditions of **b**: 1,1-diphenylethene (0.26 mmol), benzofuran **10** (0.2 mmol), Acr^+^-Mes ClO_4_^−^ (3 mol%), Co(dmgH)_2_(4-NMe_2_py)Cl (10 mol%) in DCE (4 mL), 3W blue LEDs, 24 h

Besides alkynes, other electron-rich dienophiles were also suitable for this oxidative D–A reaction (Fig. 4). An electron-rich olefin, 2,3-dihydrofuran (**8**), coupled with 1,1-diphenylethene to afford the dihydronaphthalene (**9**, 35% yield). Benzofuran (**10**), an electron-rich heterocyclic aromatic compound, can also act as dienophile to participate this oxidative [4 + 2] annulation protocol (**11**, 64%).

### Gram synthesis using sunlight

**Fig. 5:**
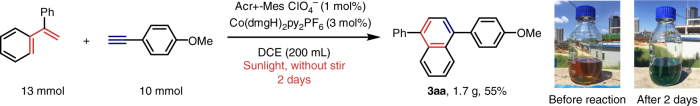
Gram synthesis using sunlight source. Reaction conditions: the mixture of 1,1-diphenylethene **1a** (13 mmol), 1-ethynyl-4-methoxybenzene **2a** (10 mmol), Acr^+^-Mes ClO_4_^−^ (1 mol%), Co(dmgH)_2_py_2_PF_6_ (3 mol%) in DCE (200 mL) was placed in a sunny place for 2 days; isolated yield was showed

To demonstrate the scalability of this protocol, a gram-scale synthesis of **3aa** (10 mmol scale) was performed by employing only 1 mol% of photosensitizer and 3 mol% of cobaloxime catalyst. Furthermore, this reaction even could be performed by using sunlight source (Fig. 5). When the reaction was placed in a sunny place for 2 days without stir, 55% yield of product can be obtained.

### Mechanistic studies

**Fig. 6:**
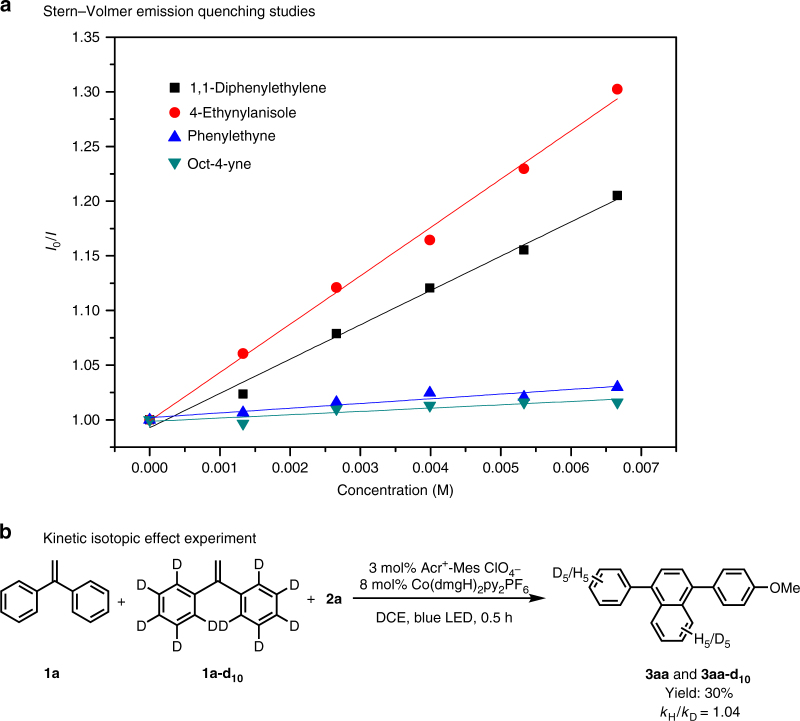
Mechanistic studies. **a** Stern−Volmer emission quenching studies; **b** kinetic isotopic effect experiment

To obtain further insight on the photocatalytic process, the Stern–Volmer studies of photocatalyst were conducted and the results were shown in Fig. 6a. It was found that fluorescence intensity of the excited state of **4** can be linearly quenched by 1,1-diphenylethene (**1a**) and 4-ethynylaniosole (**2a**). In contrast, the reactive electron-neutral phenylethyne or alkyl alkyne fail to quench the emission of the excited state of the photosensitizer. Therefore, we proposed that the alkynes preferred to serve as the dienophiles, though the initial oxidation of alkyne cannot be completely ruled out when the strong electron-rich aryl alkynes were used. To gain insight of this transformation, the competition experiment between the substrates with electron-rich and electron-deficient substituent was conducted (Supplementary Fig. 9). The electron-rich substituents would promote the reactivity, which indicated that a positive charge intermediate might be involved in the transformation. The reaction of the same amount of substrate **1a** and deuterated substrate **1a-d**_**10**_ under the standard conditions for 0.5 h provided a mixture of the products deuterium-**3aa** and **3aa** in 31% combined yield, in which the ratio of **3aa**: **3aa-d**_**10**_ was 1.04 (Fig. 6b). The kinetic isotopic effect (KIE) value revealed that the cleavage of aromatic C–H bond might not be the rate-determining step of the transformation.

### Proposed mechanism

**Fig. 7:**
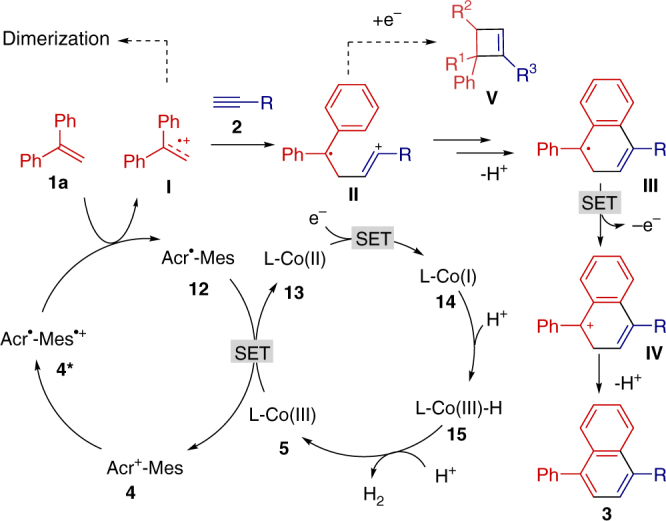
Proposed mechanism. The plausible mechanism involves oxidation of alkene to generate alkene radical cation, nucleophilic attack of dienophiles and visible-light photoredox/cobaloxime catalyzed formation of six-membered aromatic rings and H_2_

The proposed mechanism was outlined in Fig. 7. The dimerization of alkene can be detected by GC–MS, which indicated that alkene radical cation might act as an intermediate in the reaction. We believed that the styrene radical cation can be formed upon the electron transfer from the styrene to an excited-state photoredox catalyst 4*. Then, the nucleophilic attack of dienophile to radical cation **I** would furnish a distonic radical cation **II**, which further cyclized and deprotonated to form a C-radical intermediate **III**. The oxidation and proton elimination of **III** produced the desired aromatic compounds. In the cobalt catalytic cycle, the Co(III) species **5** can be reduced to provide a Co(I) species **14**, which would generate a Co(III)-hydride species **15** by reacting with a released proton. The interaction between Co(III)-hydride speices **15** and another proton could release H_2_ and complete the catalytic cycle^47–50^. It was found that the [4 + 2] pathway is favored by decreasing the reaction concentrations. We proposed that the radical cation intermediate has time to intramolecularly rearrange to the more stable [4 + 2] product at low reaction concentration, while the radical cation **II** can be facilely reduced by starting materials to give cyclobutene at high reaction concentration.

Overall, this dehydrogenative [4 + 2] annulation reactions is a powerful tool for the synthesis of polysubstituted aromatics from commercially available materials. Through an in situ-generated alkene radical cation intermediate, the reaction can be carried out under room temperature with good regioselectivity. The mildness and high atom economy of this approach makes it appealing for further application to synthesis of important natural products, pharmaceuticals, and functional materials.

## Methods

### General procedure for catalytic oxidative [4 + 2] cycloaddition of styrene with alkynes

A schlenk tube equipped with a stir bar was loaded with 2.4 mg (3 mol%, 0.006 mmol) of Acr^+^-Mes ClO_4_^−^, 9.5 mg (8 mol%, 0.016 mmol) of Co(dmgH)_2_py_2_PF_6_, 0.26 mmol styrenes **1** and 0.2 mmol alkyne **2** in 14 mL degassed DCE under N_2_ atmosphere. The solution was then stirred at room temperature under the irradiation of blue LED lamp for 24 h. After the completion of reaction, the product was determined by thin layer chromatography (TLC) and H_2_ can be detected by GC-TCD. The solvent was removed under reduced pressure by an aspirator, then the pure product was obtained by flash column chromatography on silica gel (eluent: petroleum ether/dichloromethane = 10:1) to afford corresponding naphthalene products **3**. See Supplementary Methods for further experimental details.

### Data availability

The data supporting the findings of this work are available within the article and its Supplementary Information files. All other relevant data supporting the findings of this study are available from the corresponding author on request.

## Electronic supplementary material

Supplementary Information(PDF 3510 kb)
