# Supplementary material for: Oxidative [4+2] annulation of styrenes with alkynes under external-oxidant-free conditions
Source: Nat Commun. 2018 Mar 26;9:1225. doi: 10.1038/s41467-018-03534-z (PMC5964314; doi:10.1038/s41467-018-03534-z)
Supplement: Supplementary file 1 — Supplementary Information(PDF 3510 kb) [file 41467_2018_3534_MOESM1_ESM.pdf]

Supplementary Information

Oxidative [4+2] Annulation of Styrenes with Alkynes under External-Oxidant-Free Conditions

Zhang et, al.

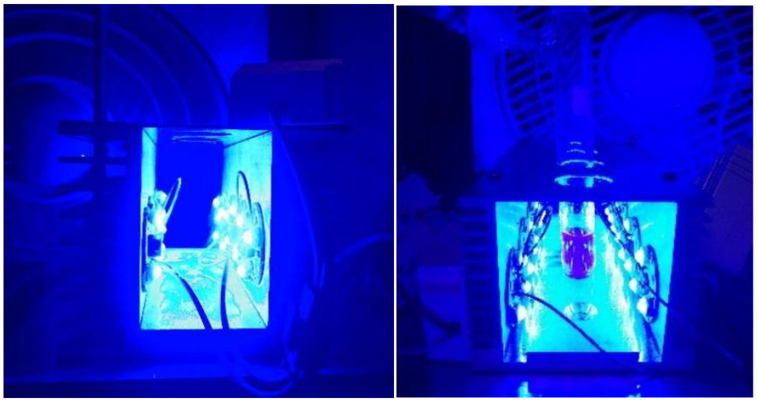

Supplementary Figure 1. The blue LEDs photochemical setup.

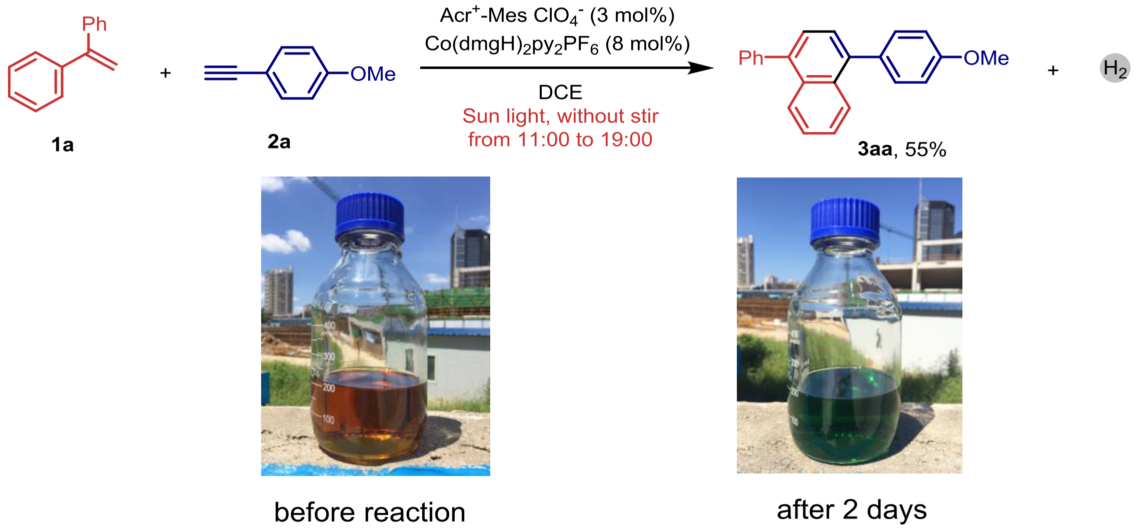

Supplementary Figure 2. Oxidative [4+2] annulation using sunlight as the light source. Conditions: In an 500 mL glass vial, Acr$^+$-Mes ClO_4^- (41 mg, 0.1 mmol), $Co(dmgH)_2py_2PF_6$ (178 mg, 0.3 mmol), 1,1-diphenylethylene (2.4 mL, 13 mmol) and 4-ethynylanisole (1.4 mL, 10 mmol) were combined. Then DCE (200 mL) was added. The solution was degased by purging the N_2 flow for 30 mins. The vial was placed in a sunny place for 2 days. The mean temperature of this area is about 33 degrees in summer. After the completion of reaction, the products was determined by TLC. The solvent was removed under reduced pressure by an aspirator, then the pure product was obtained by flash column chromatography on silica gel (eluent: petroleum ether/dichloromethane = 10:1) to afford **3aa** in 55% yield.

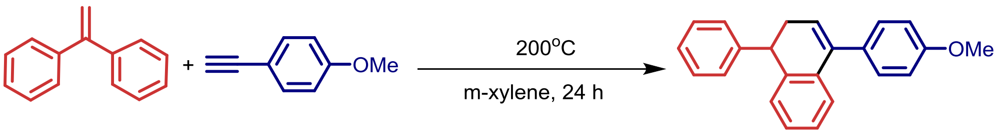

1a **2a** **< 5%**

Supplementary Figure 3. Thermal Diels-Alder reaction of styrene with electron-rich alkyne. Reaction conditions: the mixture of **1a** (0.26 mmol), 1-ethynyl-4-methoxybenzene **2a** (0.2 mmol) in m-xylene was heated under 200°C for 24 h; product was determined by GC.

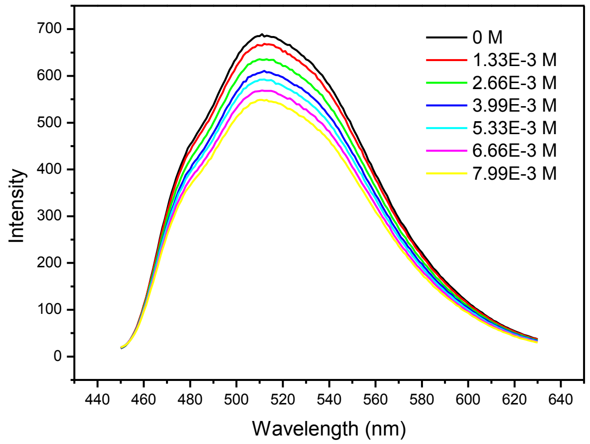

Supplementary Figure 4. Acr$^+$-Mes ClO_4^- emission quenching by 1,1-diphenylethene.

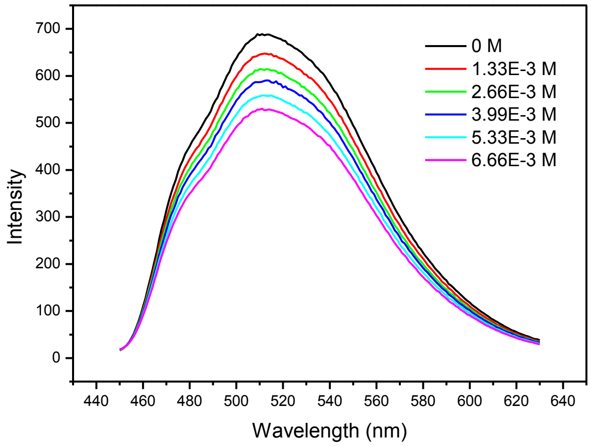

Supplementary Figure 5. Acr^{+}-Mes ClO_4^{-} emission quenching by 4-ethynylanisole.

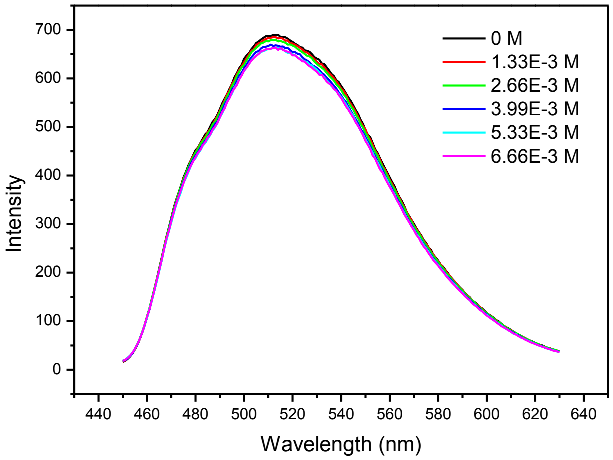

Supplementary Figure 6. Acr^+-Mes ClO_4^- emission quenching by phenylethyne.

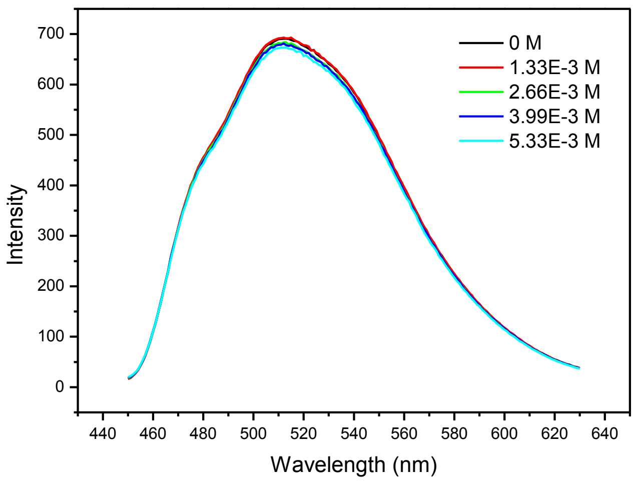

Supplementary Figure 7. Acr$^+$-Mes ClO_4^- emission quenching by oct-4-yne.

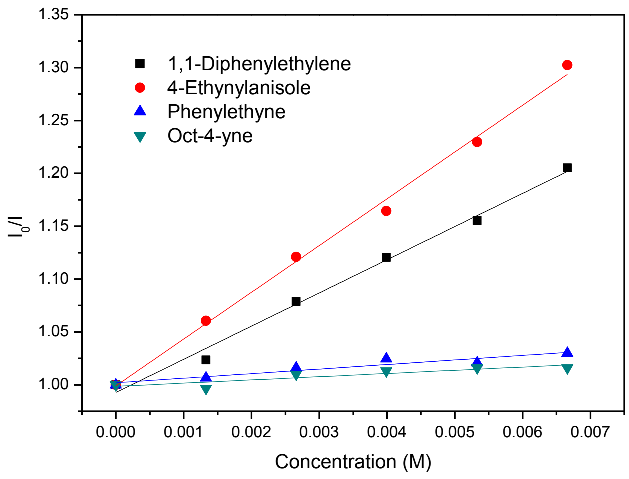

Supplementary Figure 8. Stern−Volmer emission quenching studies of Acr$^+$-Mes ClO_4^- by 1,1-diphenylethene, 4-ethynlanisole, phenylethyne and oct-4-yne.

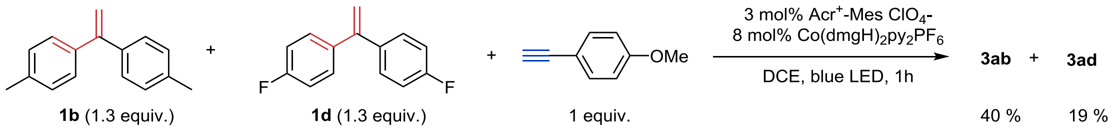

Supplementary Figure 9. The competition experiment. Reaction conditions: the mixture of **1b** (0.26 mmol), **1d** (0.26 mmol), 1-ethynyl-4-methoxybenzene **2a** (0.2 mmol), Acr^{+}-Mes ClO_4^{-} (3 mol%), $Co(dmgH)_2py_2PF_6$ (8 mol%) in DCE (200 mL), blue LEDs, 1h; yields were determined by GC.

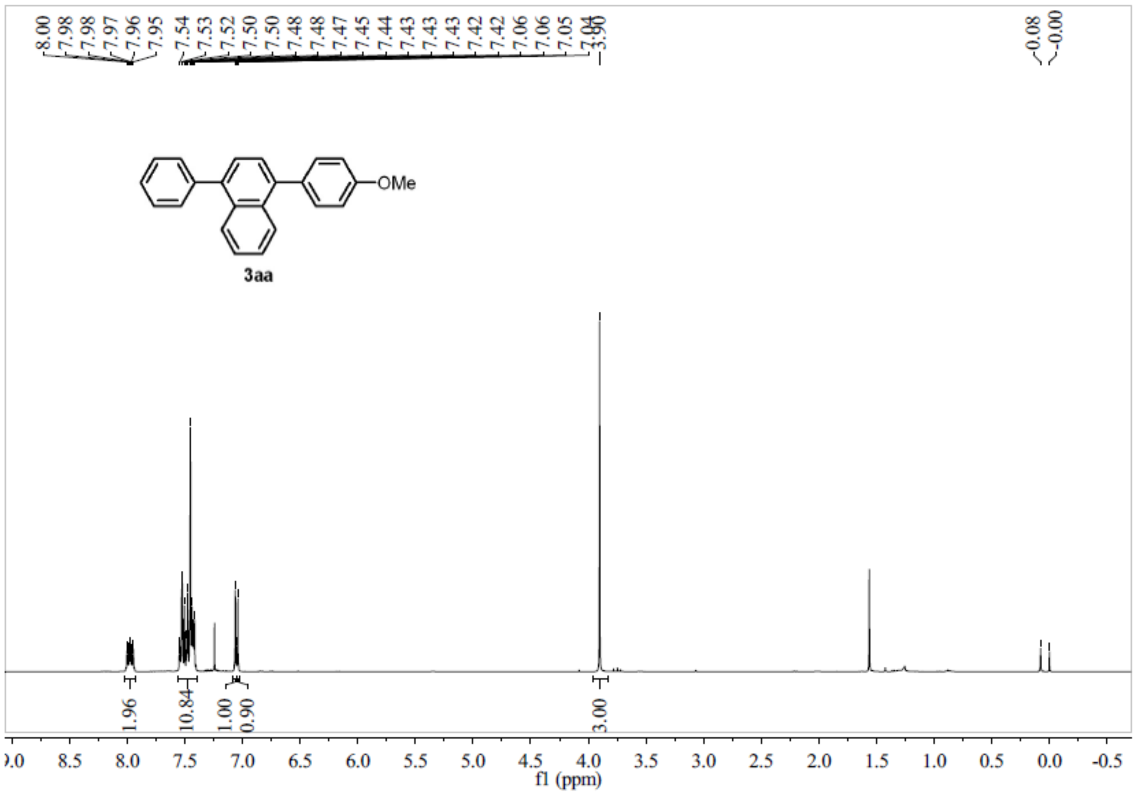

Supplementary Figure 10. ^{1}H NMR of **3aa**

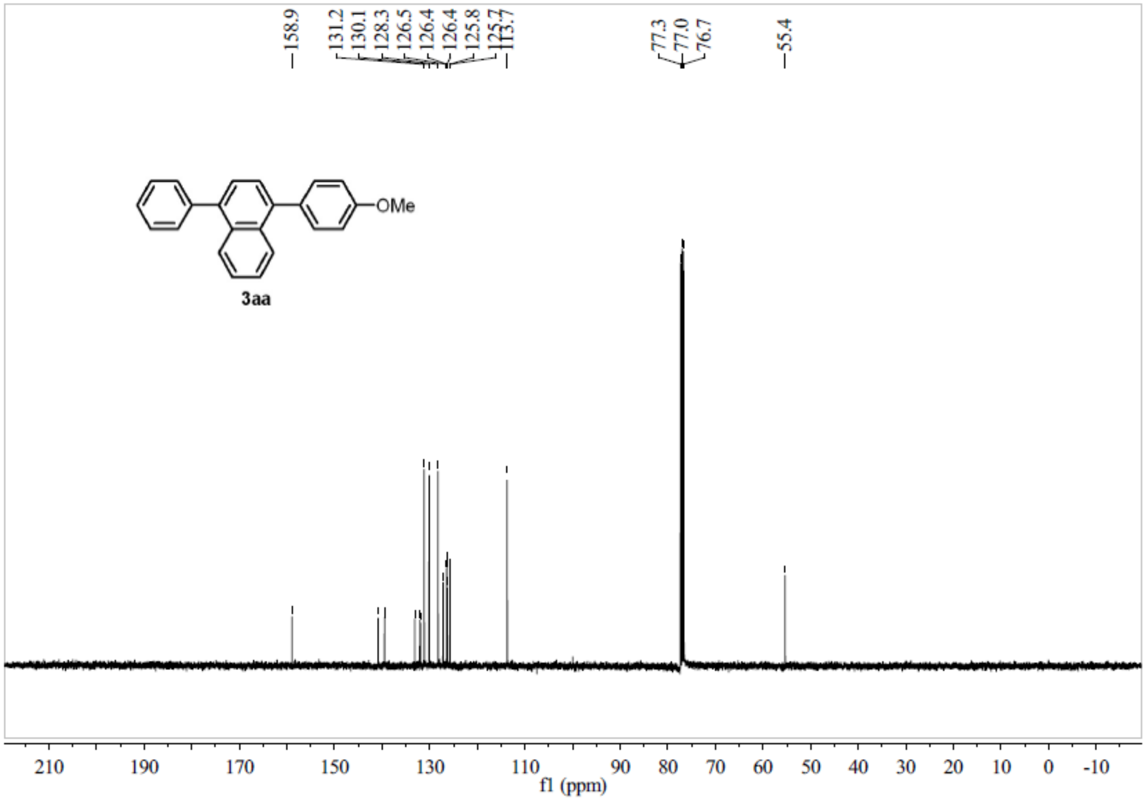

Supplementary Figure 11. ^{13}C NMR of **3aa**

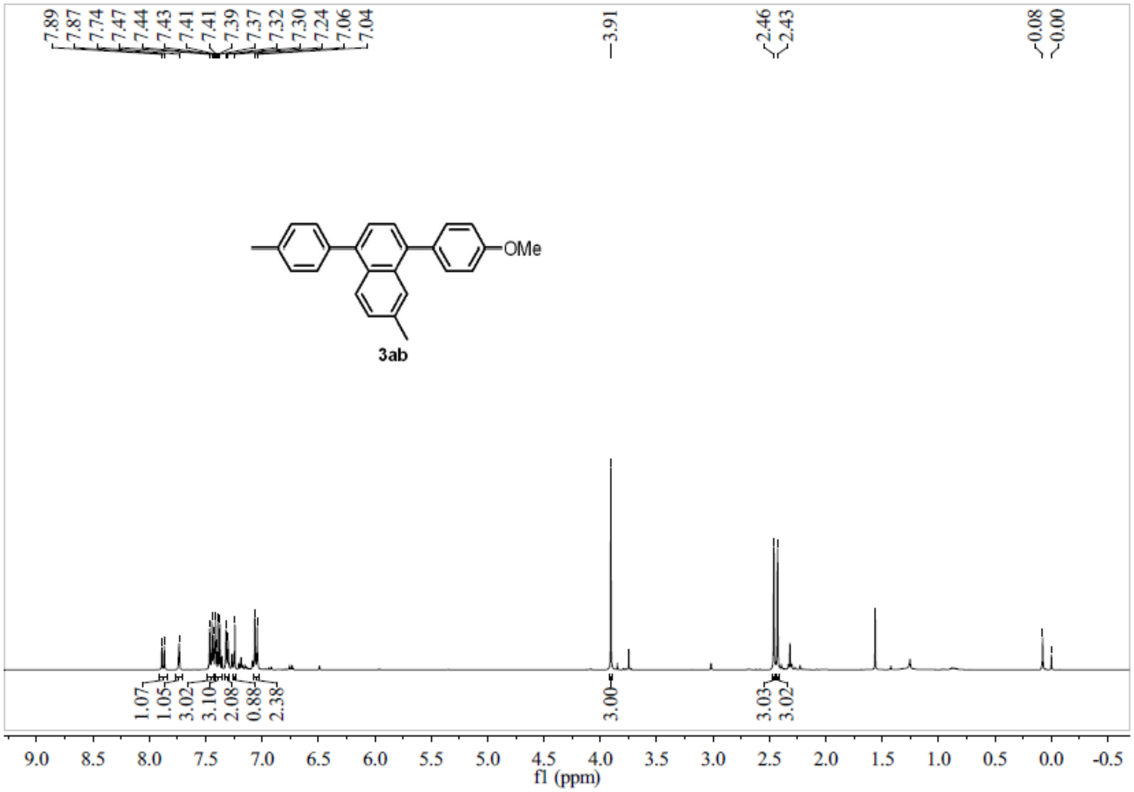

Supplementary Figure 12. ^{1}H NMR of **3ab**

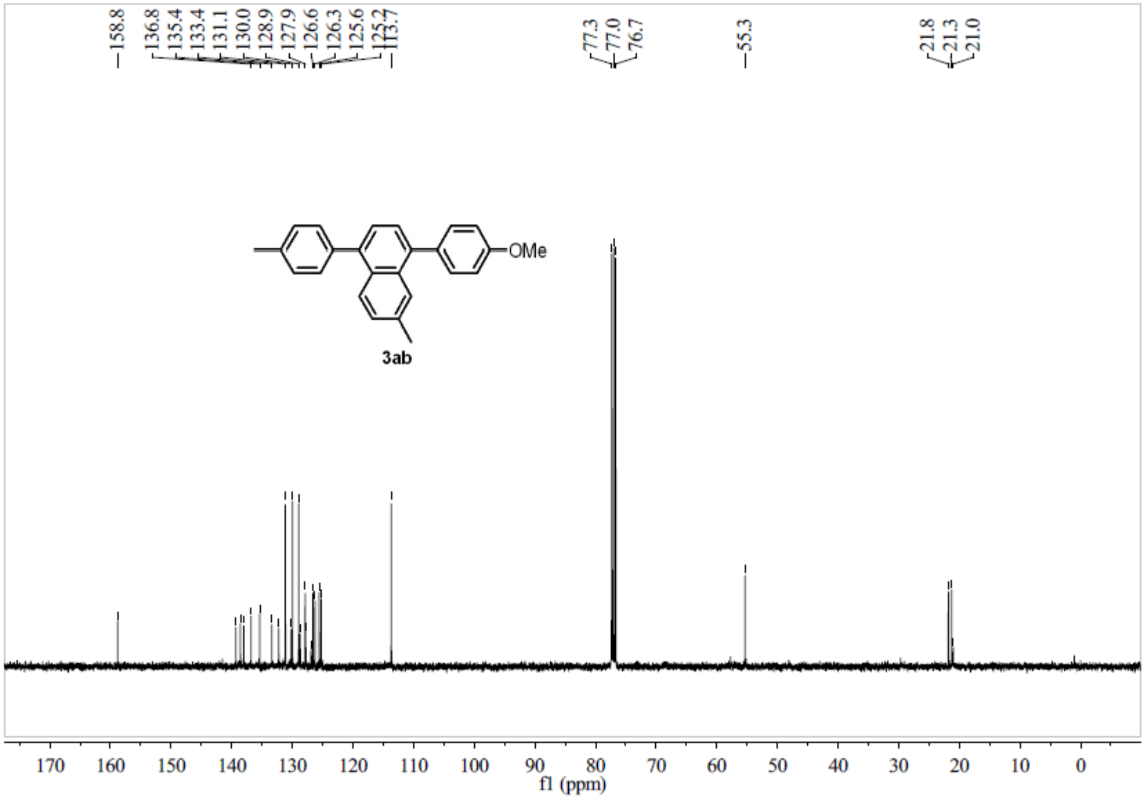

Supplementary Figure 13. ^{13}C NMR of **3ab**

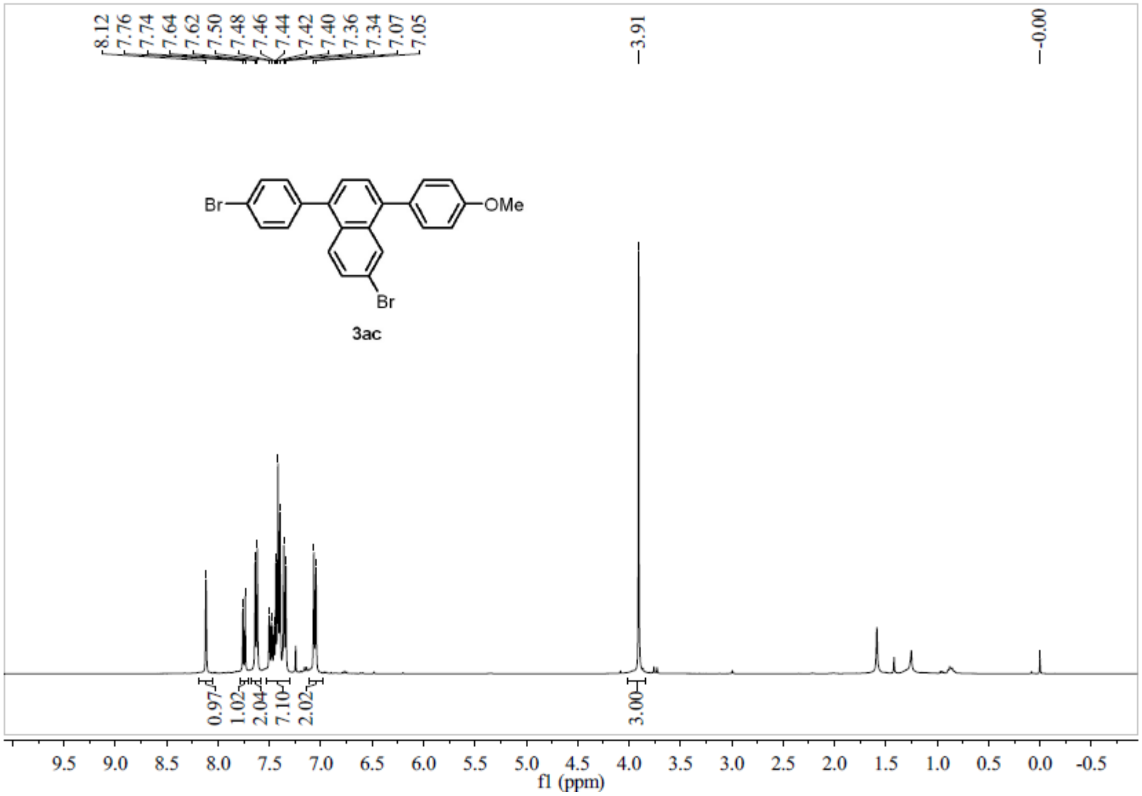

Supplementary Figure 14. ^{1}H NMR of **3ac**

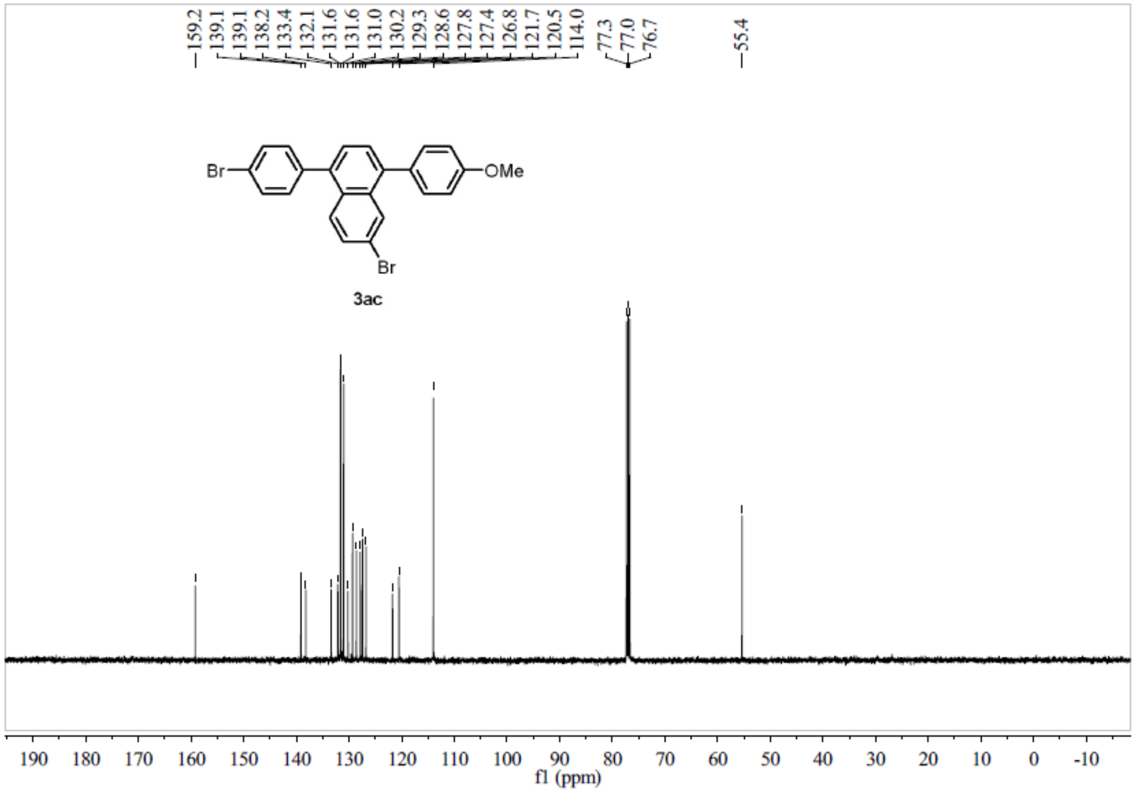

Supplementary Figure 15. ^{13}C NMR of **3ac**

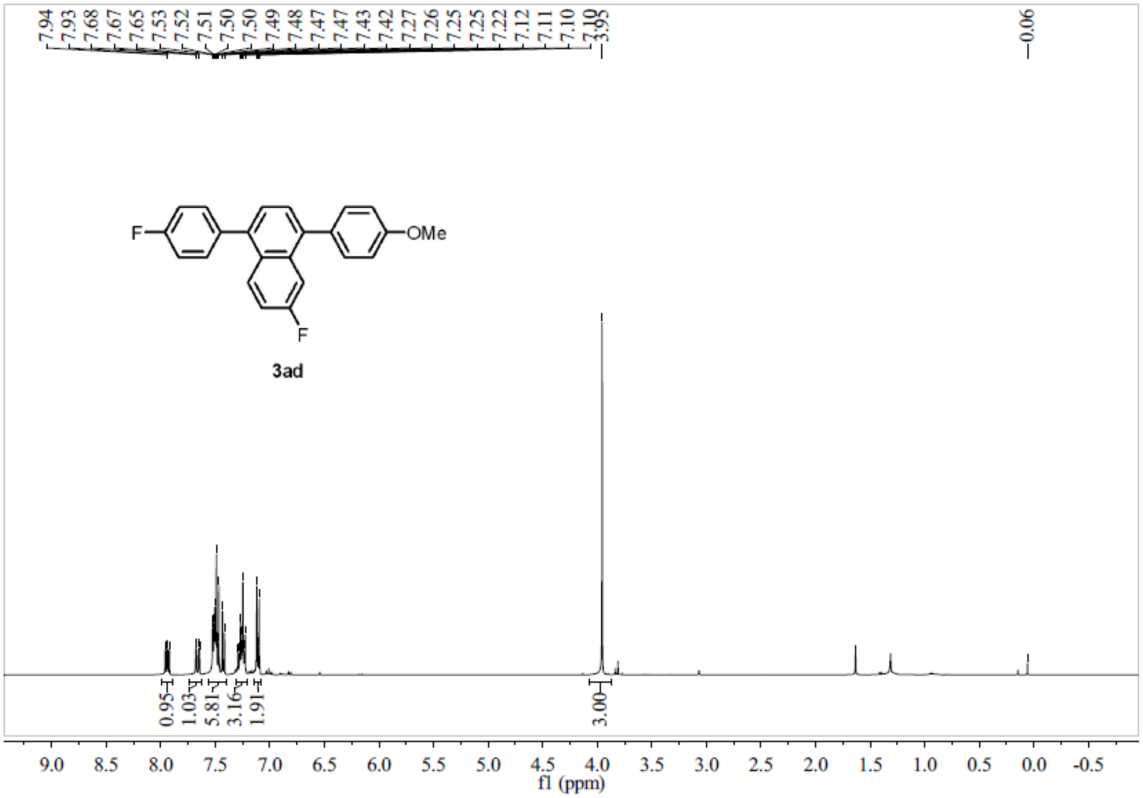

Supplementary Figure 16. ^{1}H NMR of **3ad**

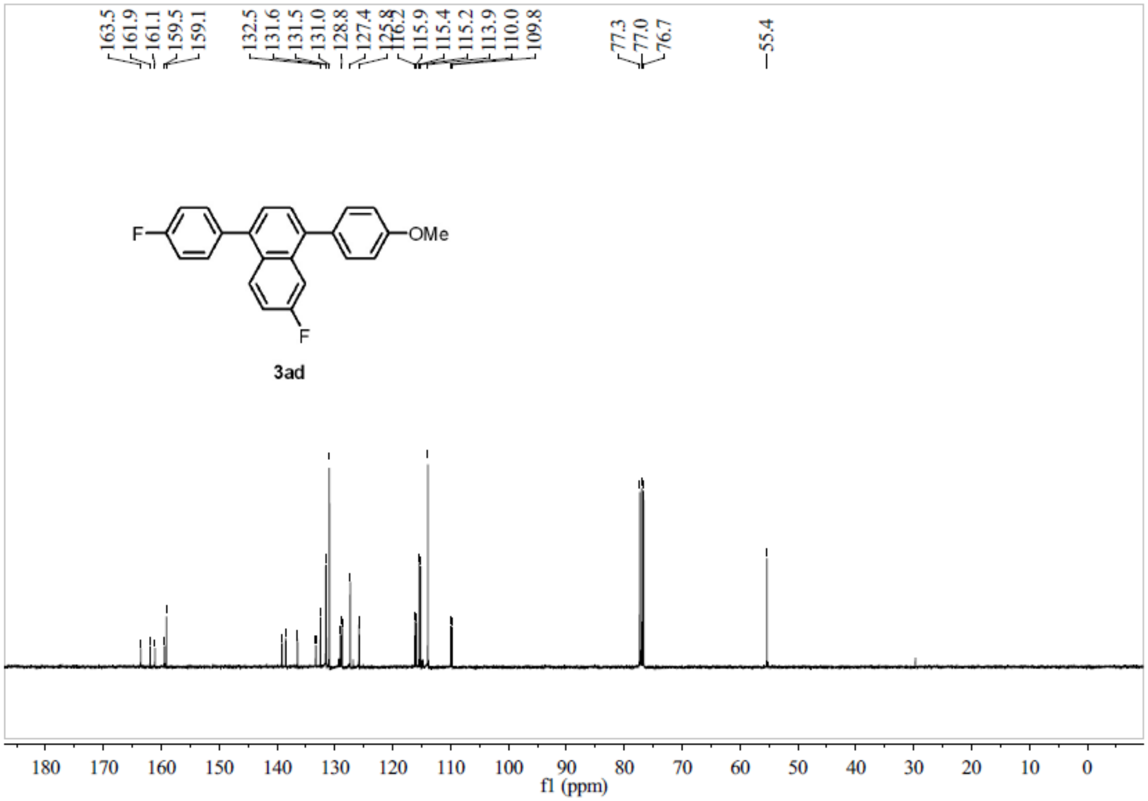

Supplementary Figure 17. ^{13}C NMR of **3ad**

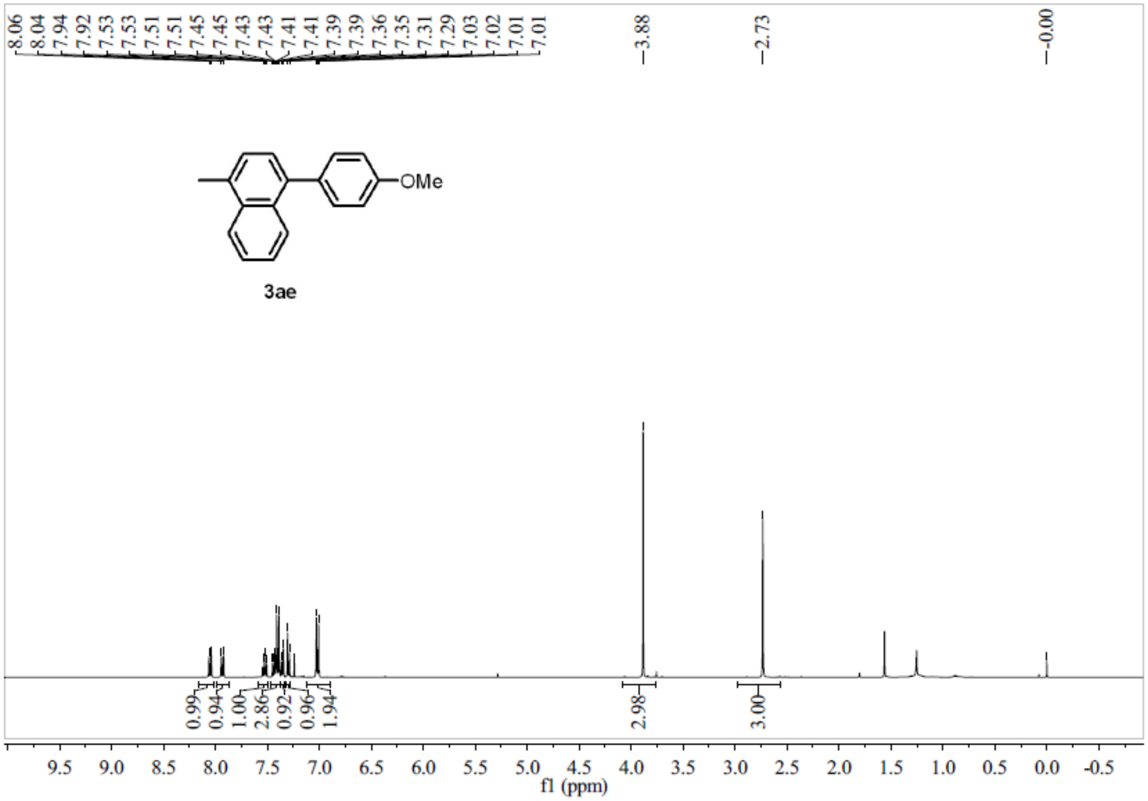

Supplementary Figure 18. ^{1}H NMR of **3ae**

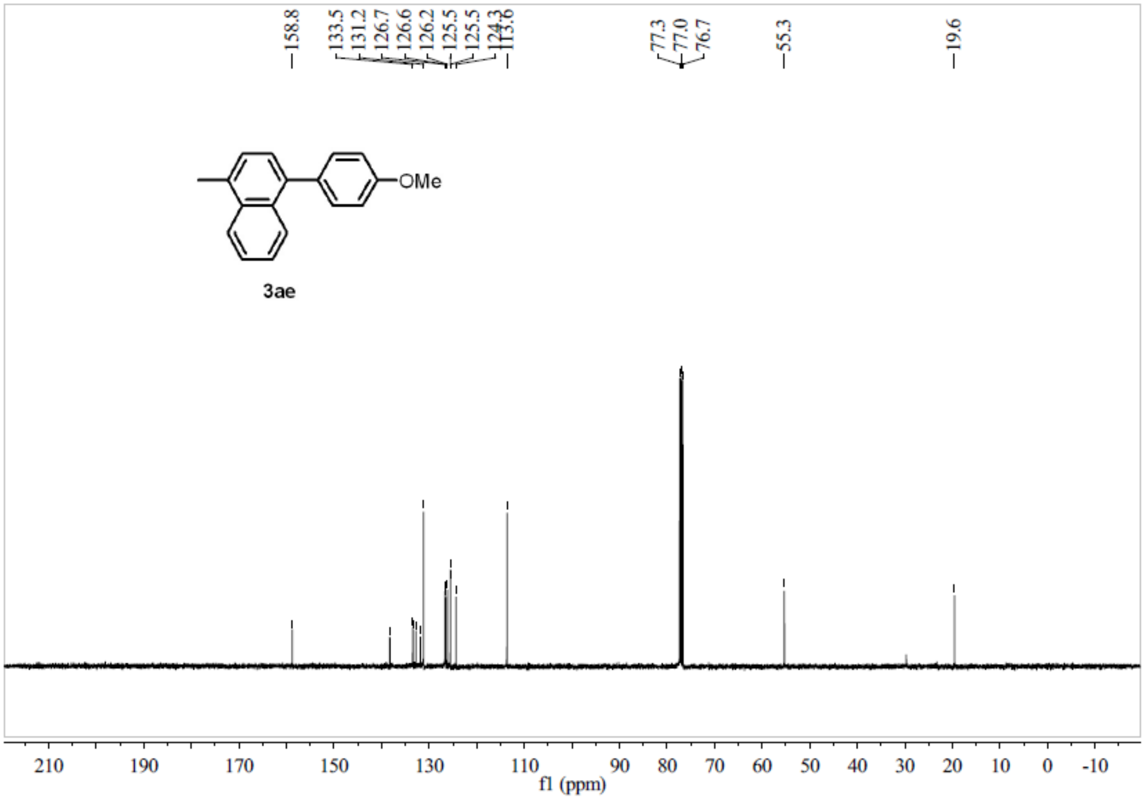

Supplementary Figure 19. ^{13}C NMR of **3ae**

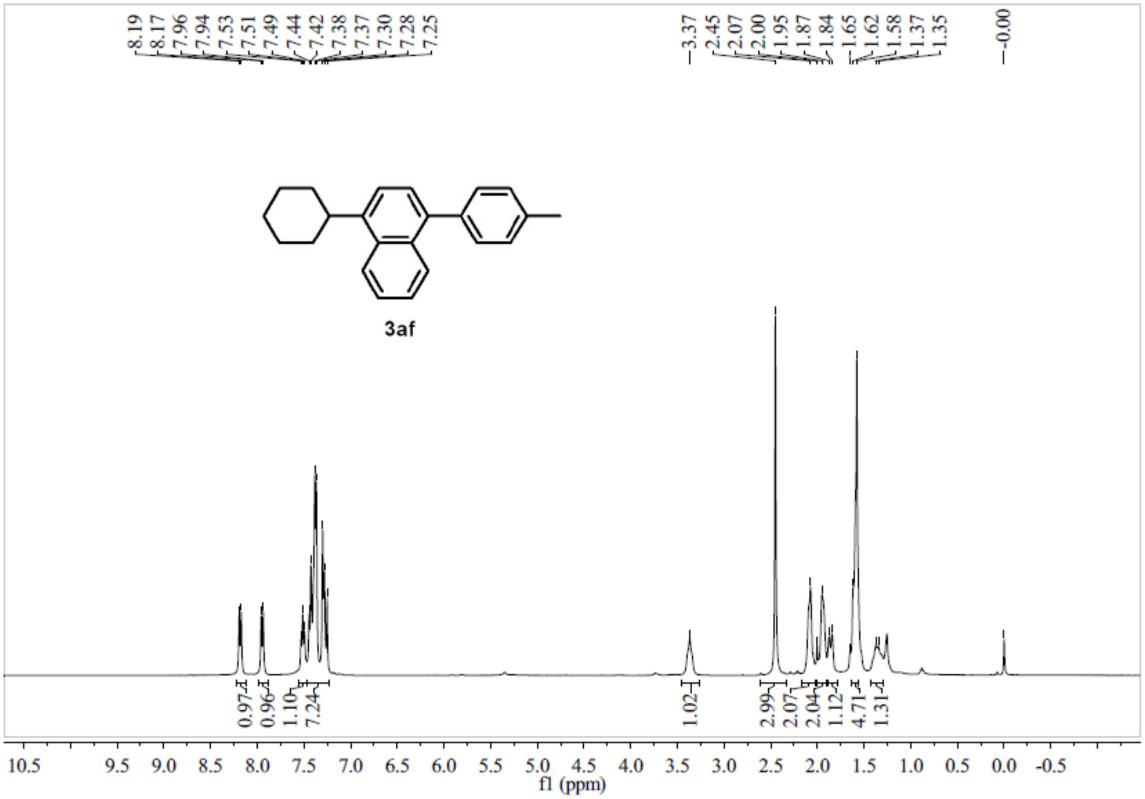

Supplementary Figure 20. ^{1}H NMR of **3af**

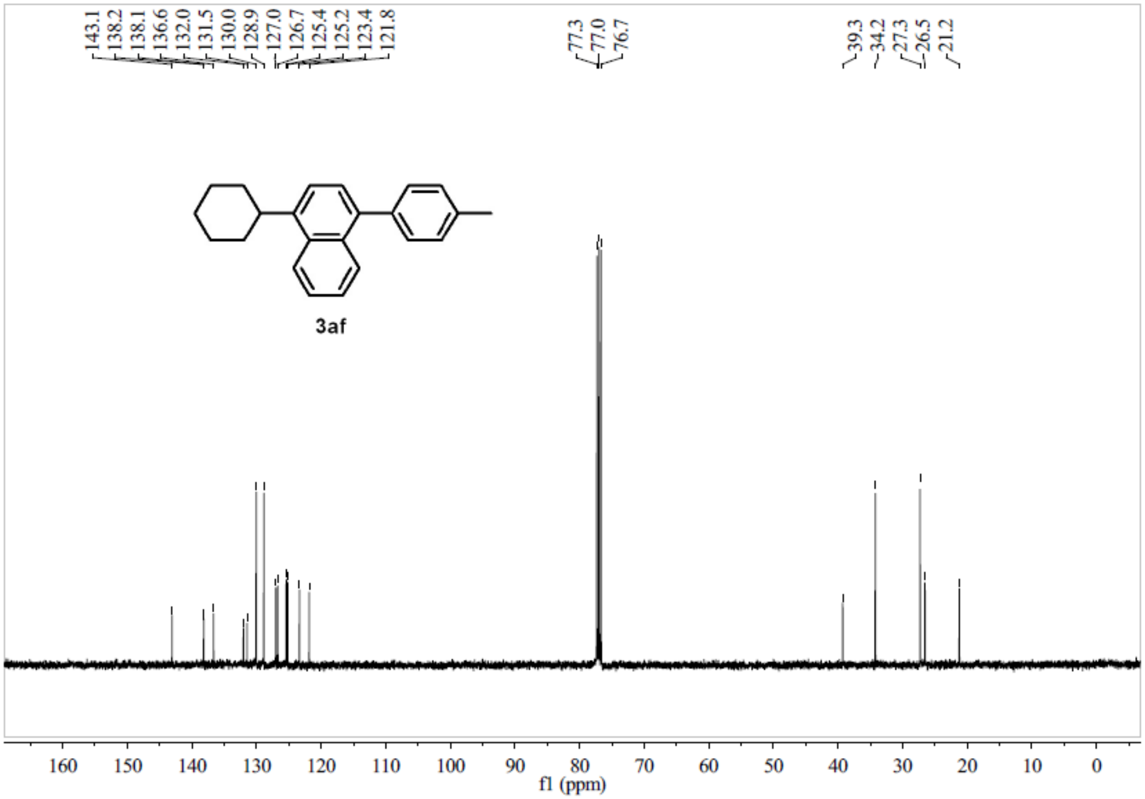

Supplementary Figure 21. ^{13}C NMR of **3af**

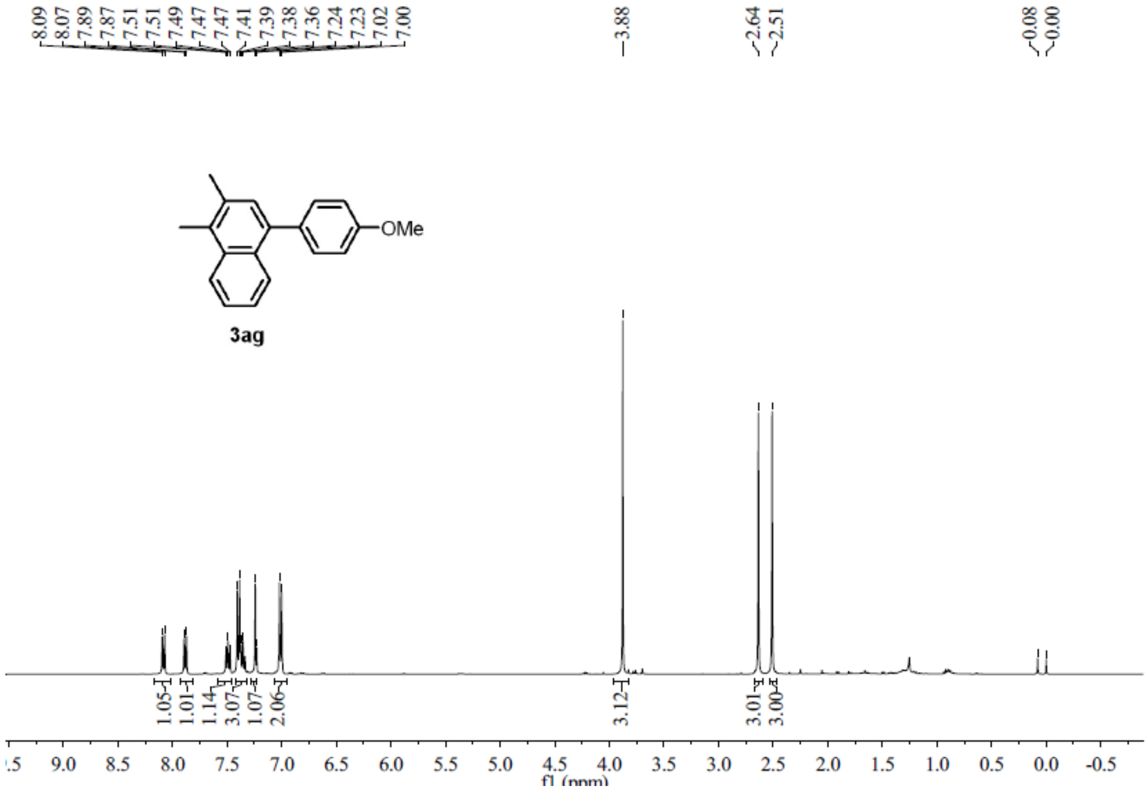

Supplementary Figure 22. ^{1}H NMR of **3ag**

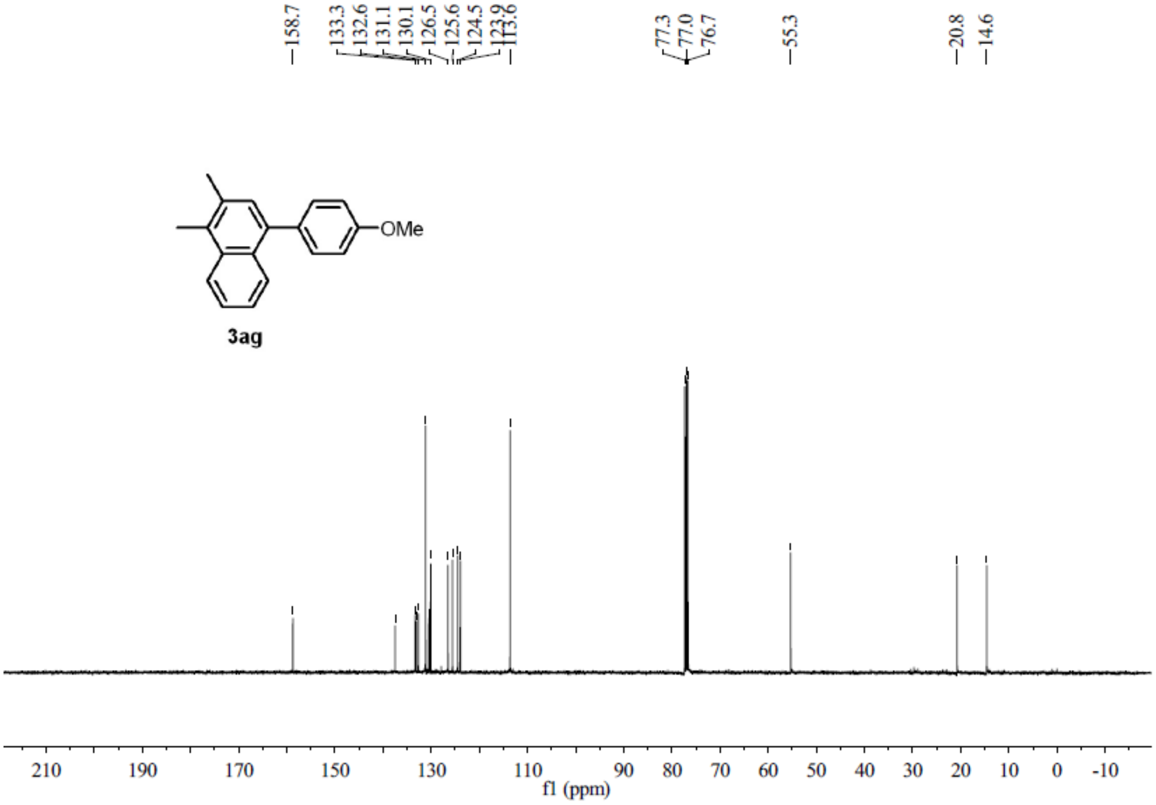

Supplementary Figure 23. ^{13}C NMR of **3ag**

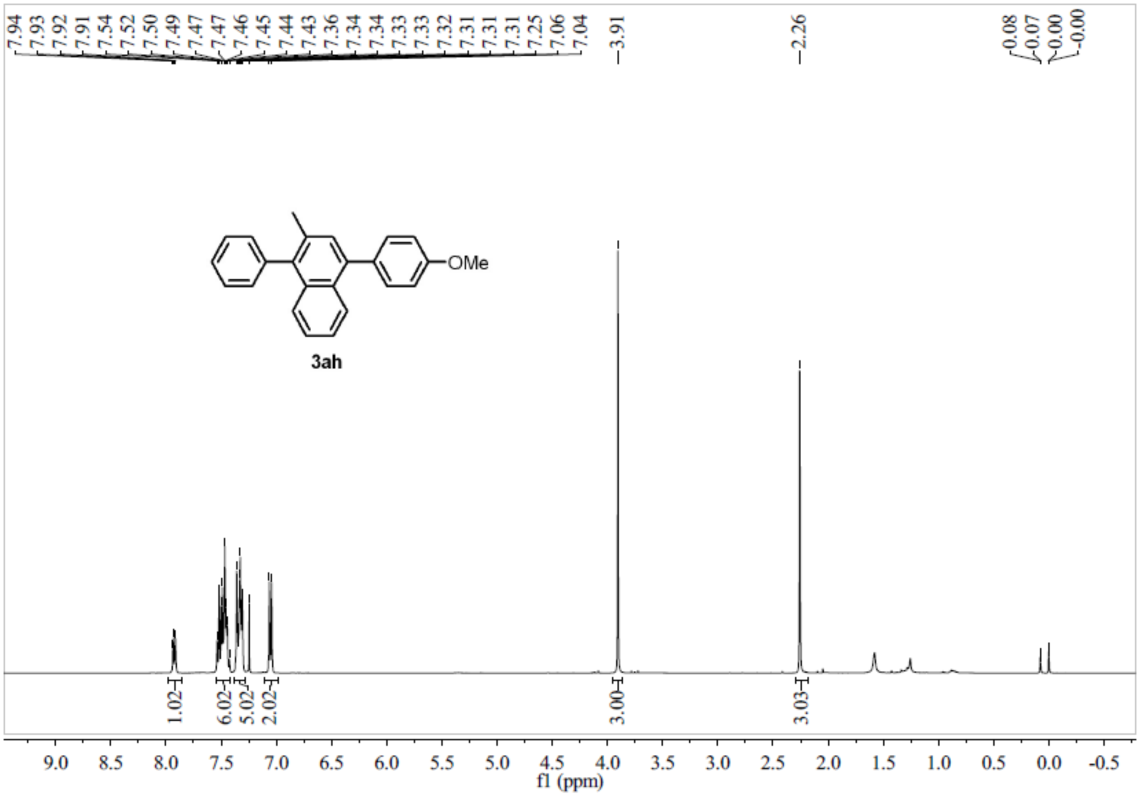

Supplementary Figure 24. ^{1}H NMR of **3ah**

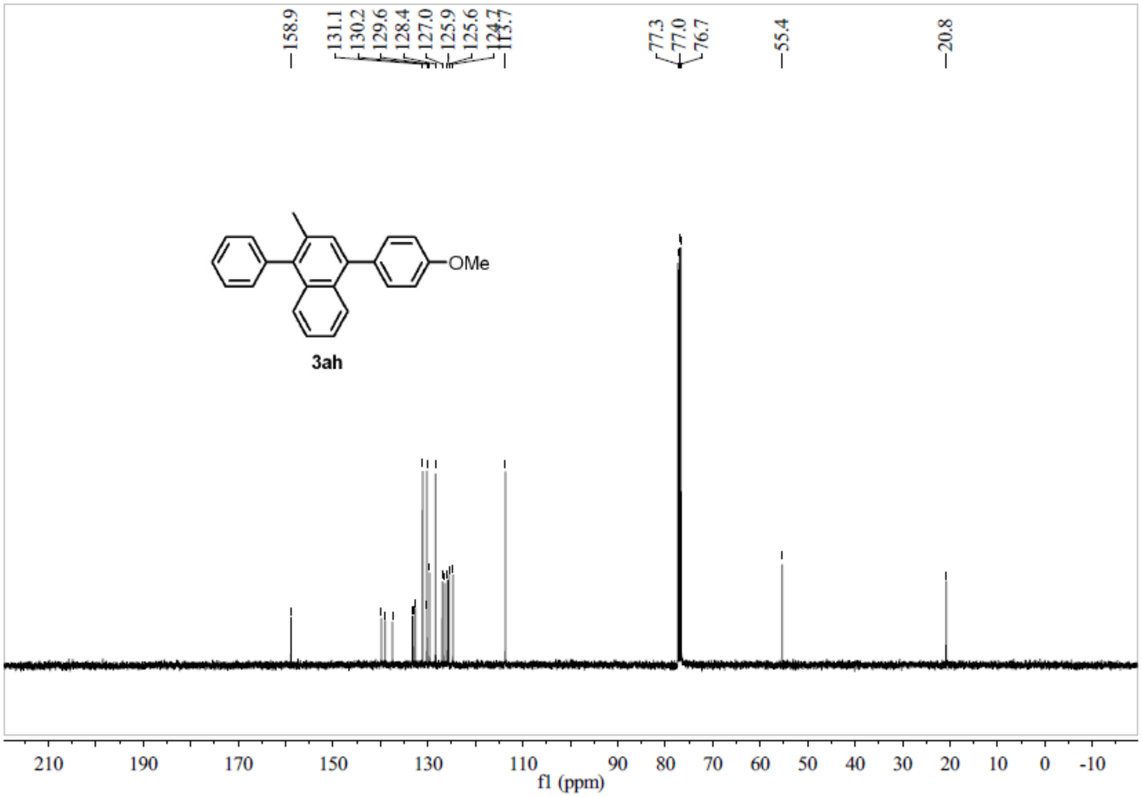

Supplementary Figure 25. ^{13}C NMR of **3ah**

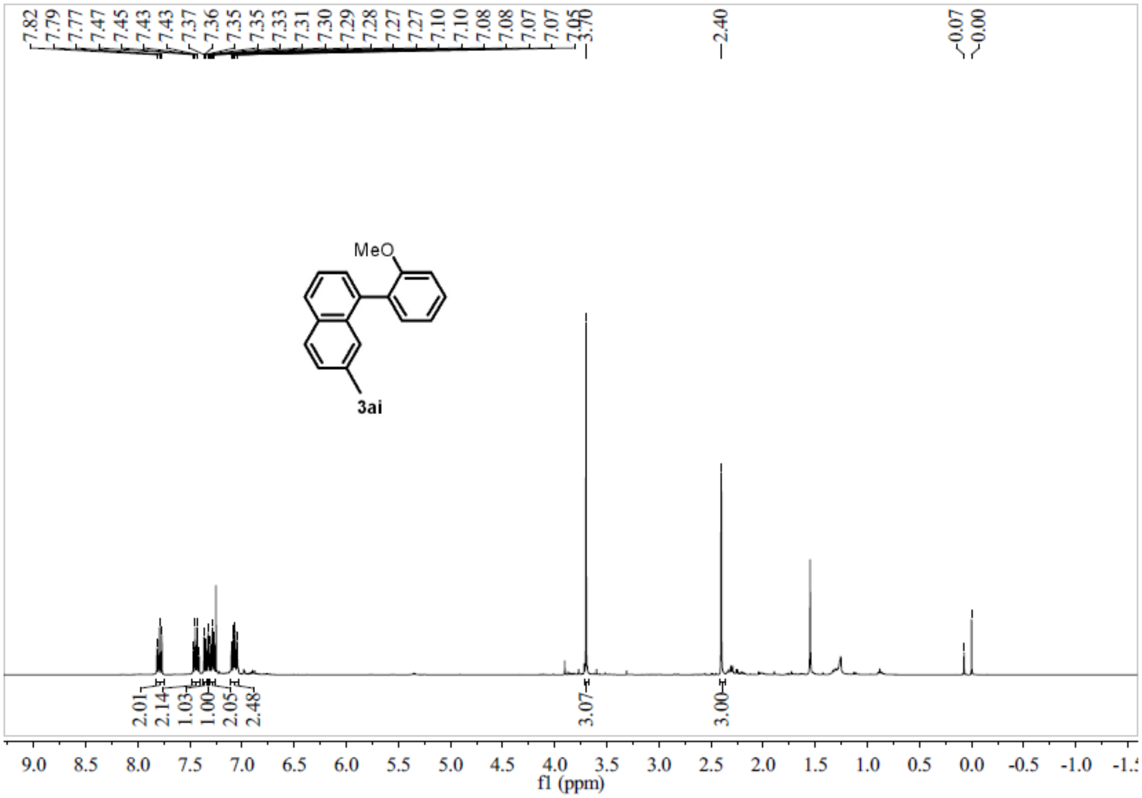

Supplementary Figure 26. ^{1}H NMR of **3ai**

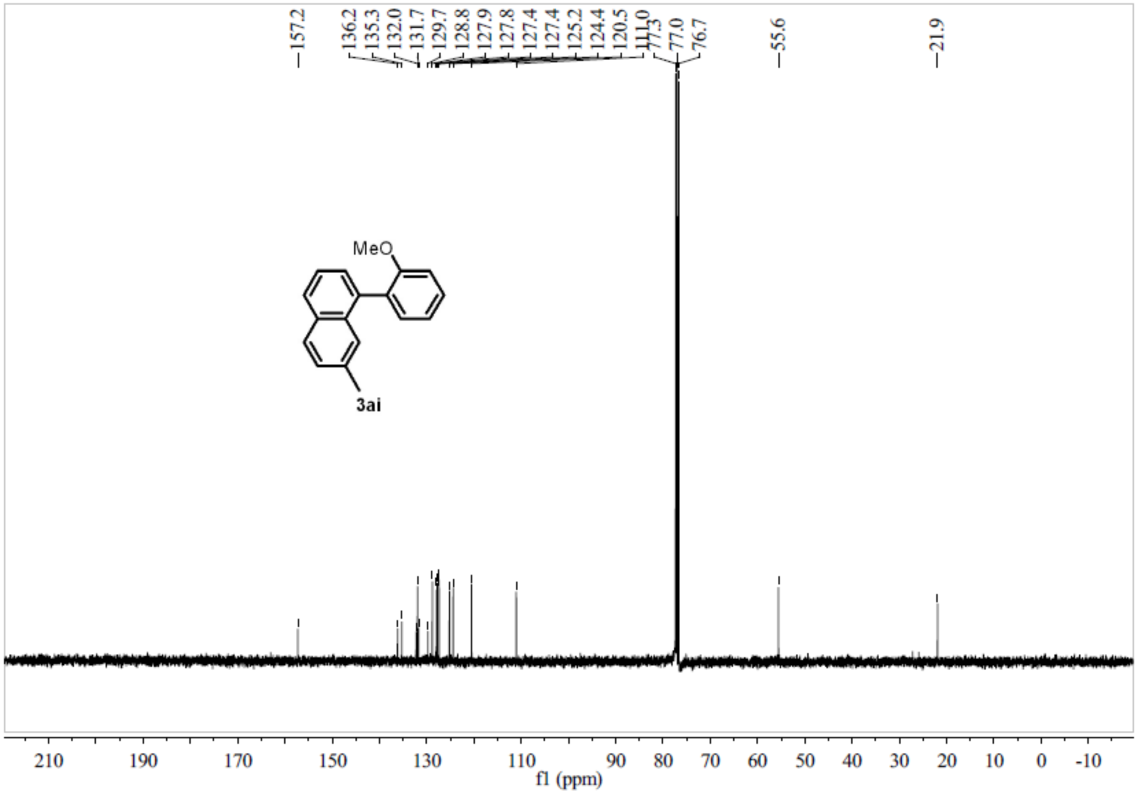

Supplementary Figure 27. ^{13}C NMR of **3ai**

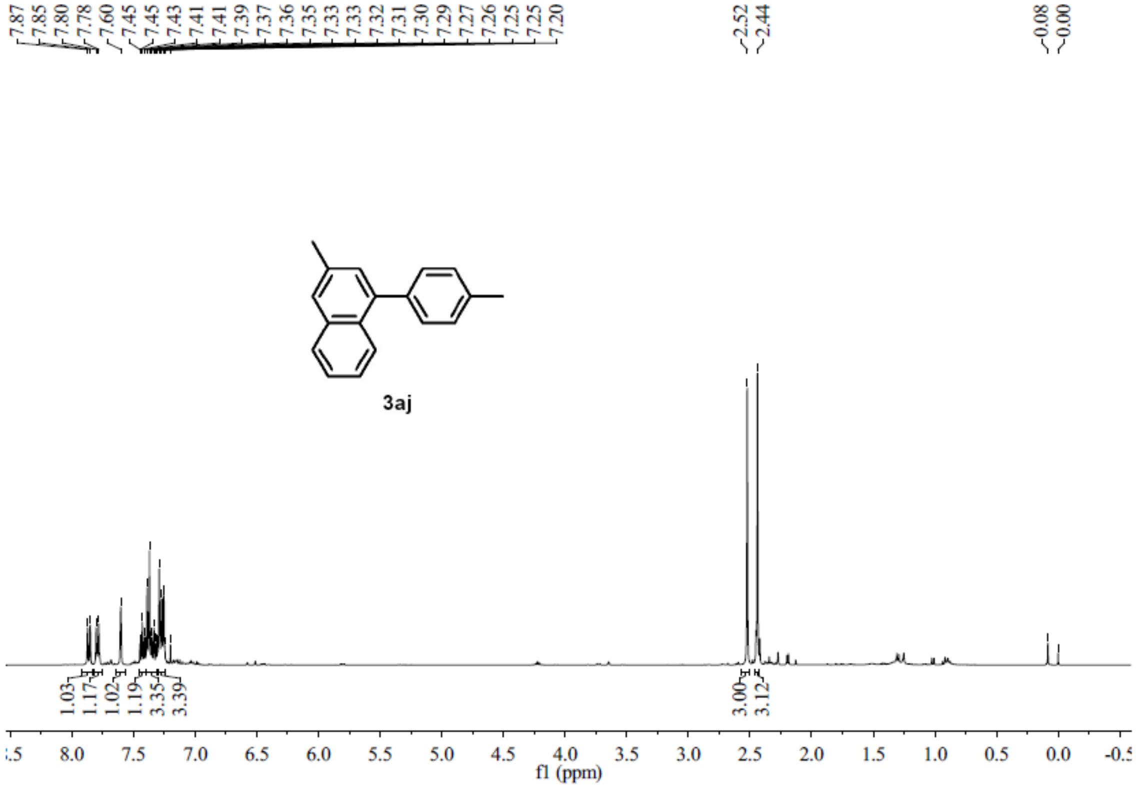

Supplementary Figure 28. ^{1}H NMR of **3aj**

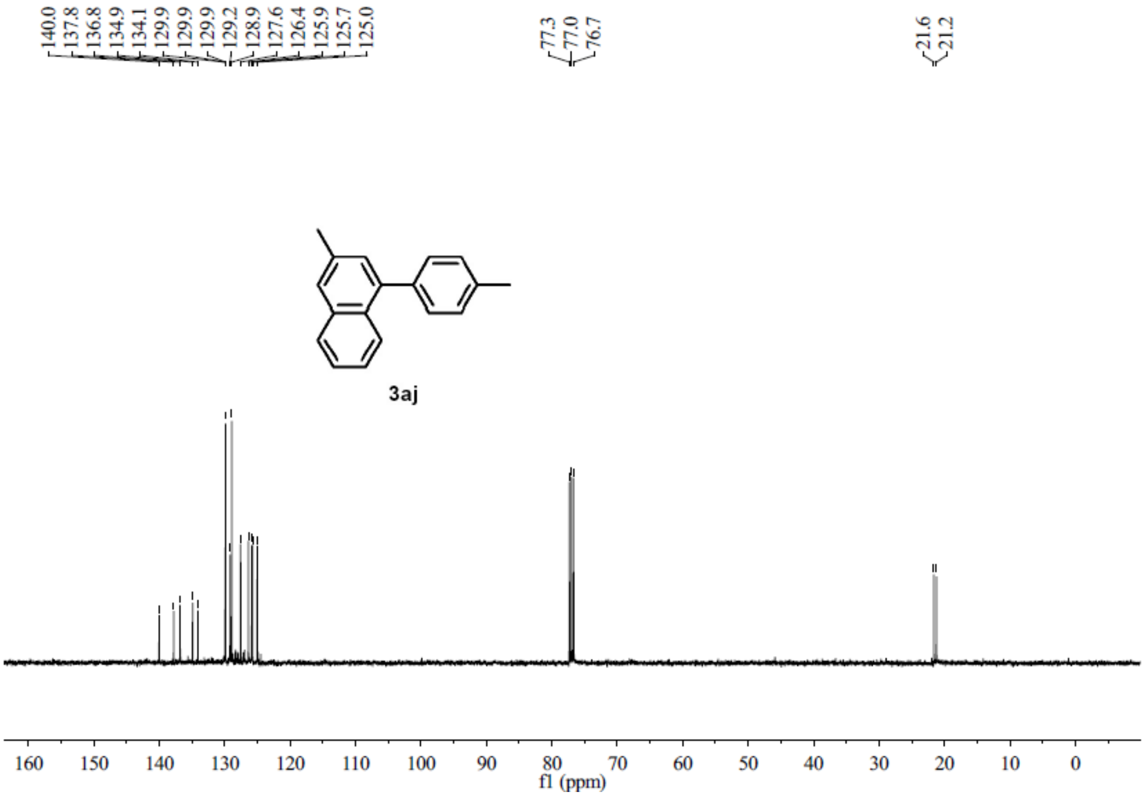

Supplementary Figure 29. ^{13}C NMR of **3aj**

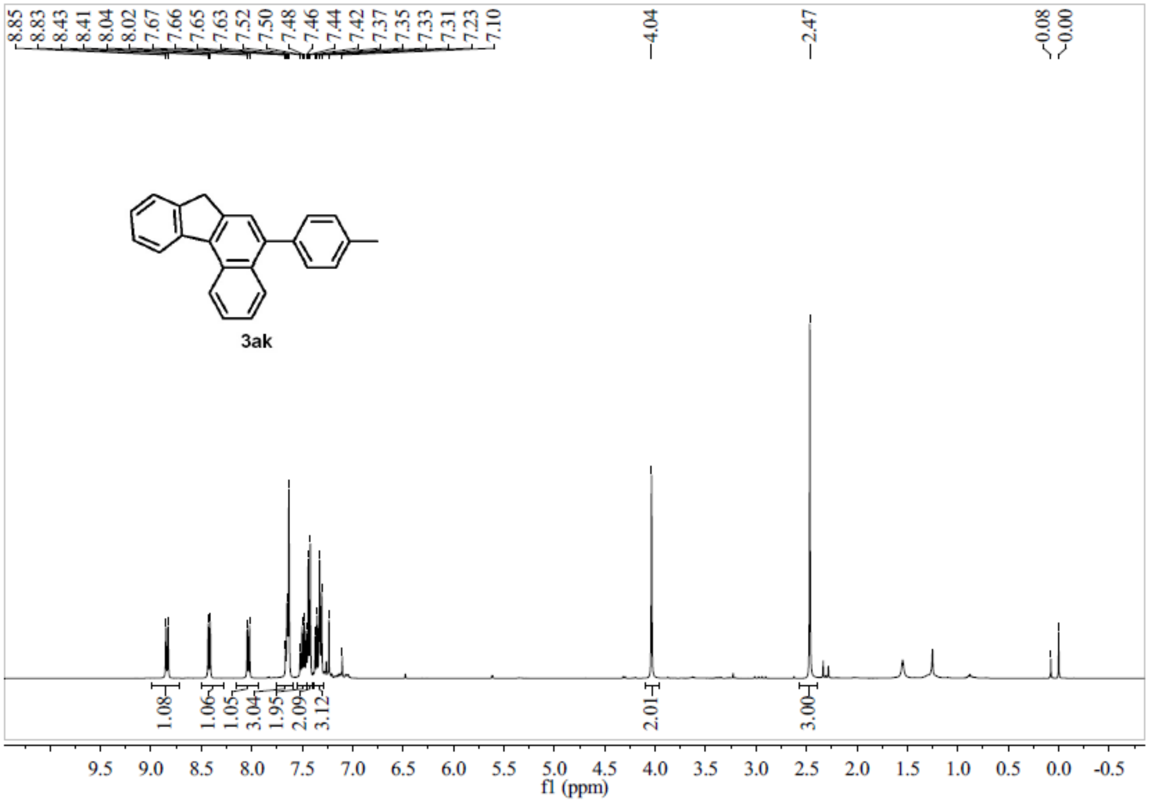

Supplementary Figure 30. ^{1}H NMR of **3ak**

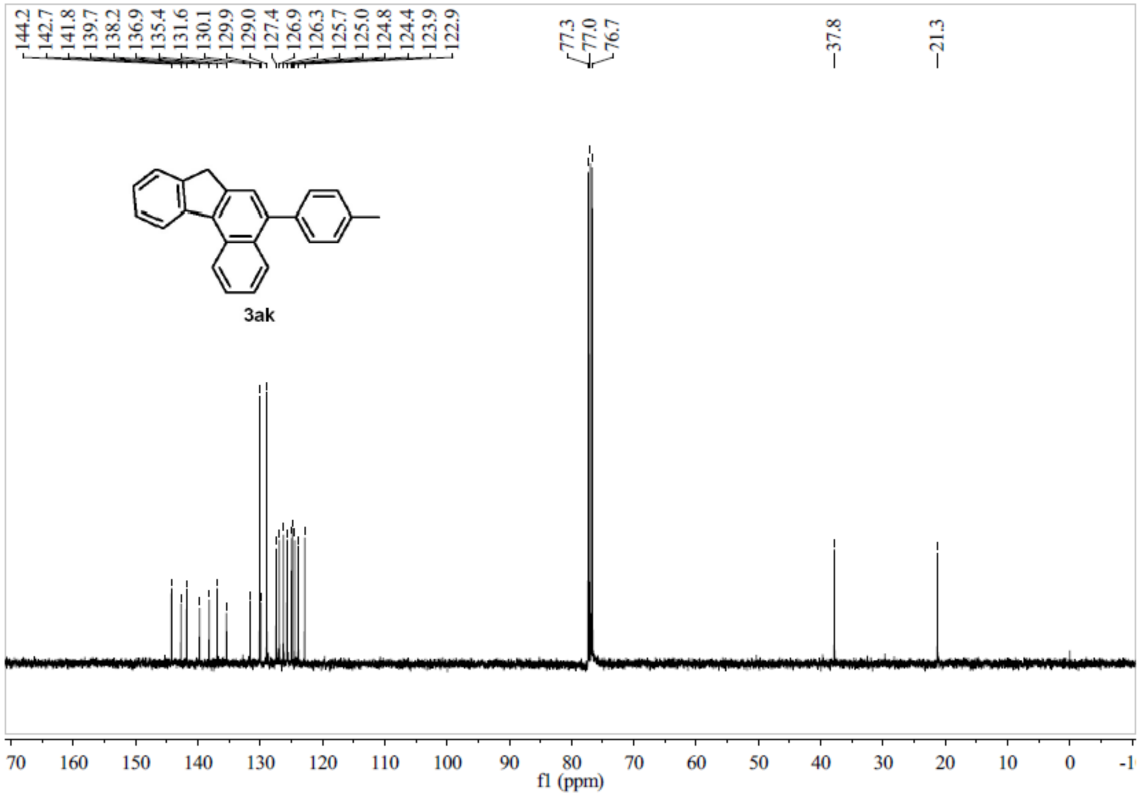

Supplementary Figure 31. ^{13}C NMR of **3ak**

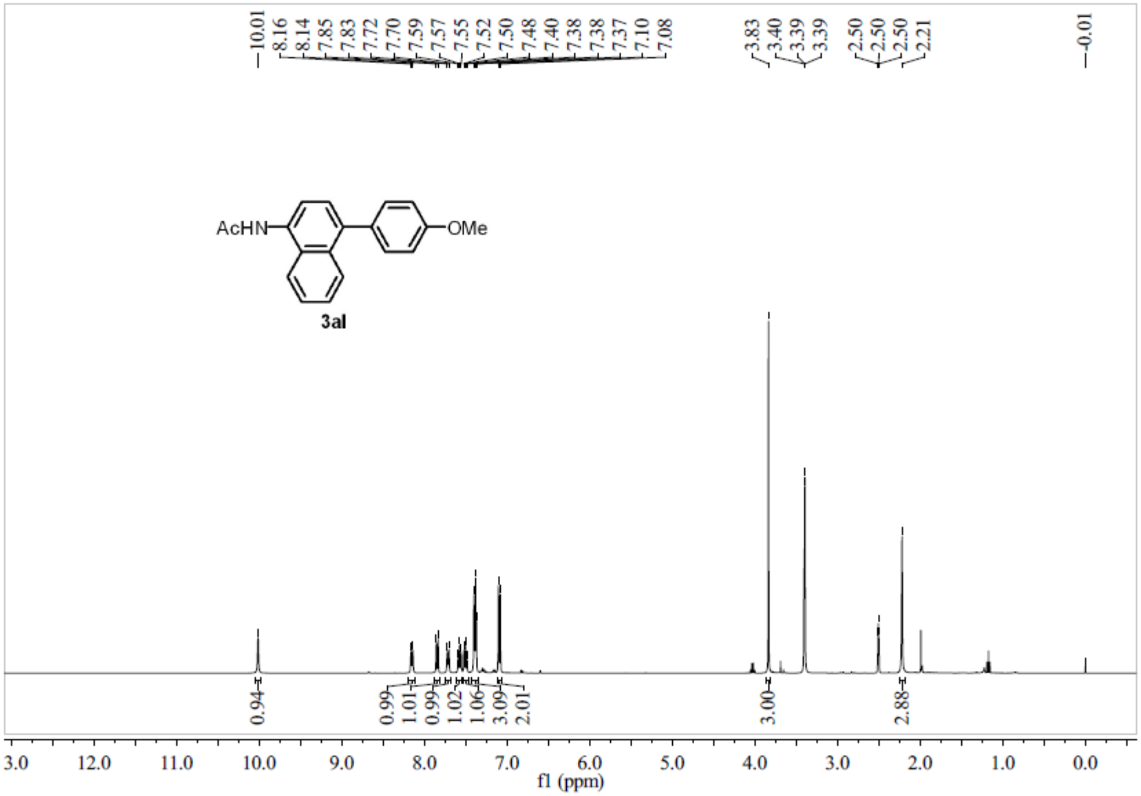

Supplementary Figure 32. ^{1}H NMR of **3al**

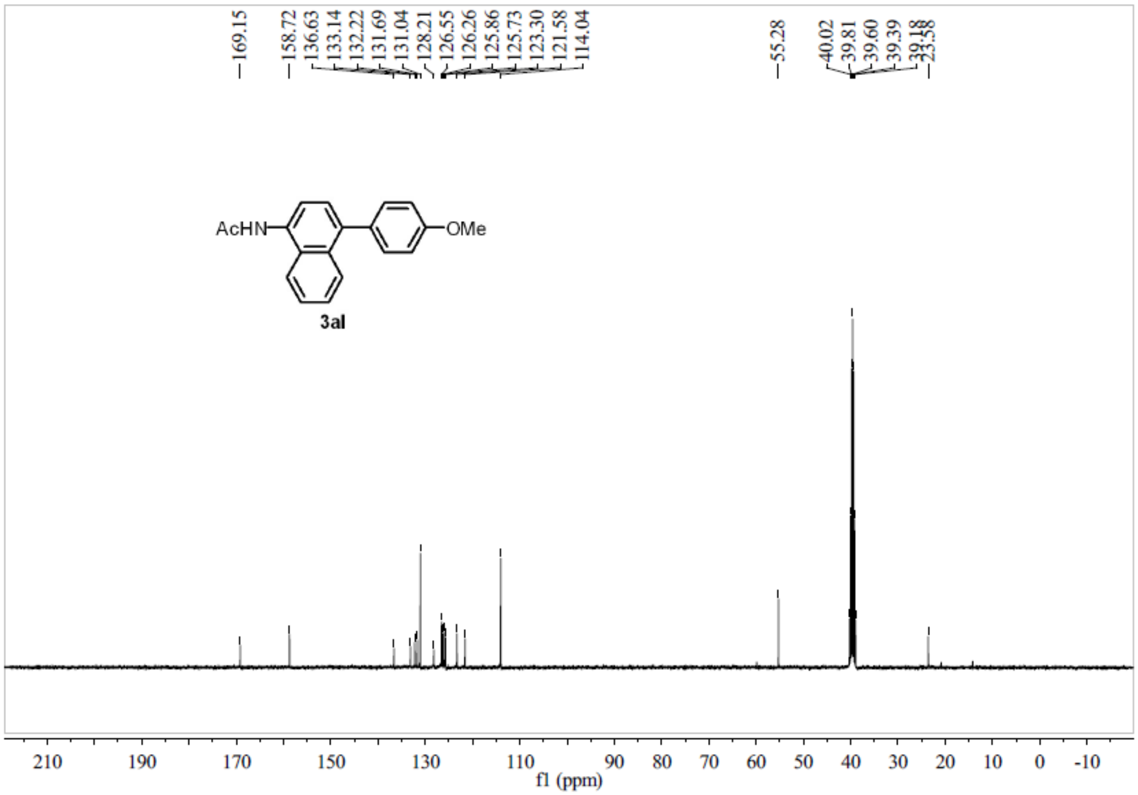

Supplementary Figure 33. ^{13}C NMR of **3al**

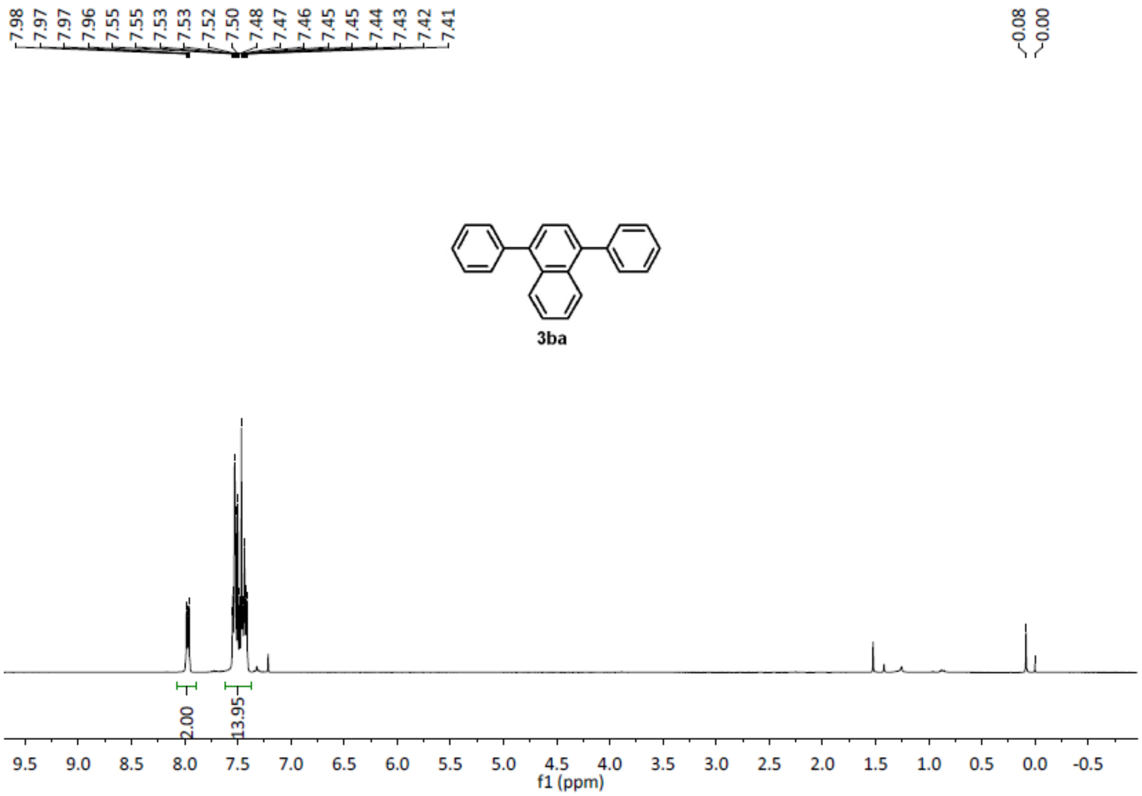

Supplementary Figure 34. ^{1}H NMR of **3ba**

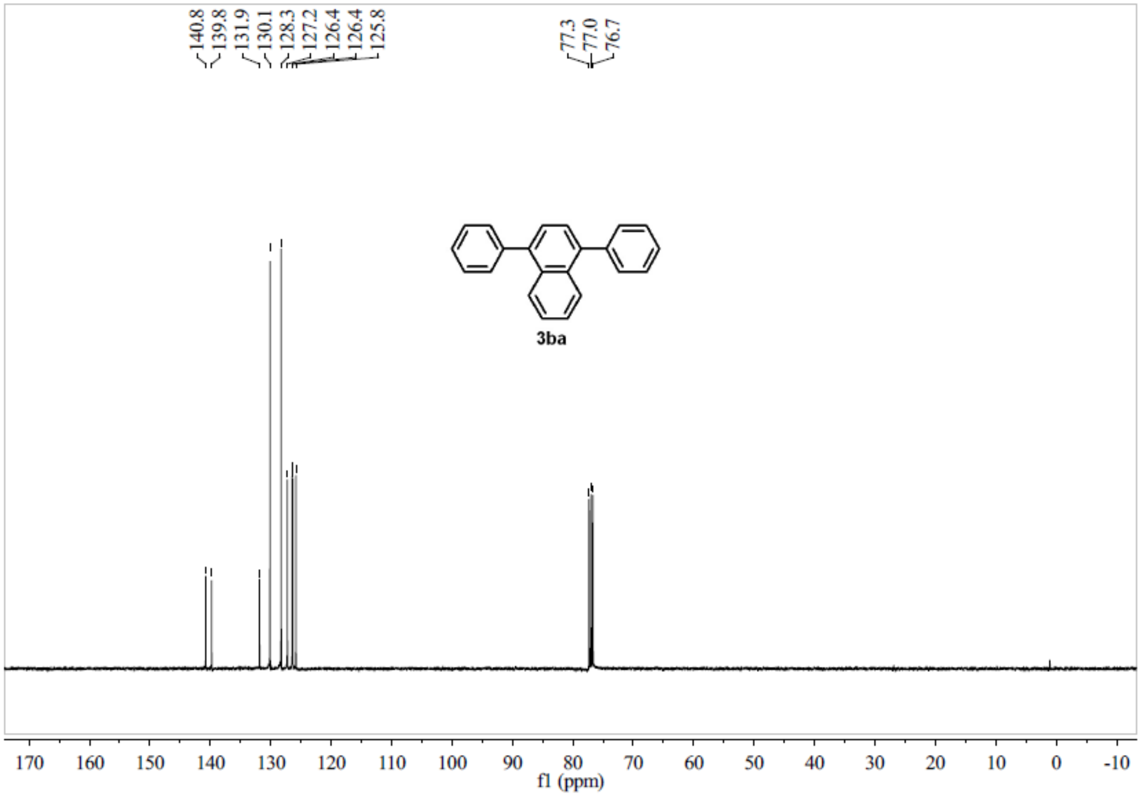

Supplementary Figure 35. ^{13}C NMR of **3ba**

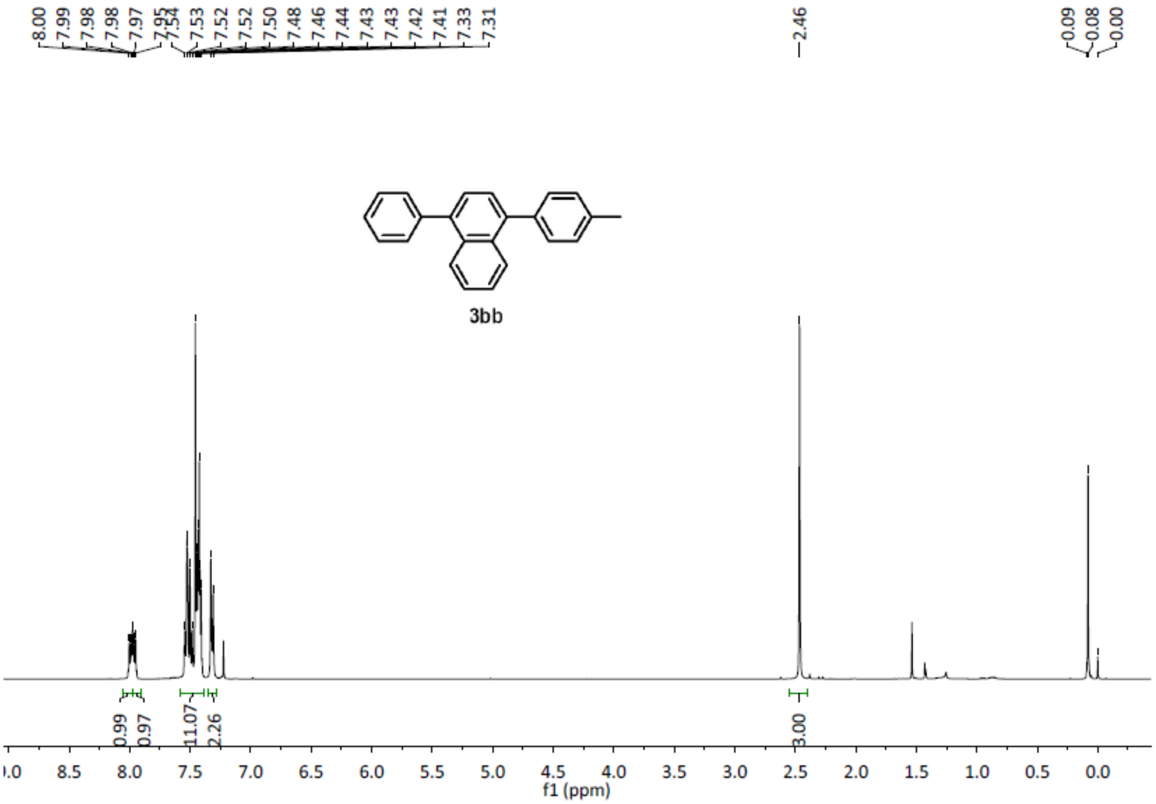

Supplementary Figure 36. ^{1}H NMR of **3bb**

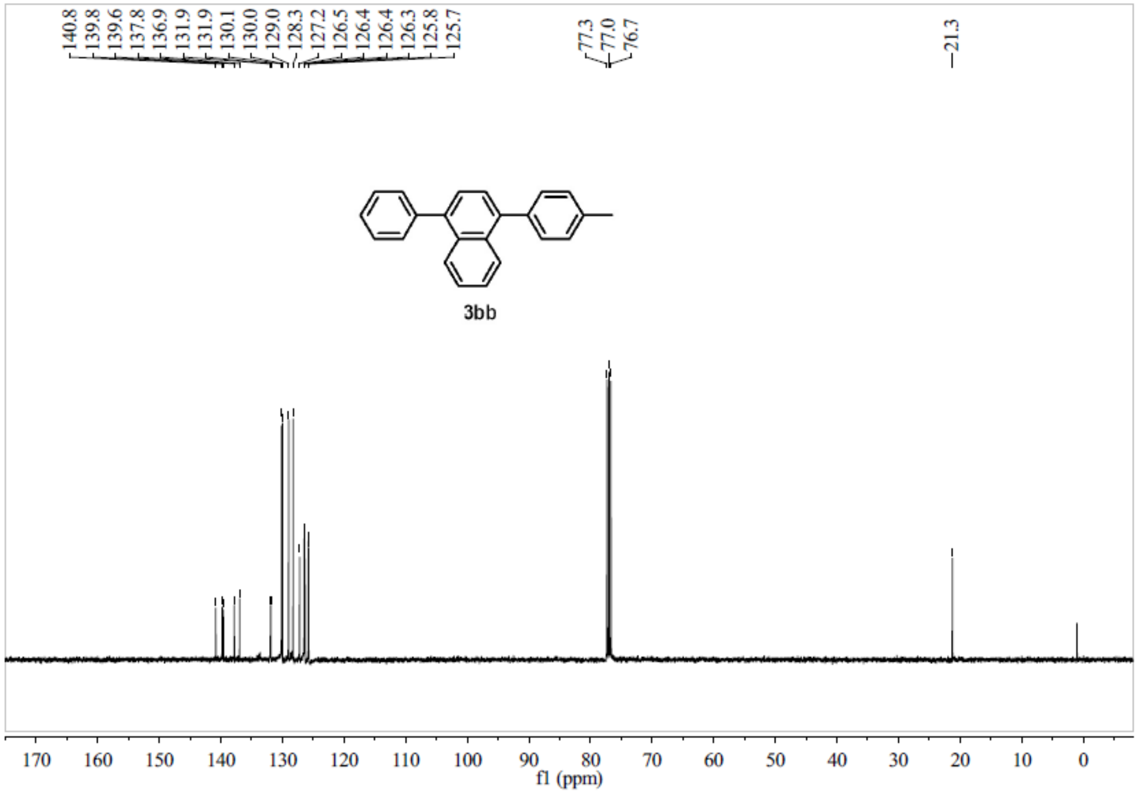

Supplementary Figure 37. ^{13}C NMR of **3bb**

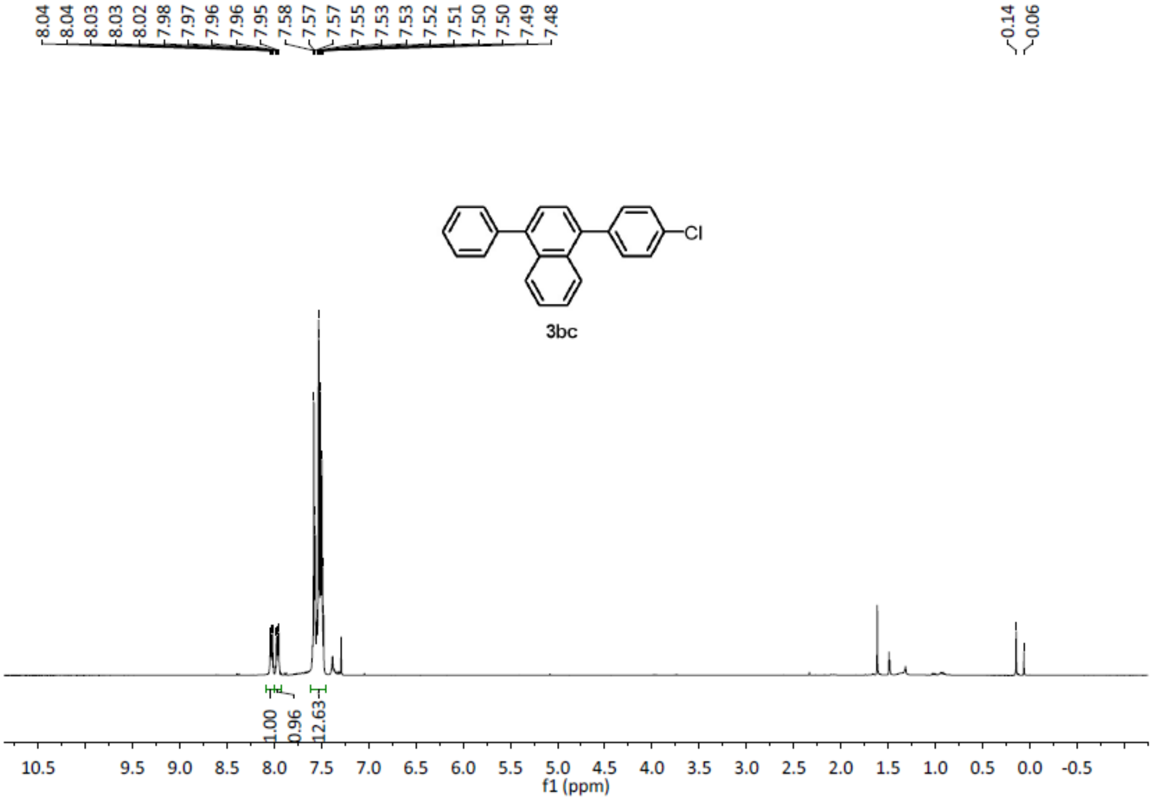

Supplementary Figure 38. ^{1}H NMR of **3bc**

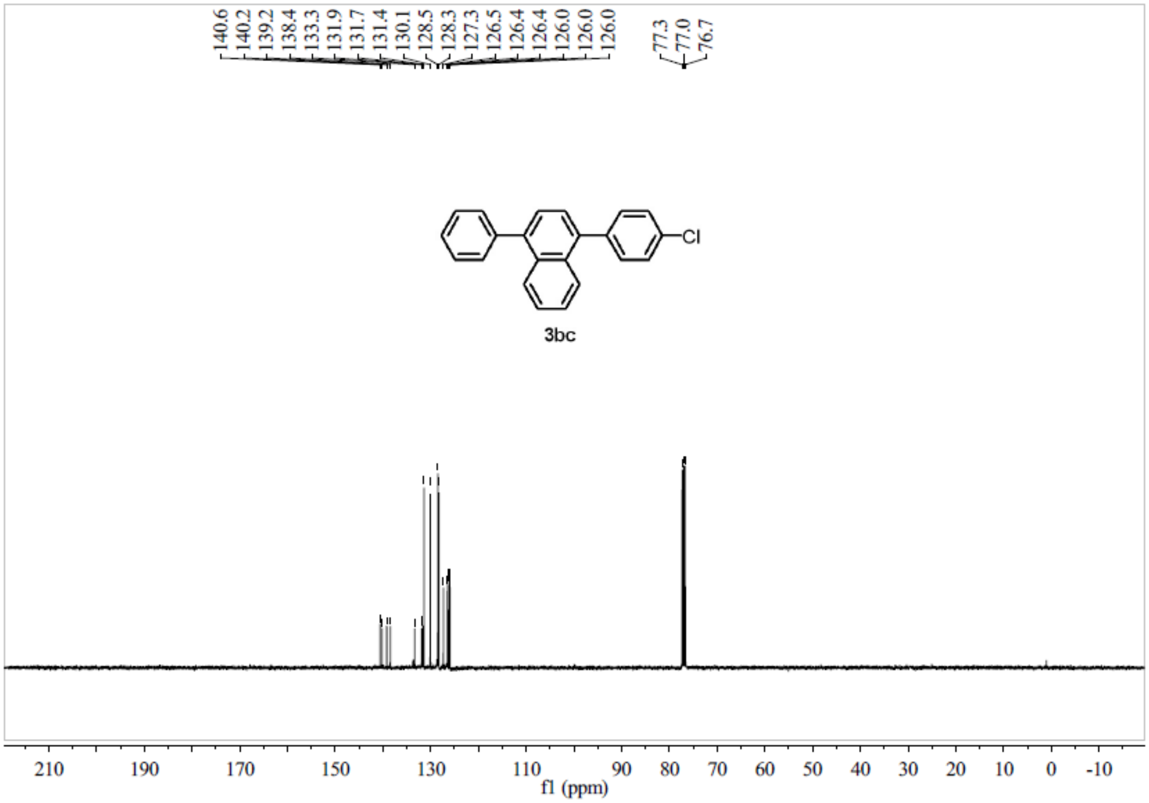

Supplementary Figure 39. ^{13}C NMR of **3bc**

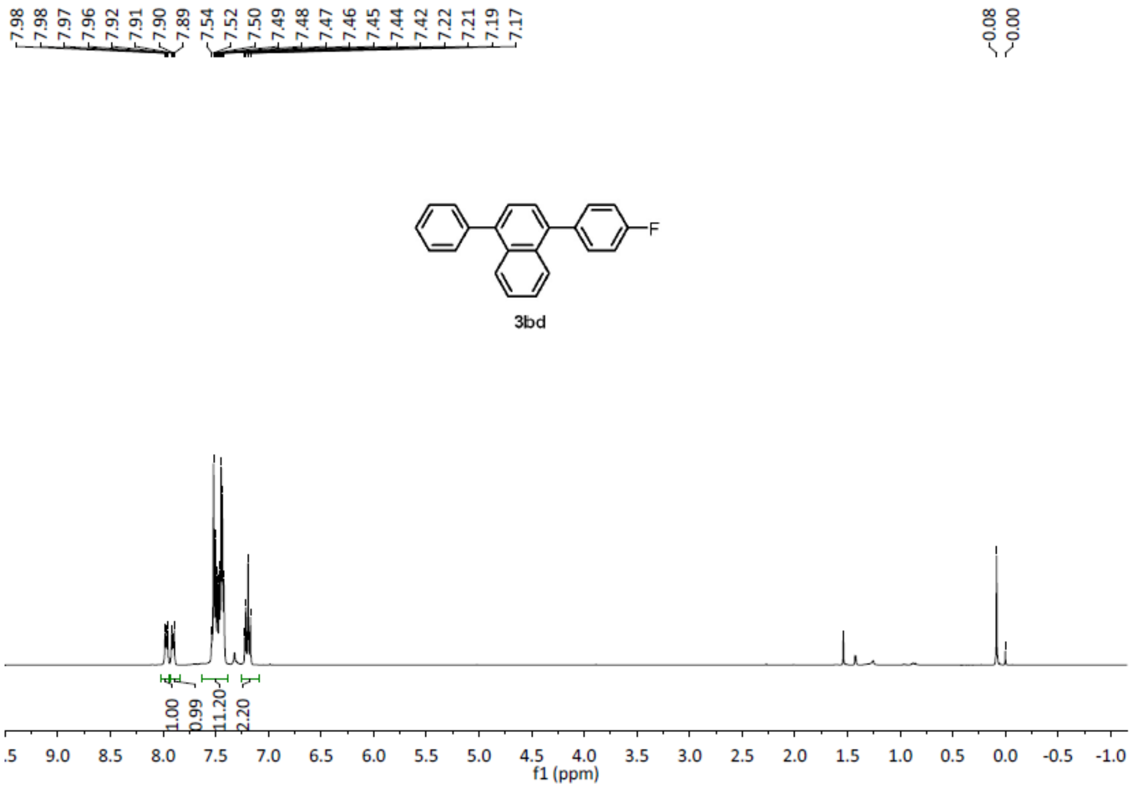

Supplementary Figure 40. ^{1}H NMR of **3bd**

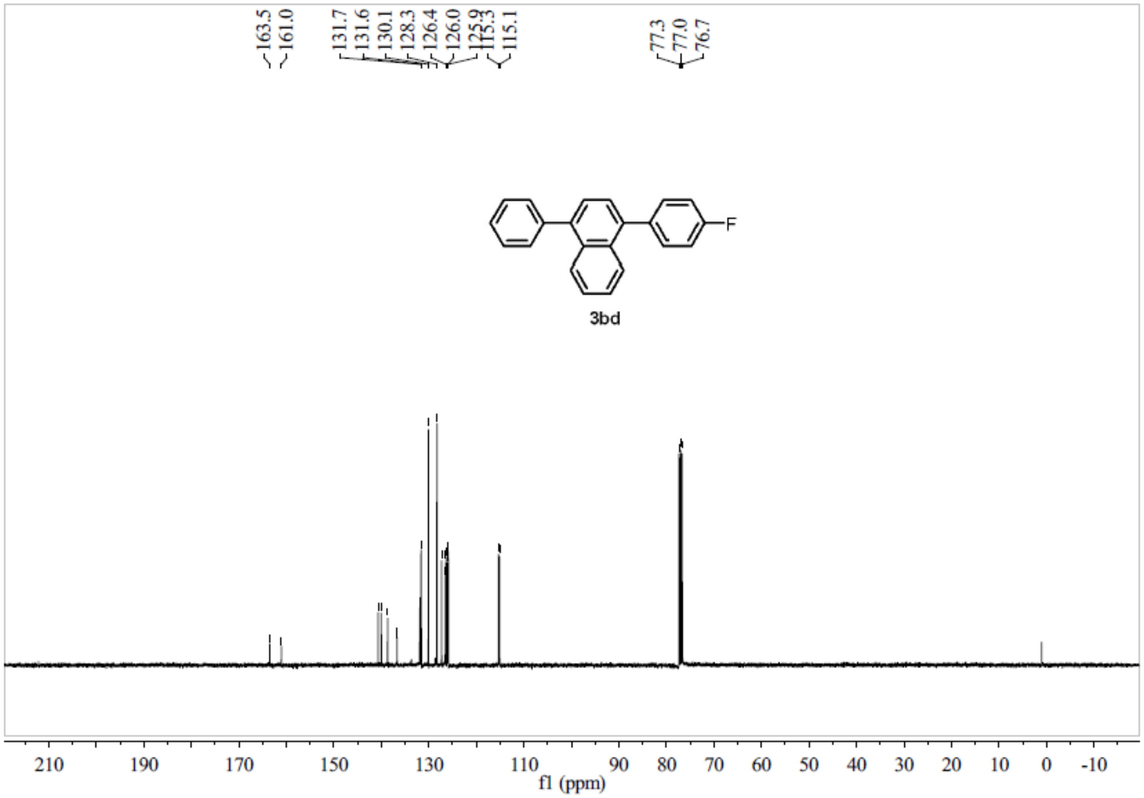

Supplementary Figure 41. ^{13}C NMR of **3bd**

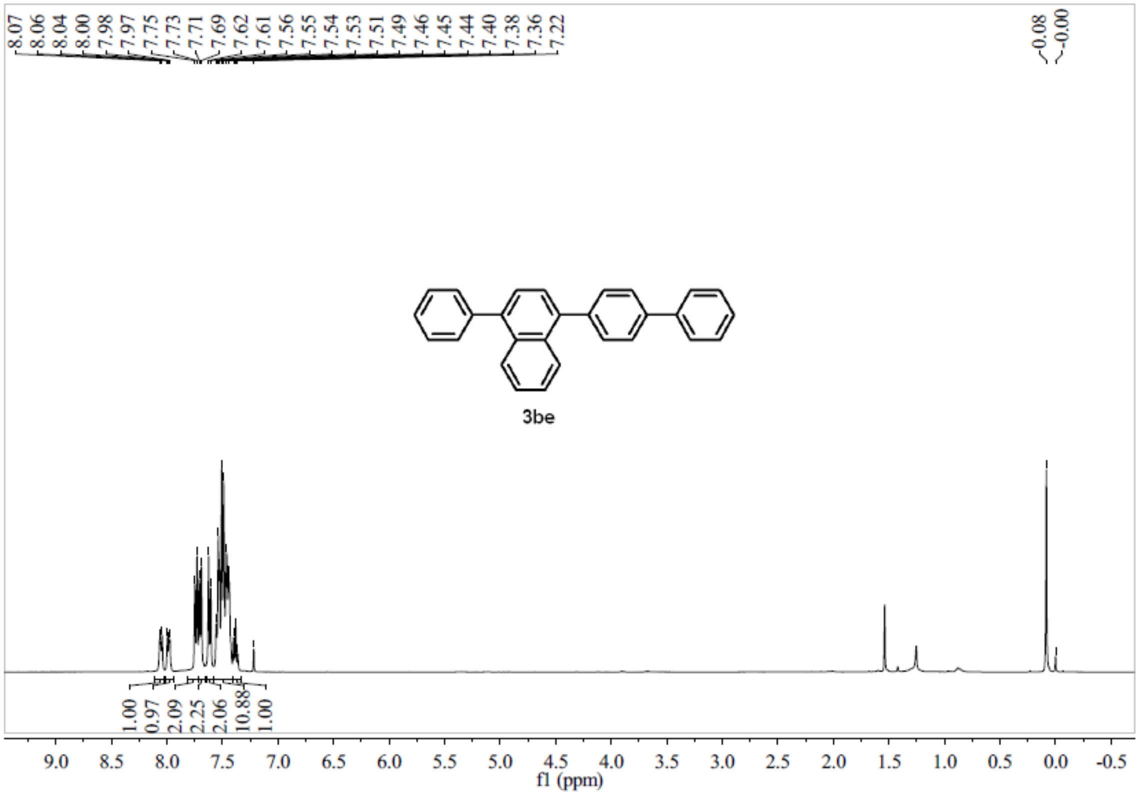

Supplementary Figure 42. ^{1}H NMR of **3be**

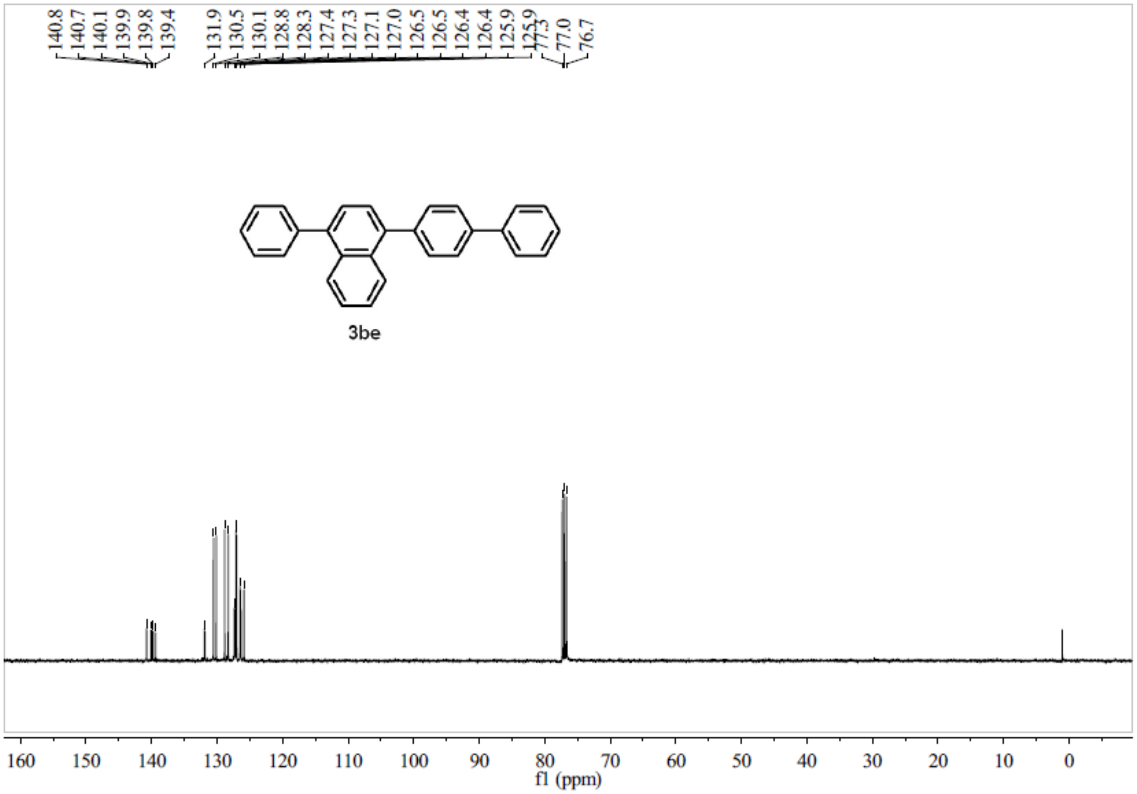

Supplementary Figure 43. ^{13}C NMR of **3be**

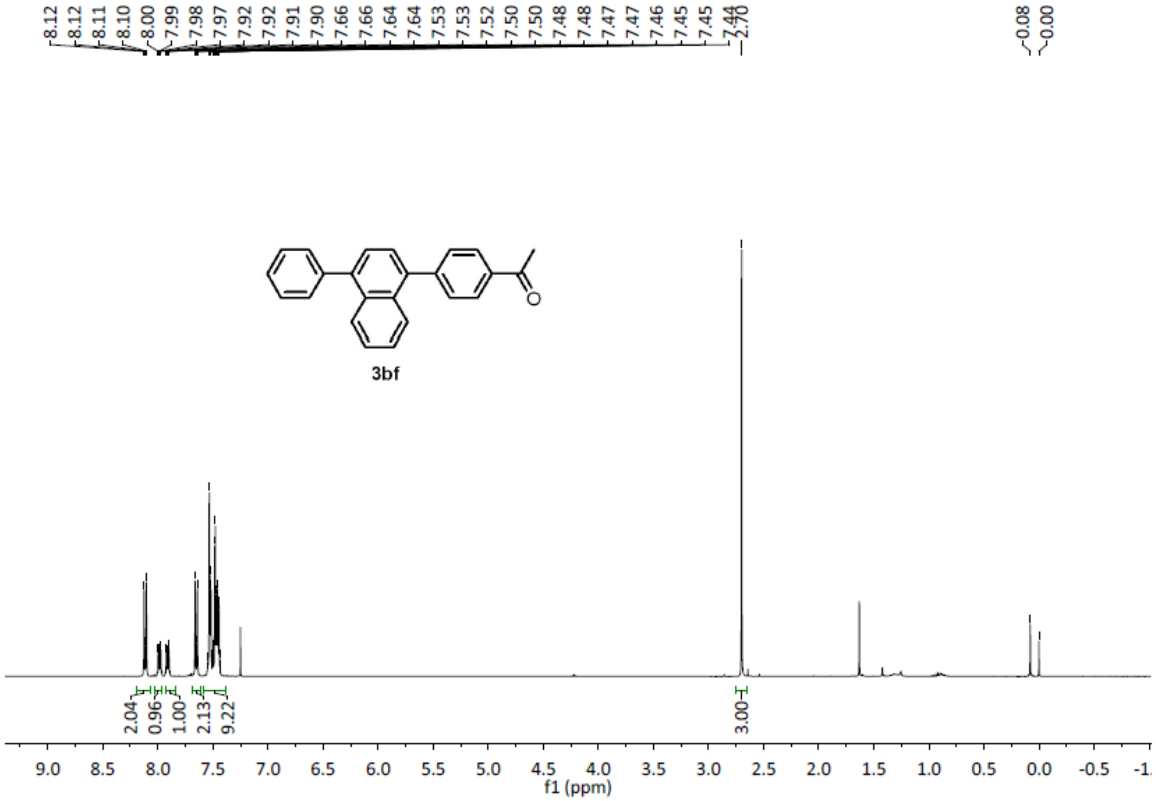

Supplementary Figure 44. ^{1}H NMR of **3bf**

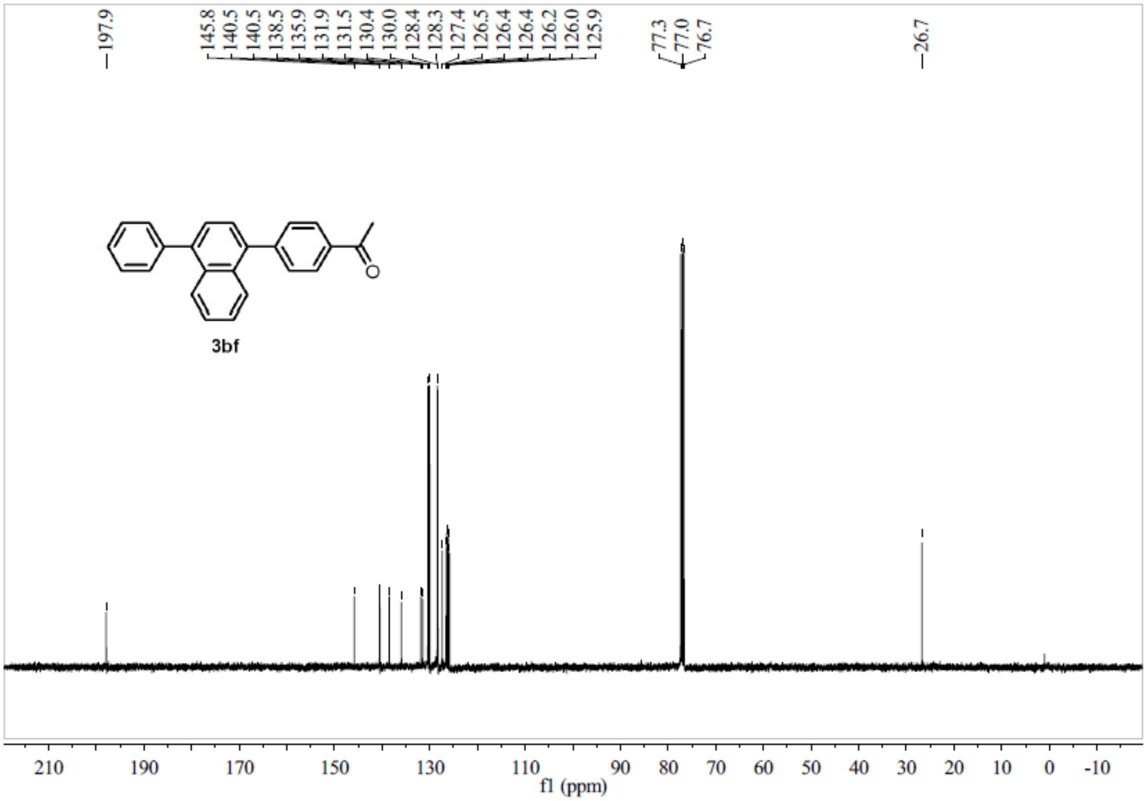

Supplementary Figure 45. ^{13}C NMR of **3bf**

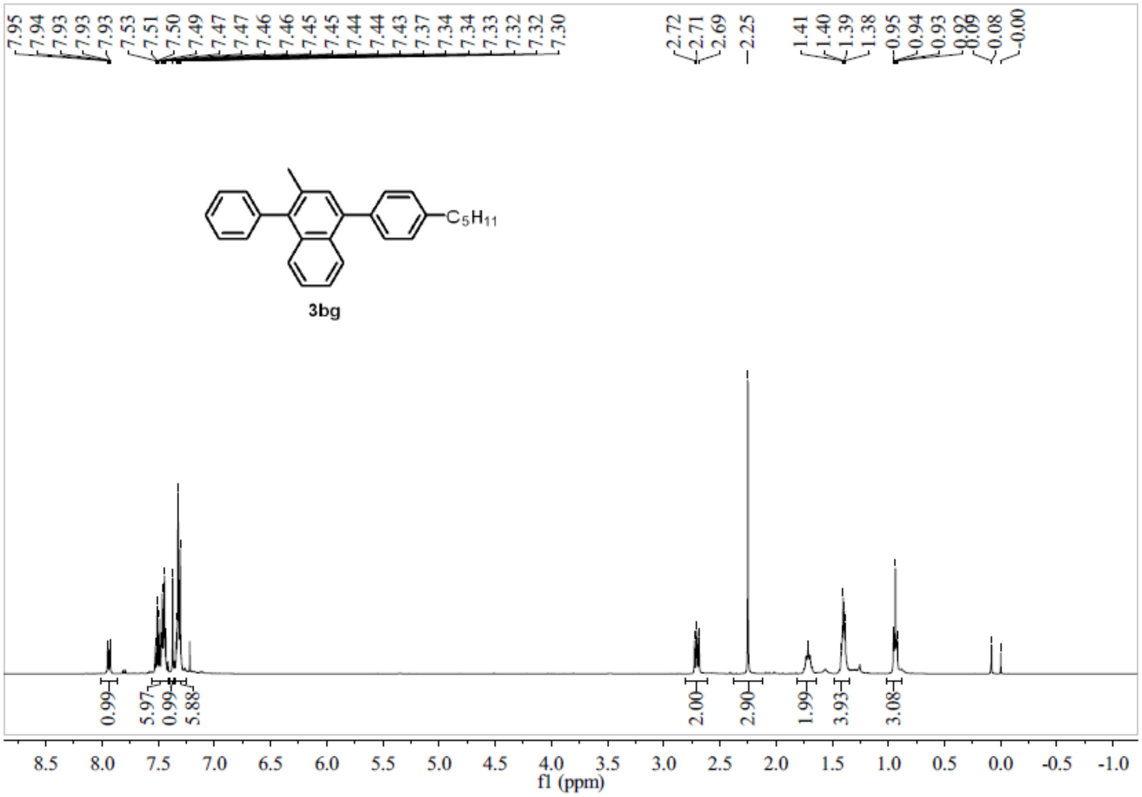

Supplementary Figure 46. ^{1}H NMR of **3bg**

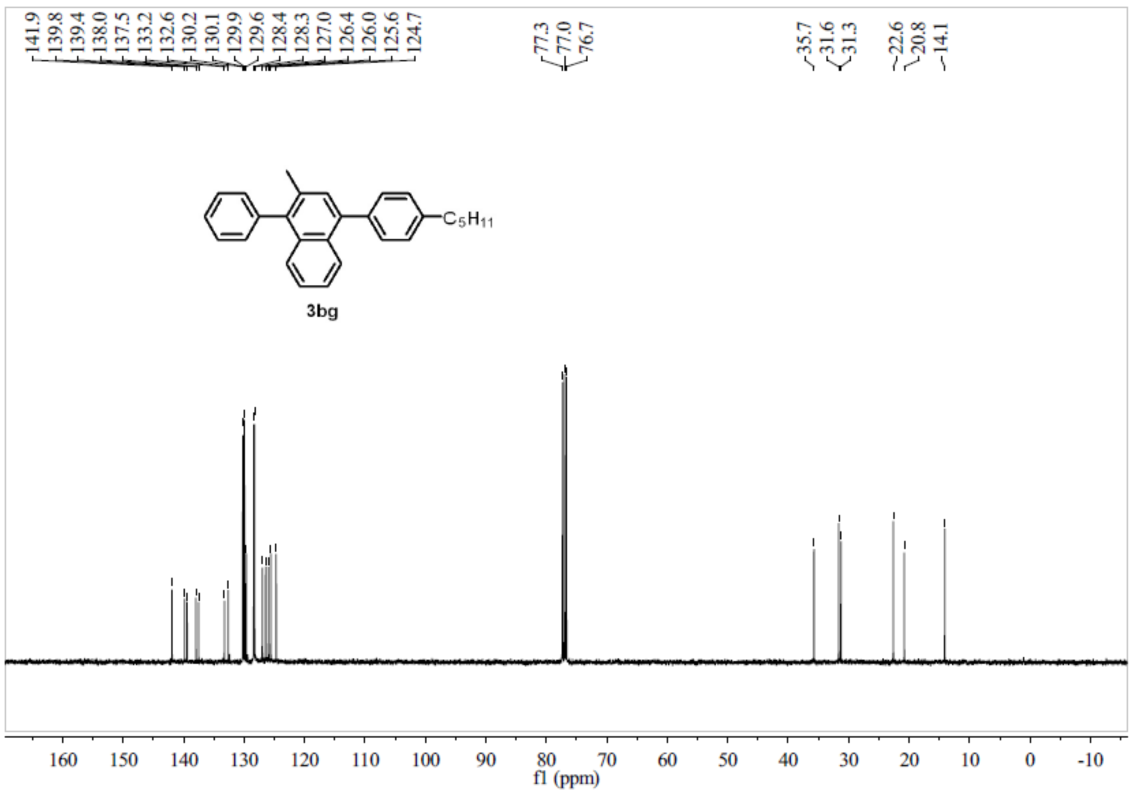

Supplementary Figure 47. ^{13}C NMR of **3bg**

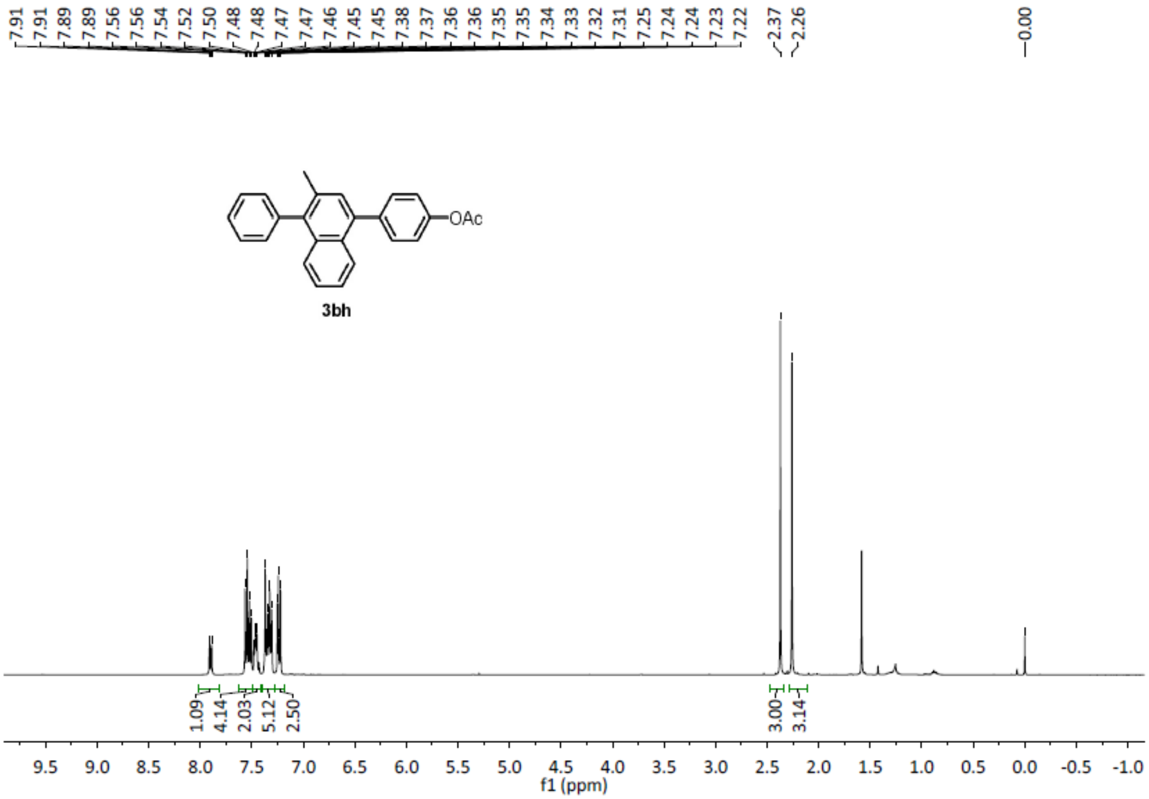

Supplementary Figure 48. ^{1}H NMR of **3bh**

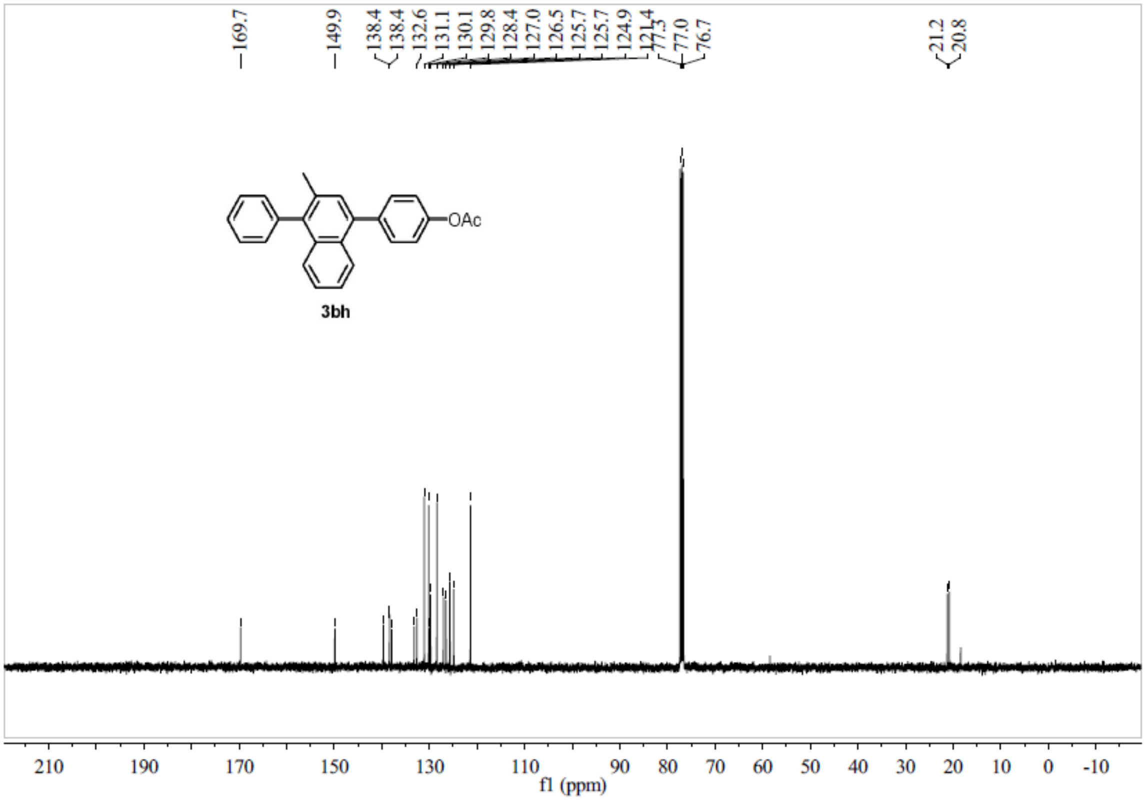

Supplementary Figure 49. ^{13}C NMR of **3bh**

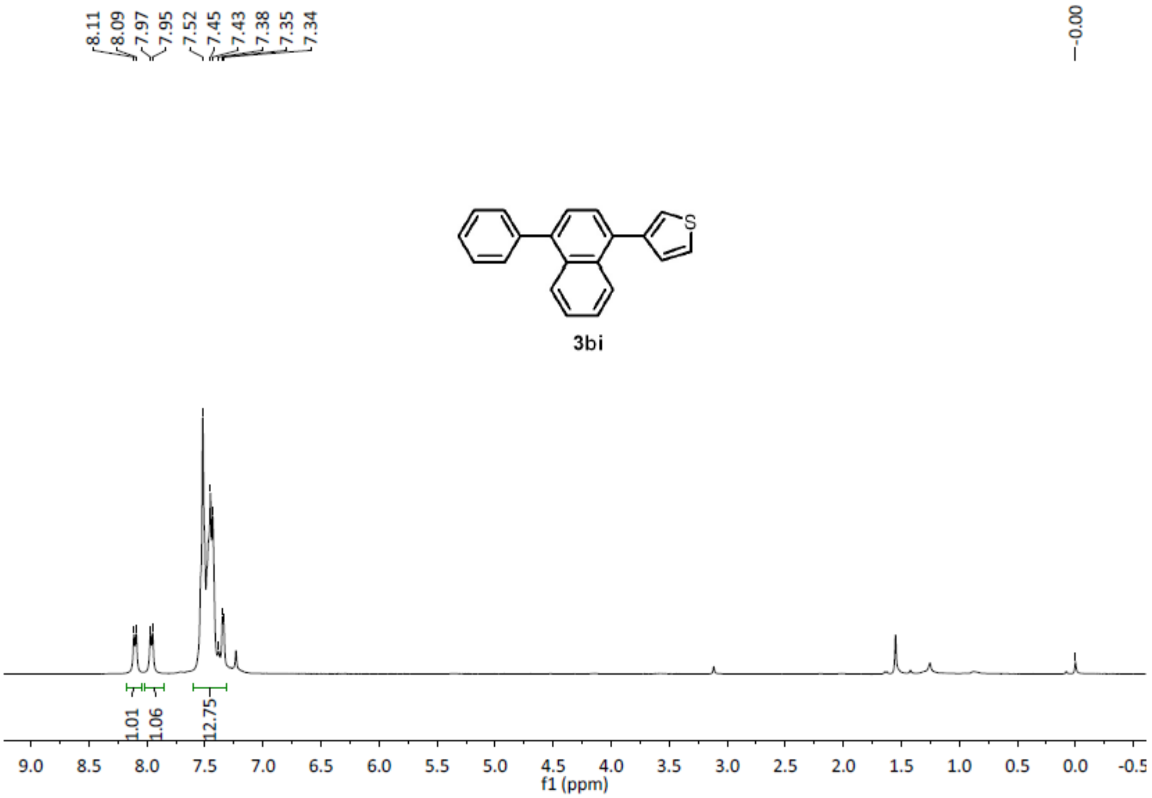

Supplementary Figure 50. ^{1}H NMR of **3bi**

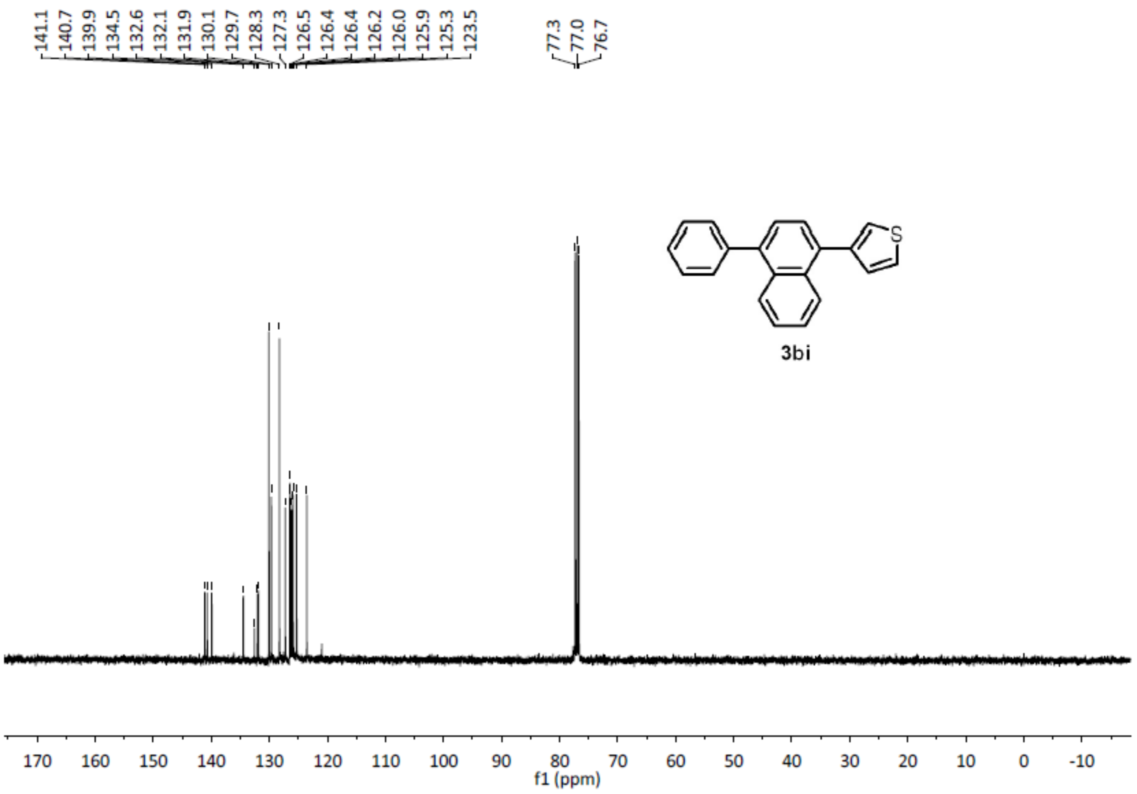

Supplementary Figure 51. ^{13}C NMR of **3bi**

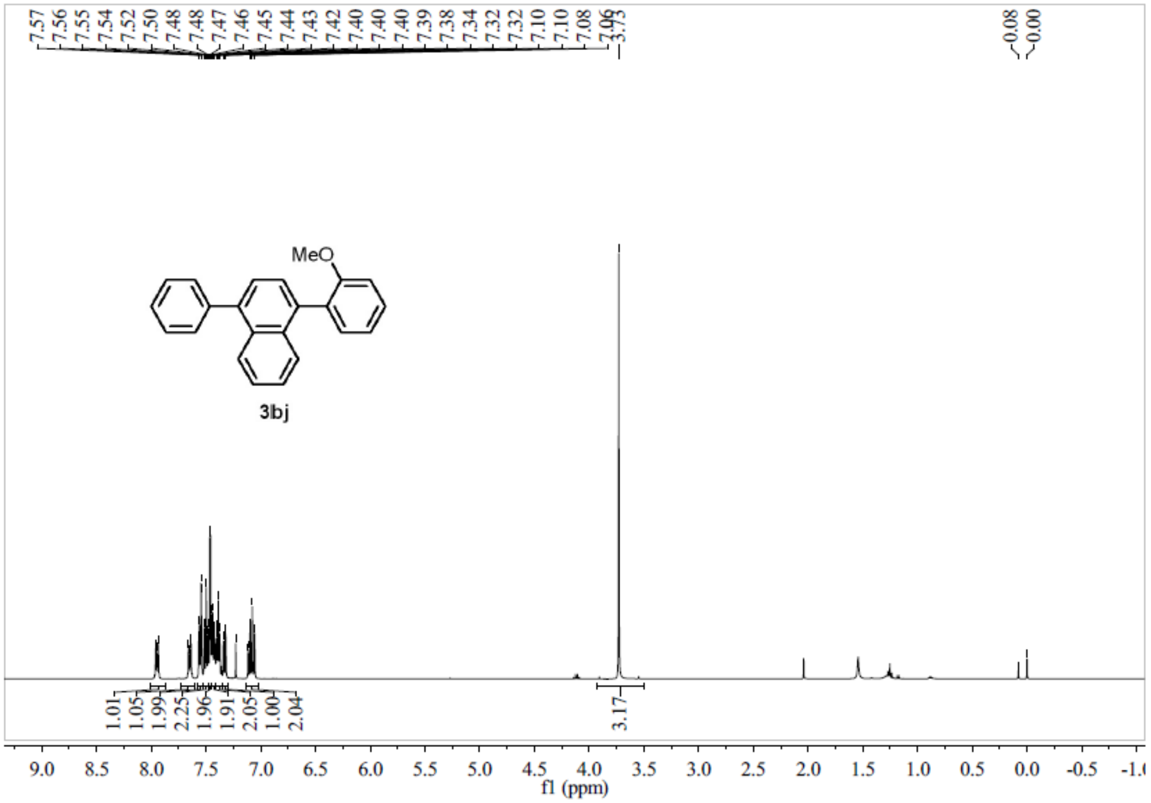

Supplementary Figure 52. ^{1}H NMR of **3bj**

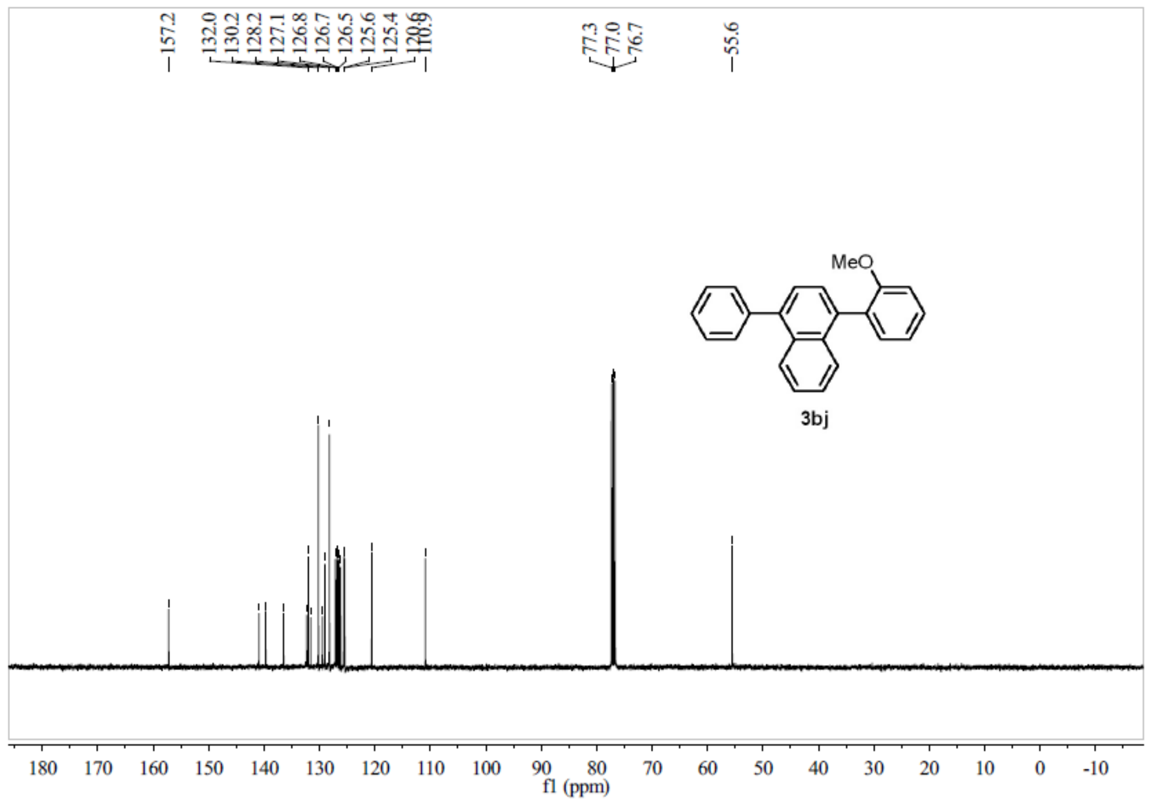

Supplementary Figure 53. ^{13}C NMR of **3bj**

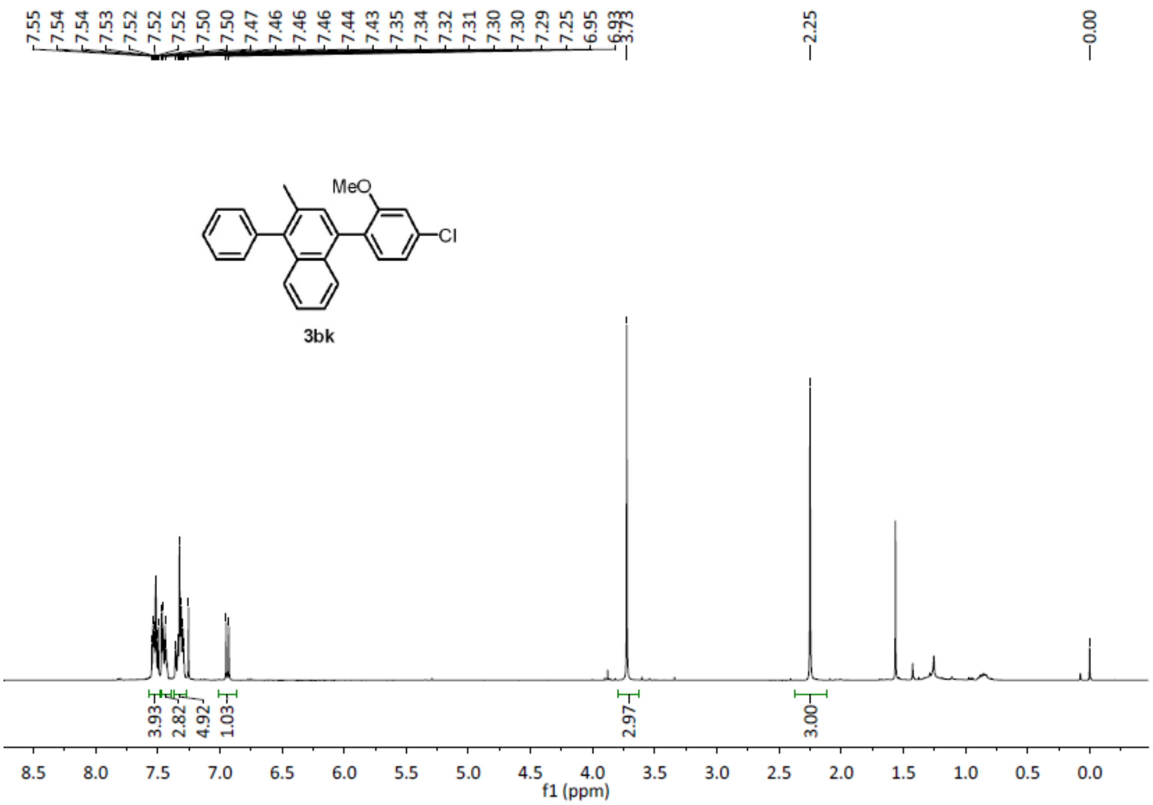

Supplementary Figure 54. ^{1}H NMR of **3bk**

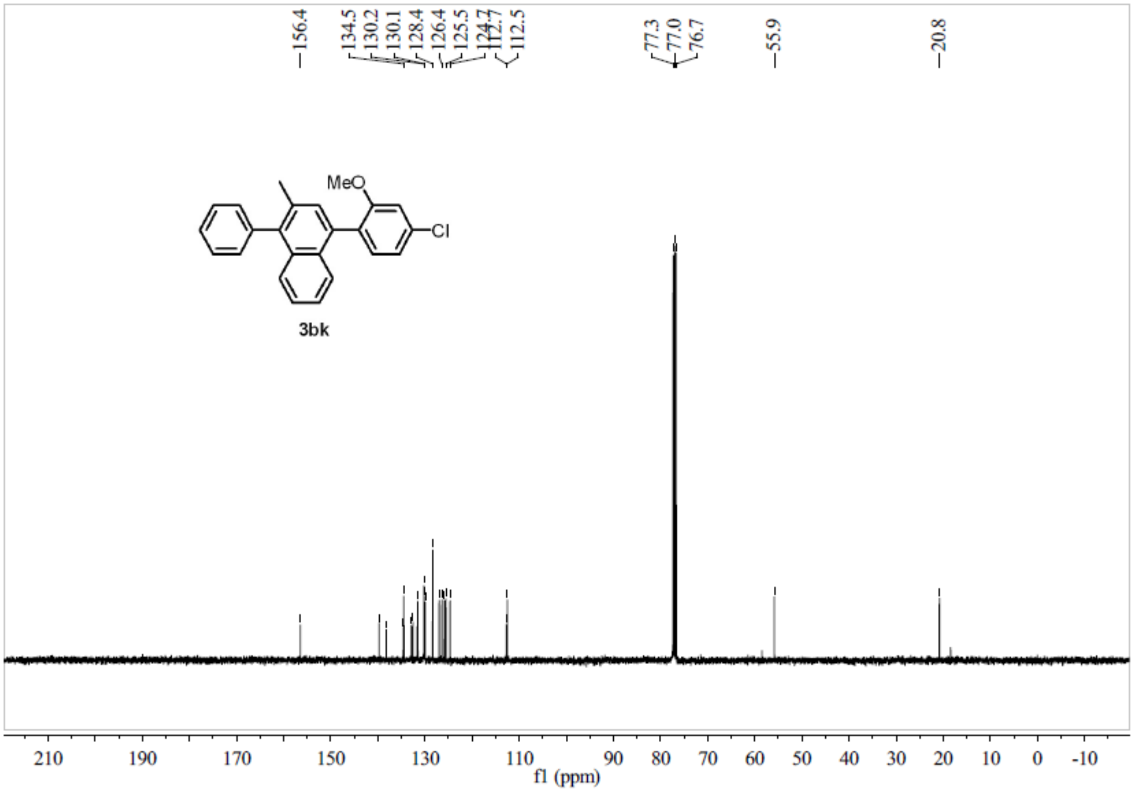

Supplementary Figure 55. ^{13}C NMR of **3bk**

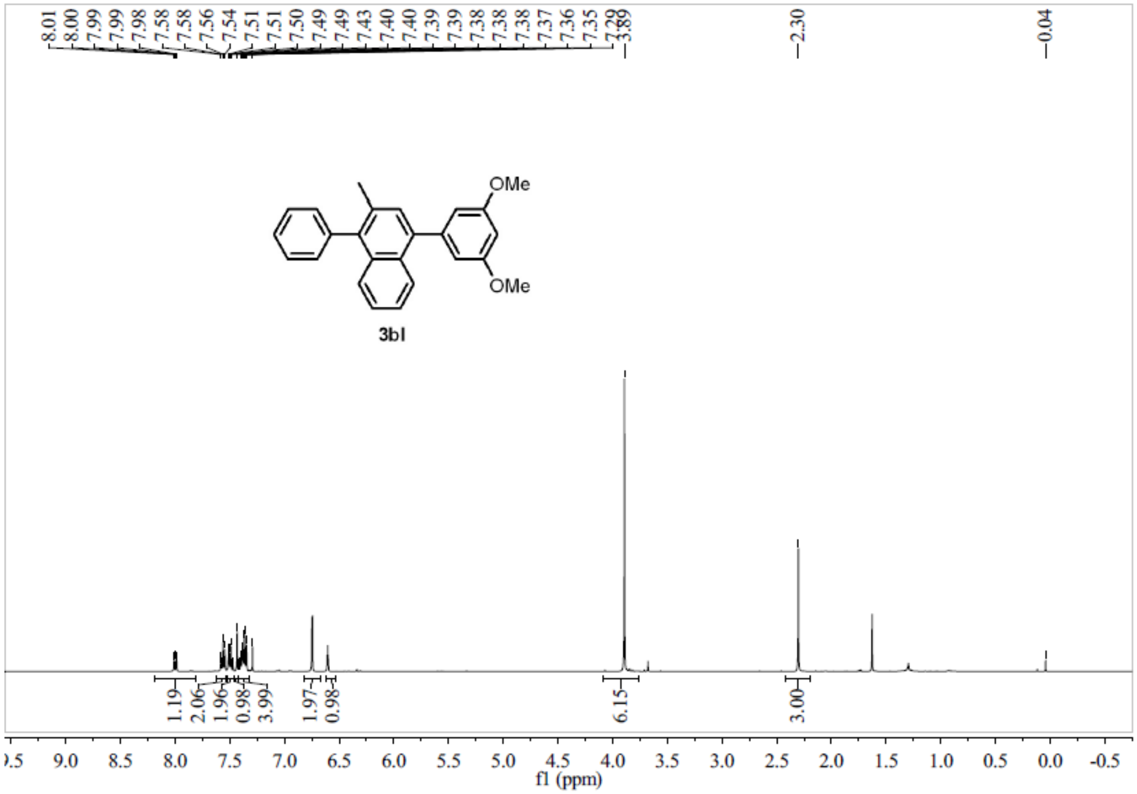

Supplementary Figure 56. ^{1}H NMR of **3bl**

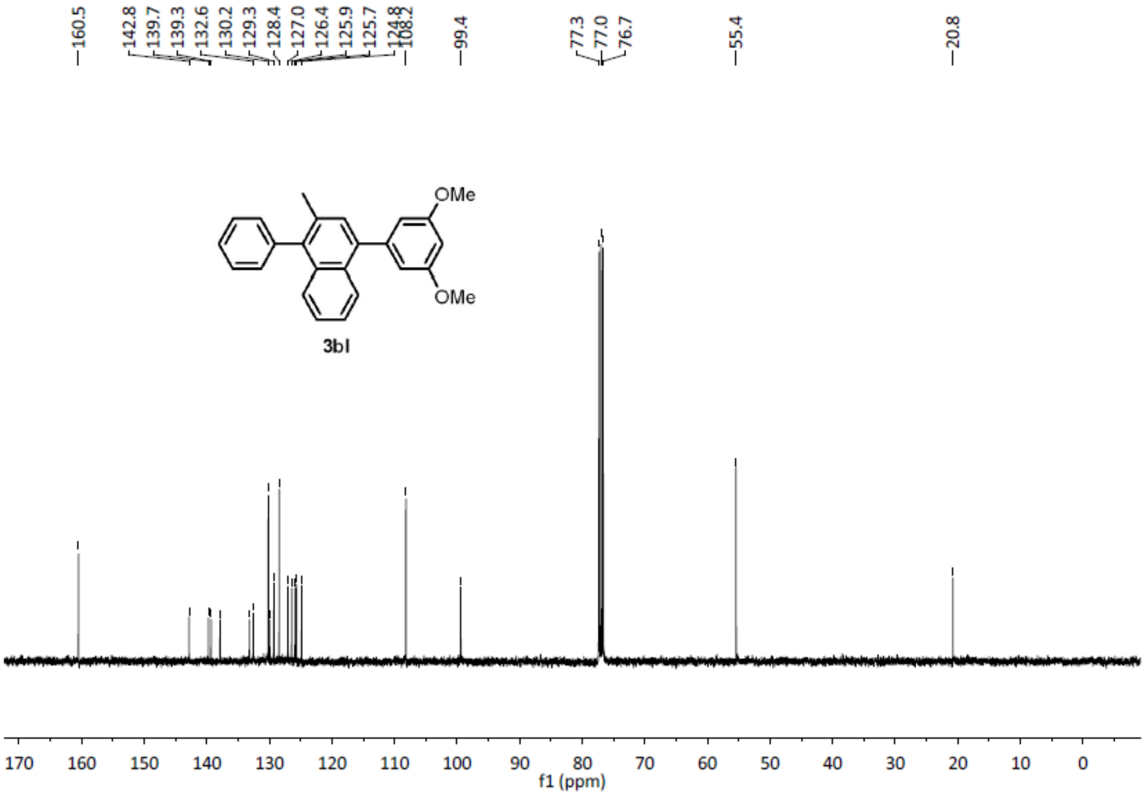

Supplementary Figure 57. ^{13}C NMR of **3bl**

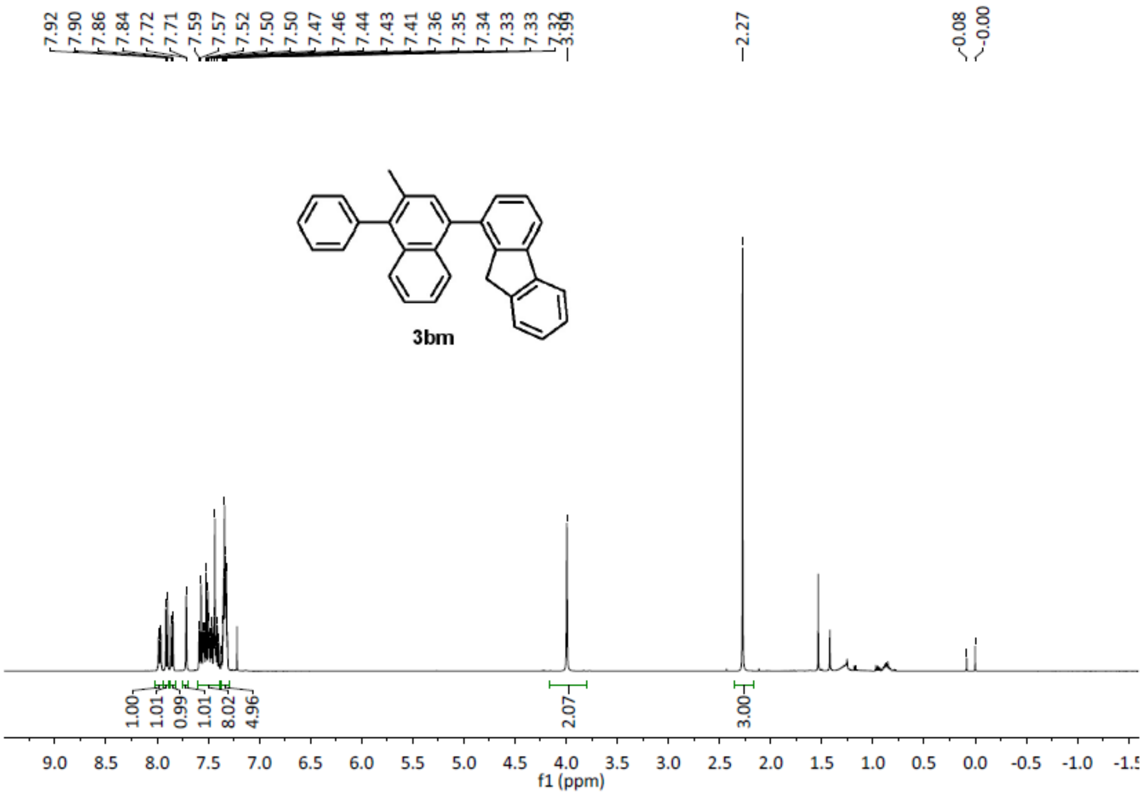

Supplementary Figure 58. ^{1}H NMR of **3bm**

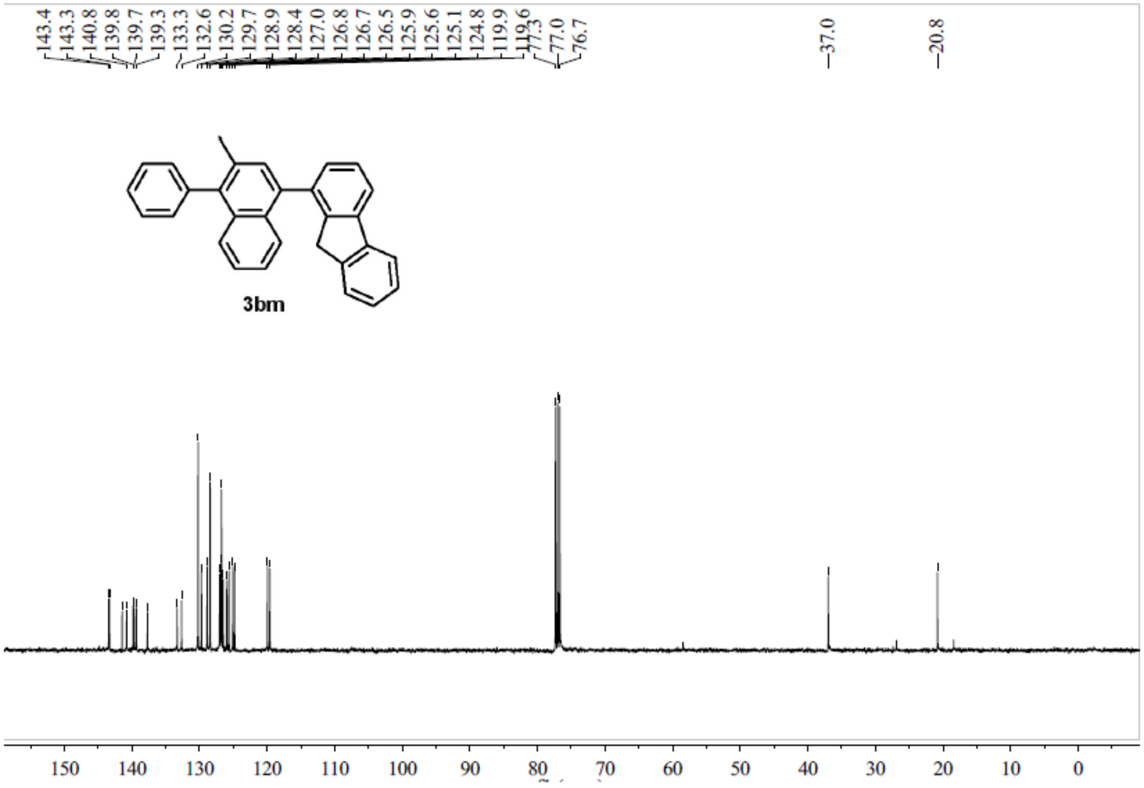

Supplementary Figure 59. ^{1}H NMR of **3bm**

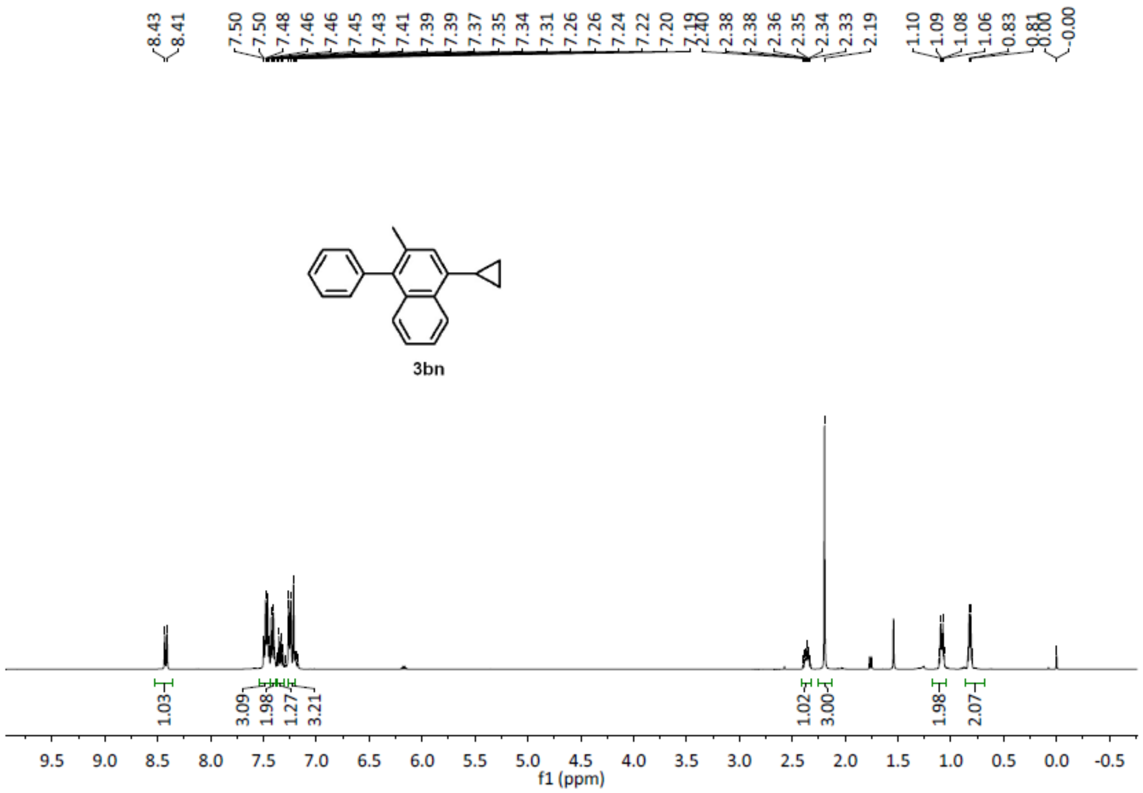

Supplementary Figure 60. ^{1}H NMR of **3bn**

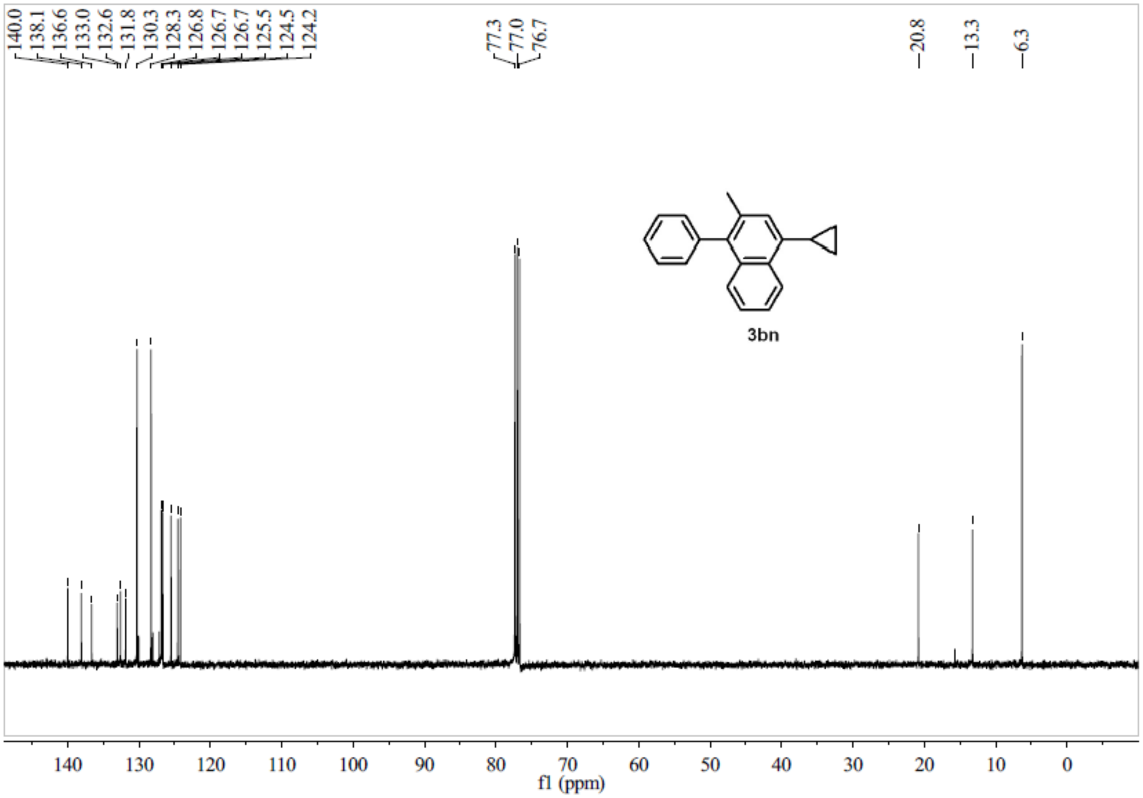

Supplementary Figure 61. ^{13}C NMR of **3bn**

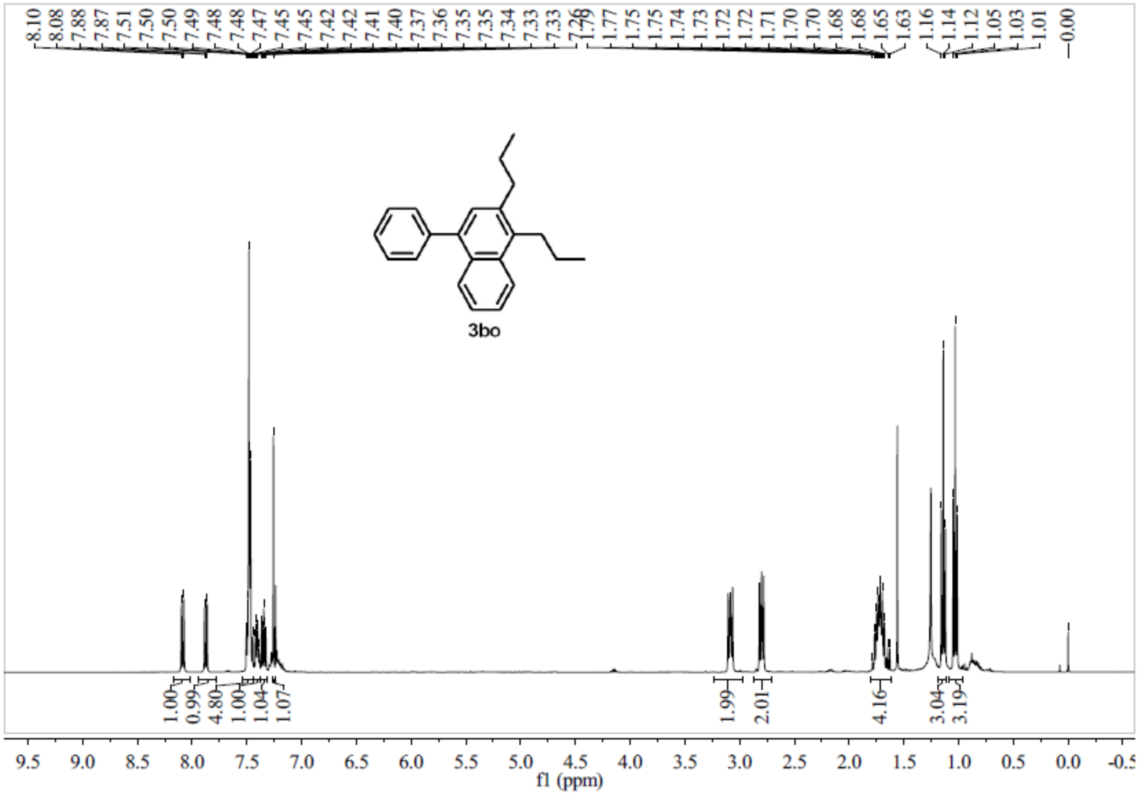

Supplementary Figure 62. ^{1}H NMR of **3bo**

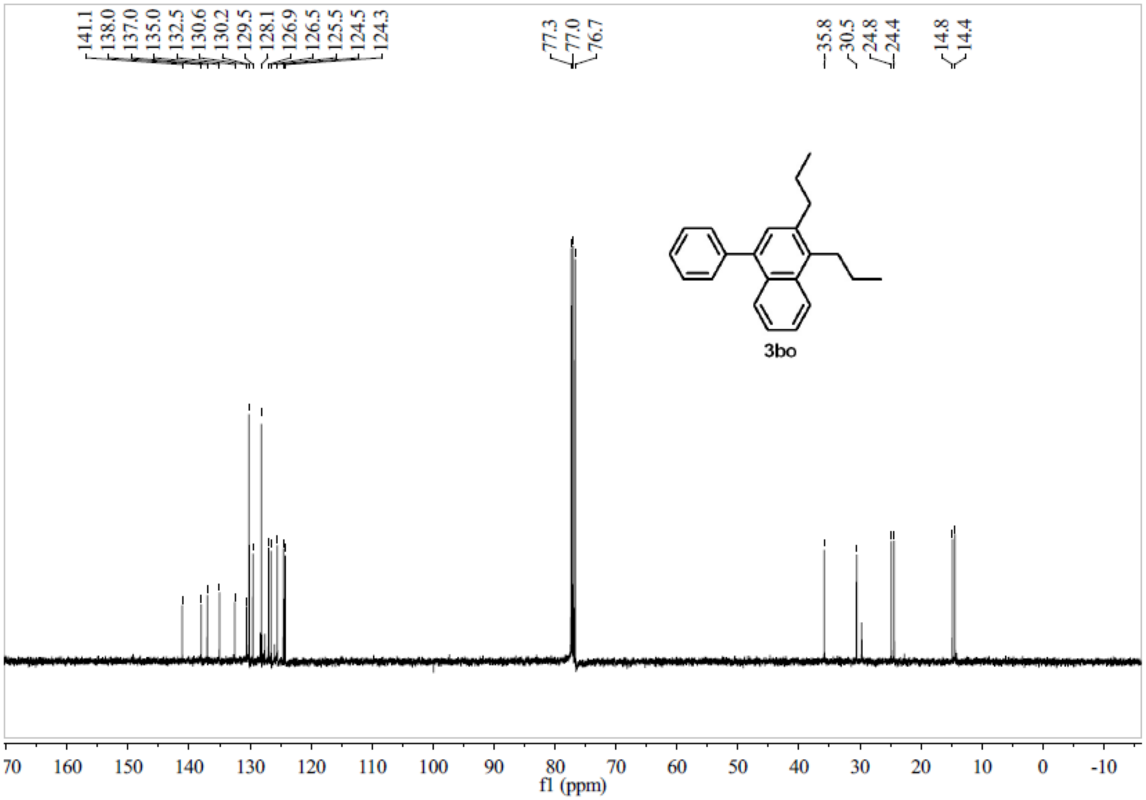

Supplementary Figure 63. ^{13}C NMR of **3bo**

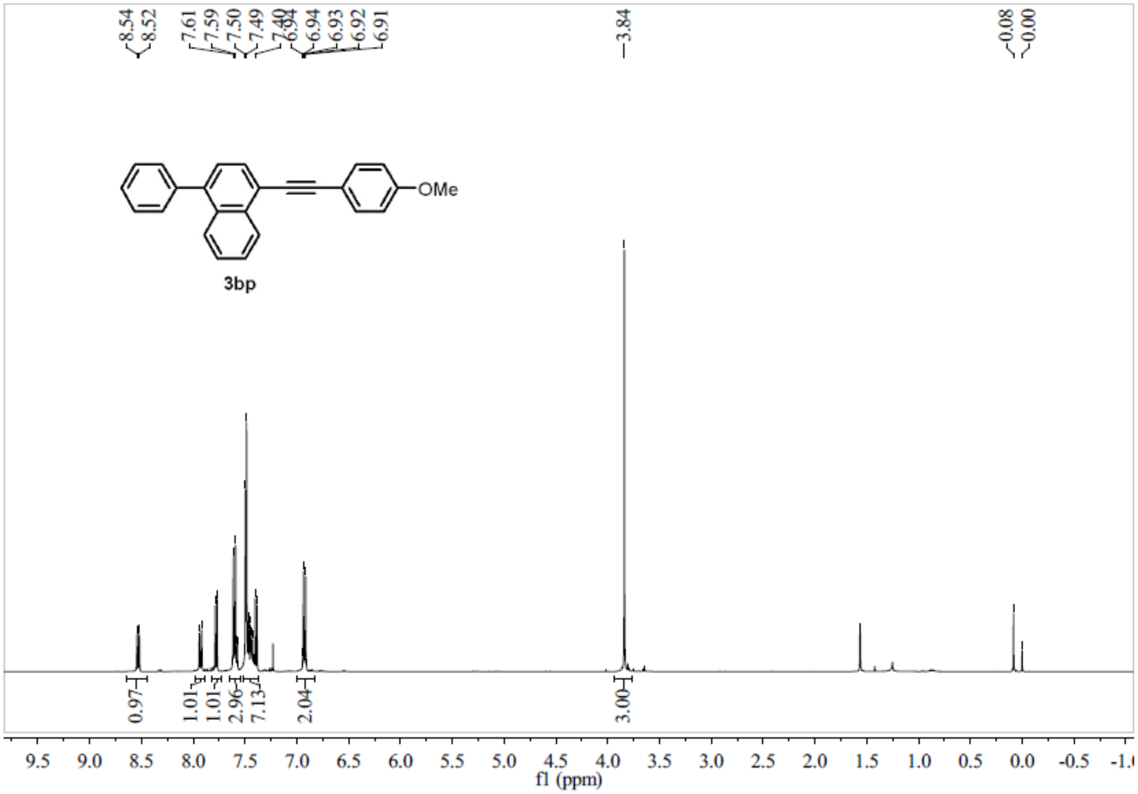

Supplementary Figure 64. ^{1}H NMR of **3bp**

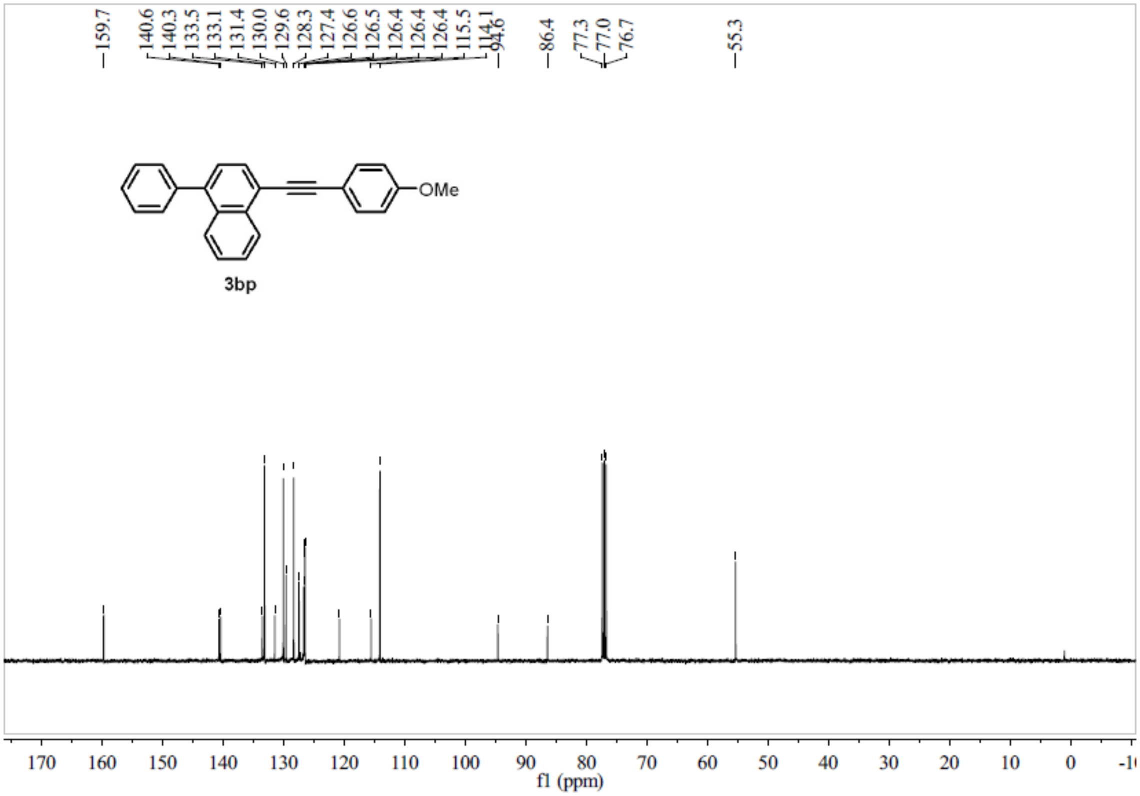

Supplementary Figure 65. ^{13}C NMR of **3bp**

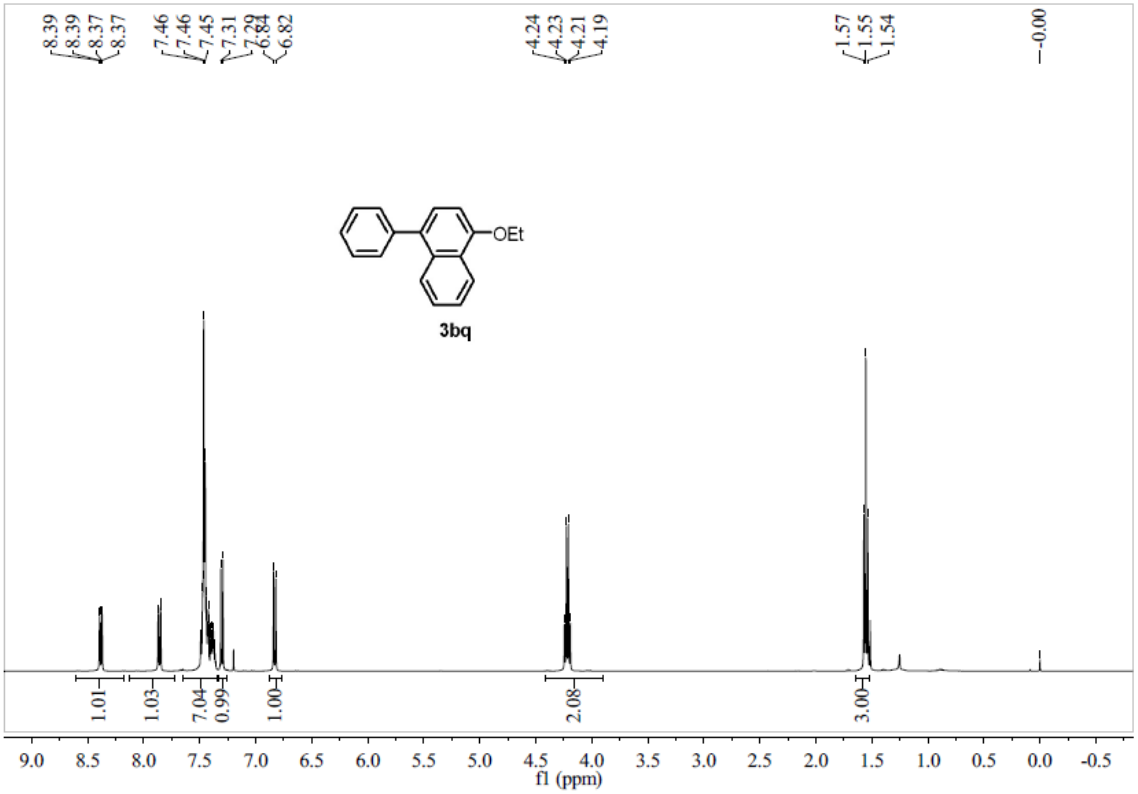

Supplementary Figure 66. ^{1}H NMR of **3bq**

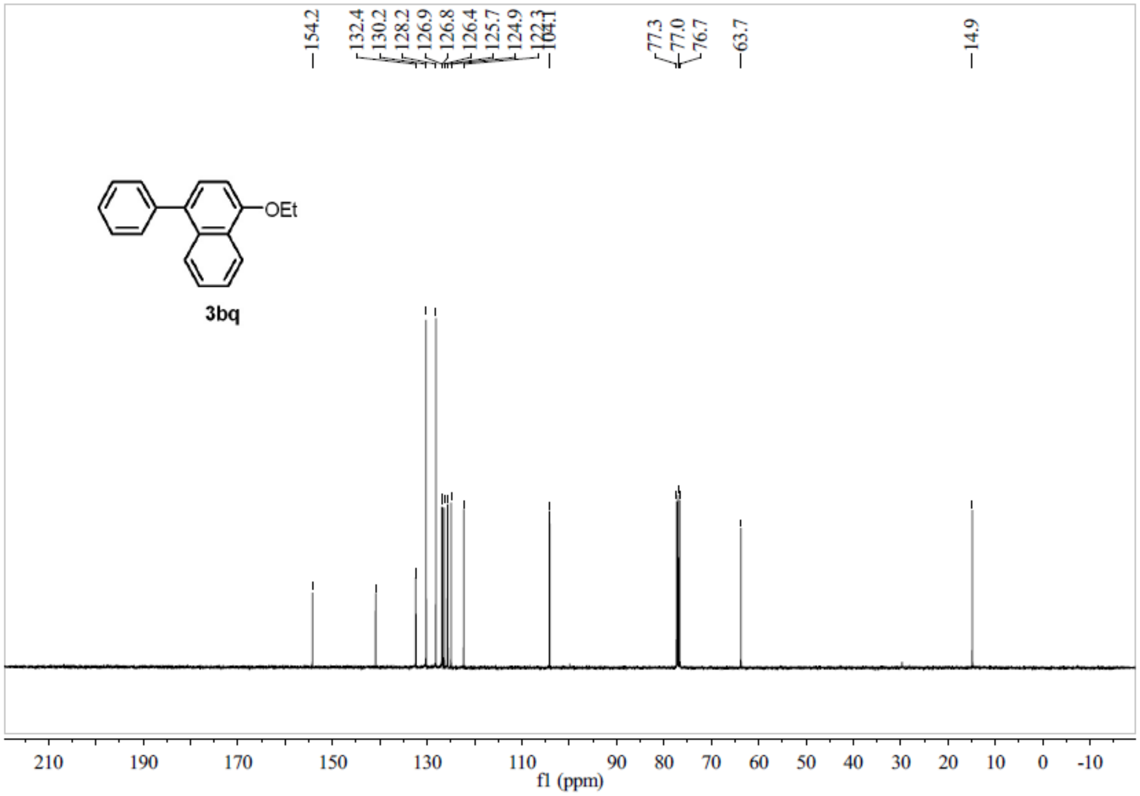

Supplementary Figure 67. ^{13}C NMR of **3bq**

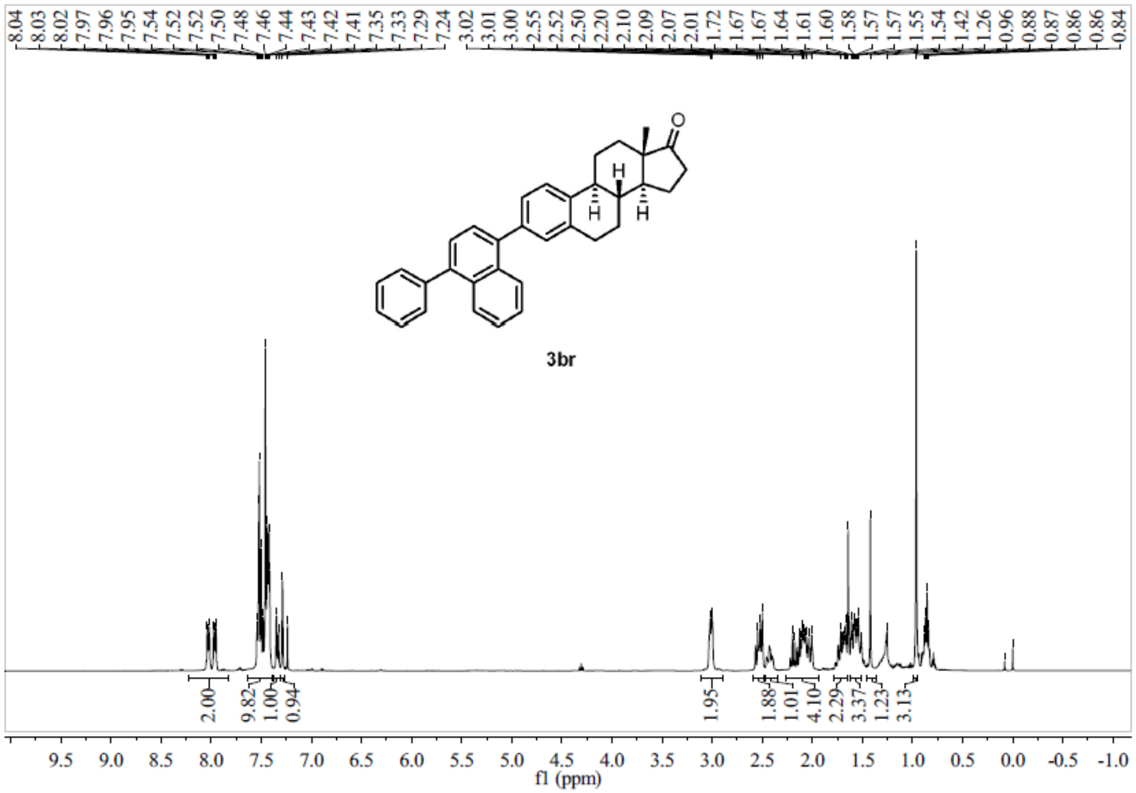

Supplementary Figure 68. ^{1}H NMR of **3br**

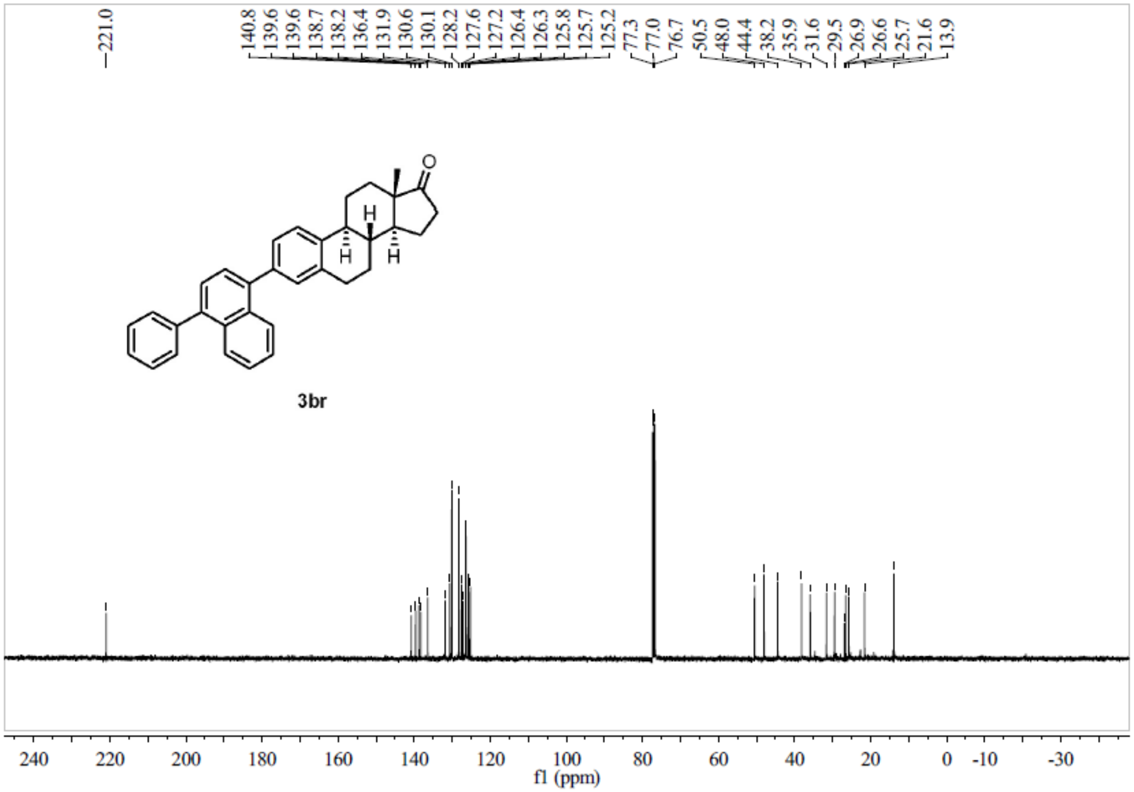

Supplementary Figure 69. ^{13}C NMR of **3br**

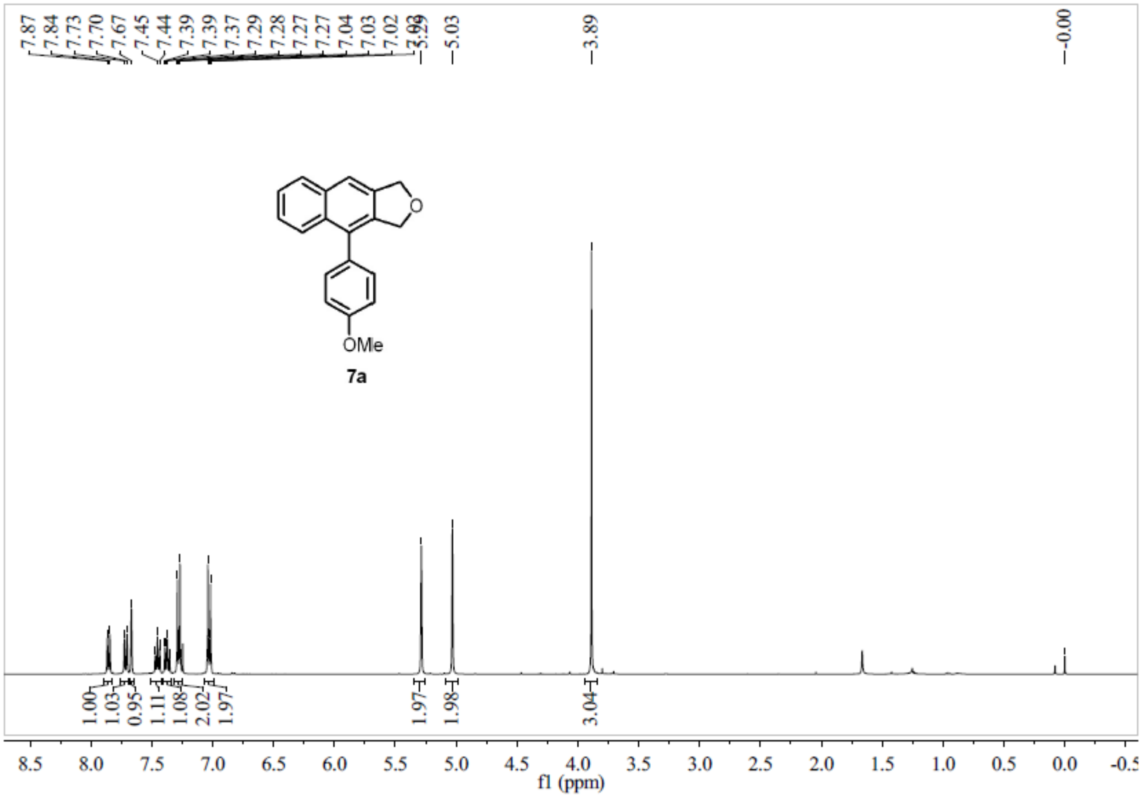

Supplementary Figure 70. ^{1}H NMR of **7a**

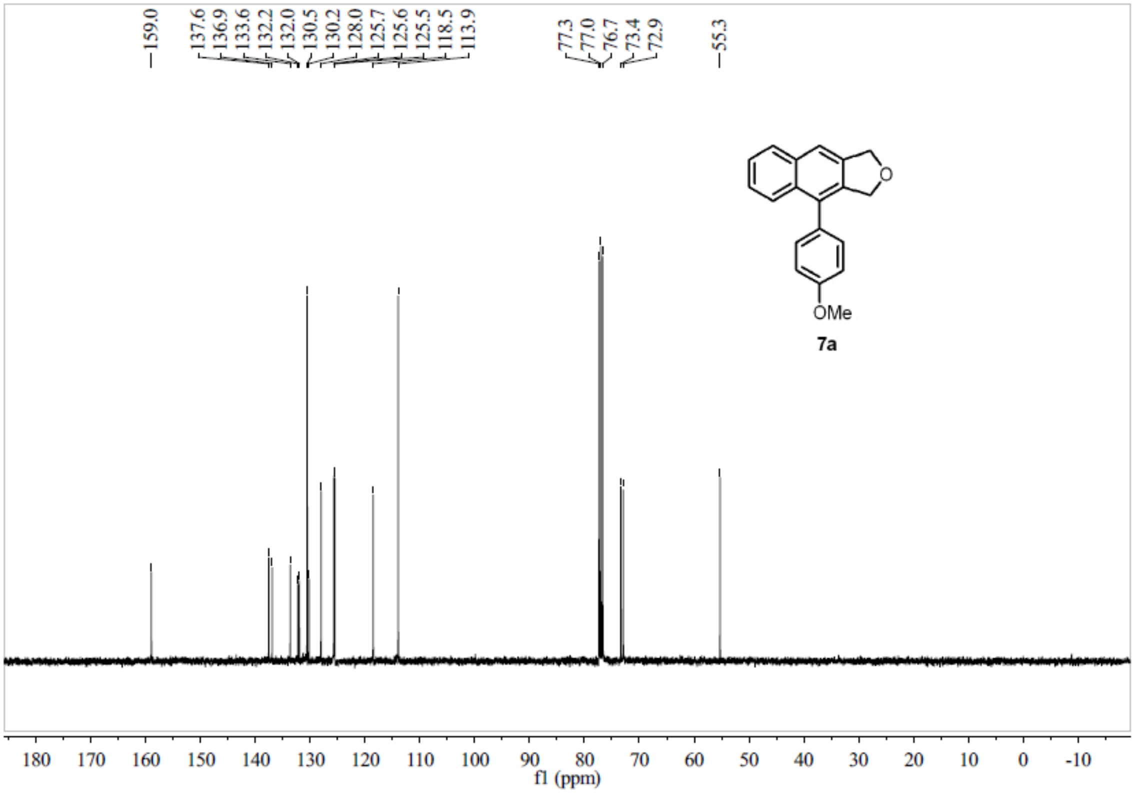

Supplementary Figure 71. ^{13}C NMR of **7a**

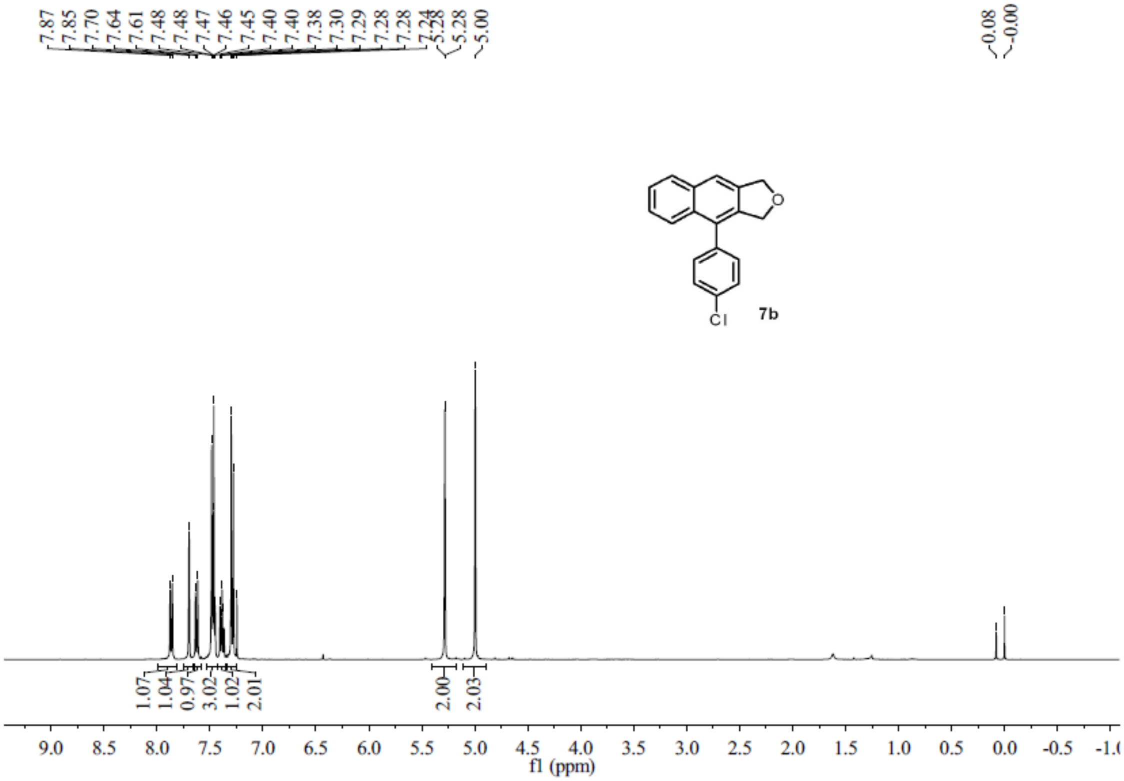

Supplementary Figure 72. ^{1}H NMR of **7b**

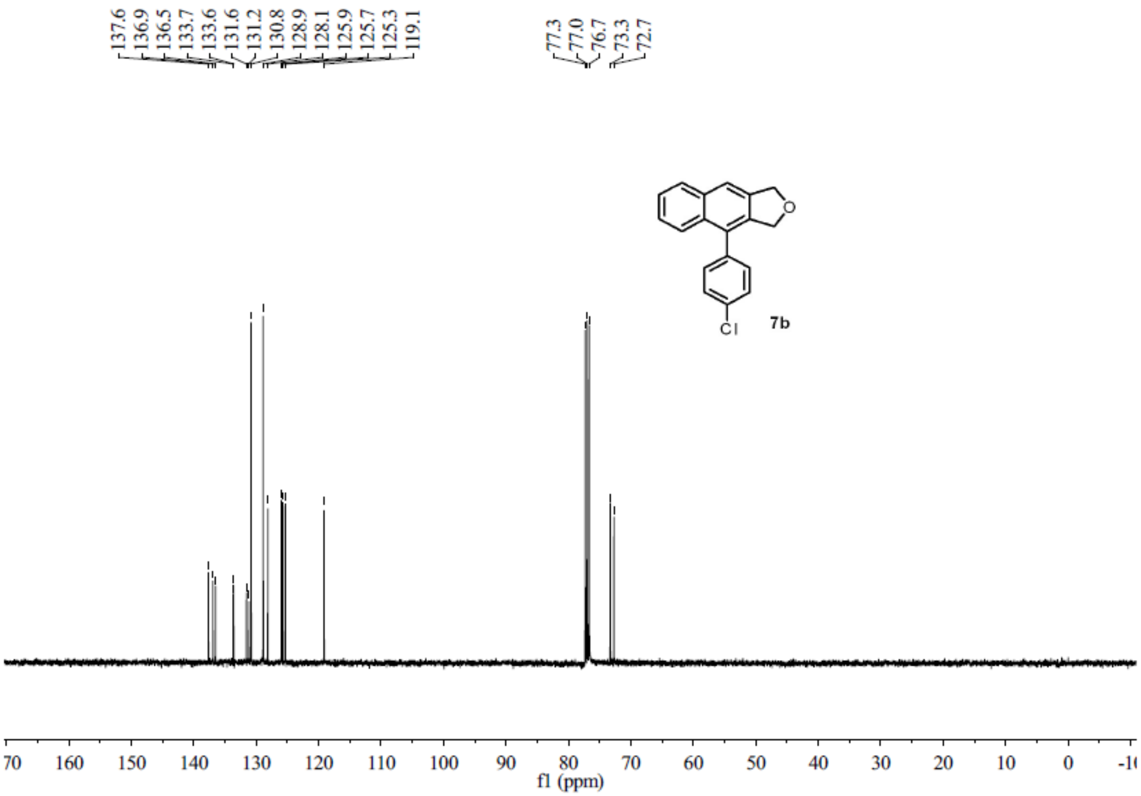

Supplementary Figure 73. ^{13}C NMR of **7b**

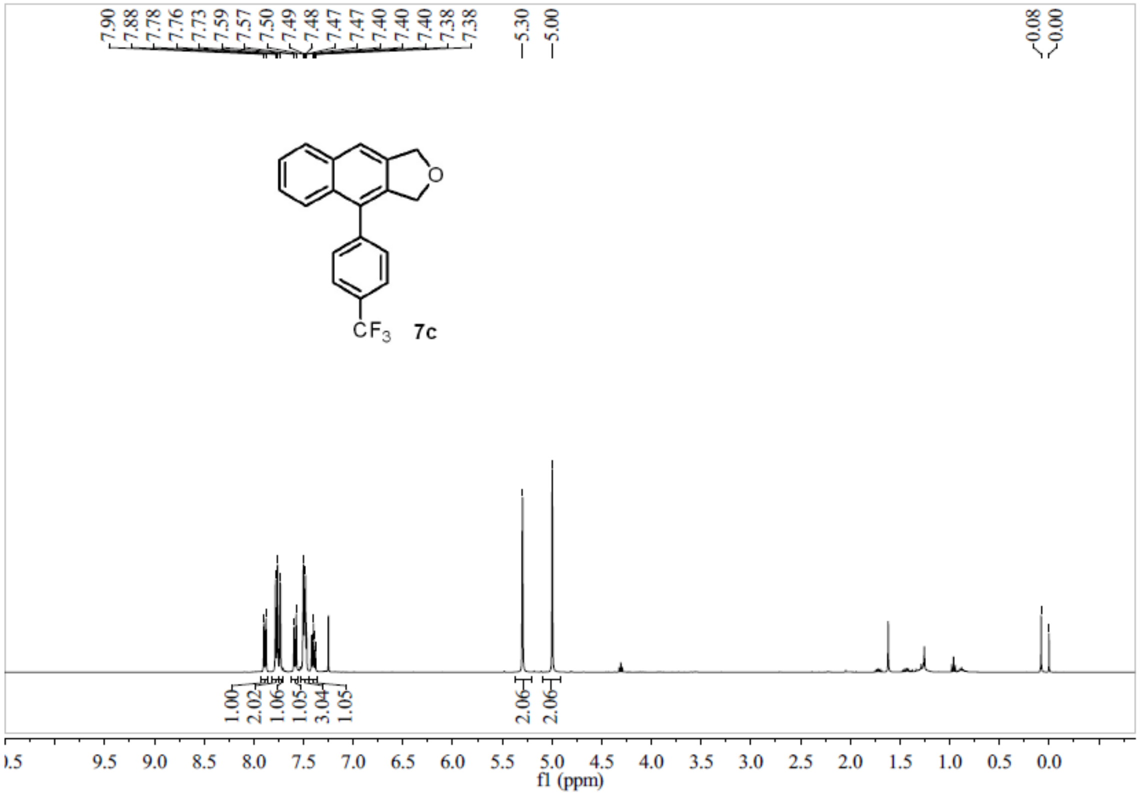

Supplementary Figure 74. ^{1}H NMR of **7c**

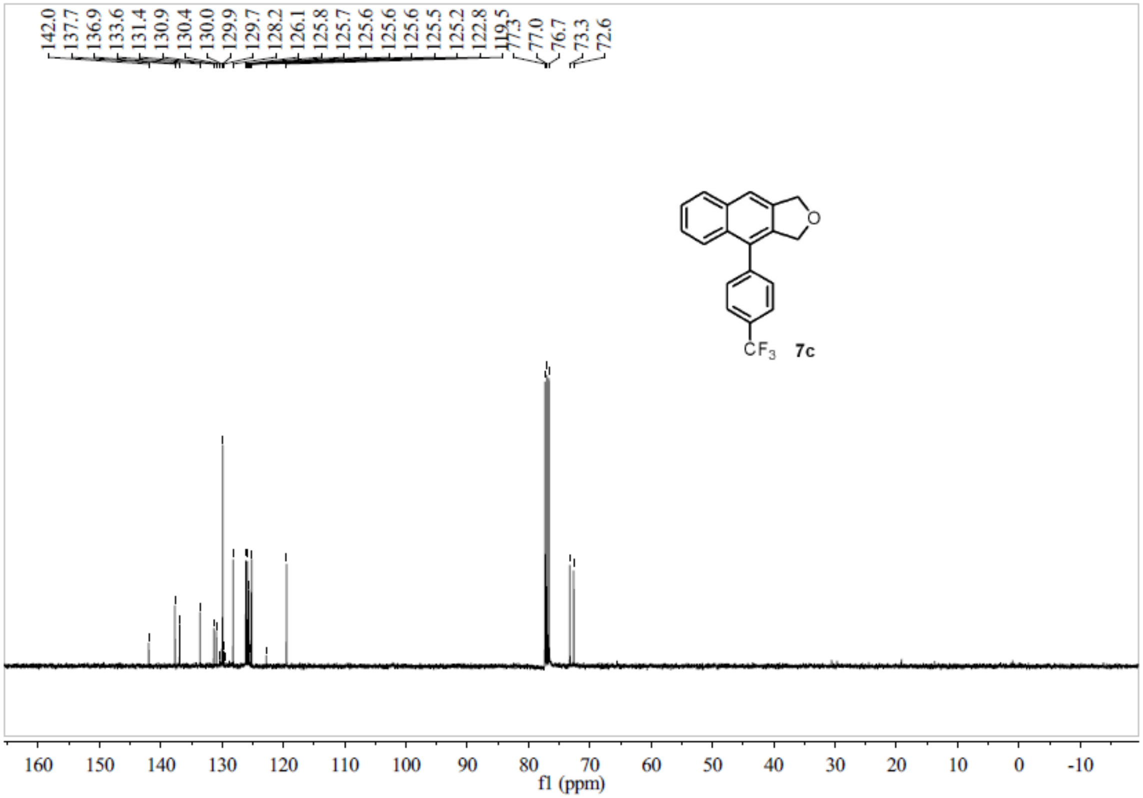

Supplementary Figure 75. ^{13}C NMR of **7c**

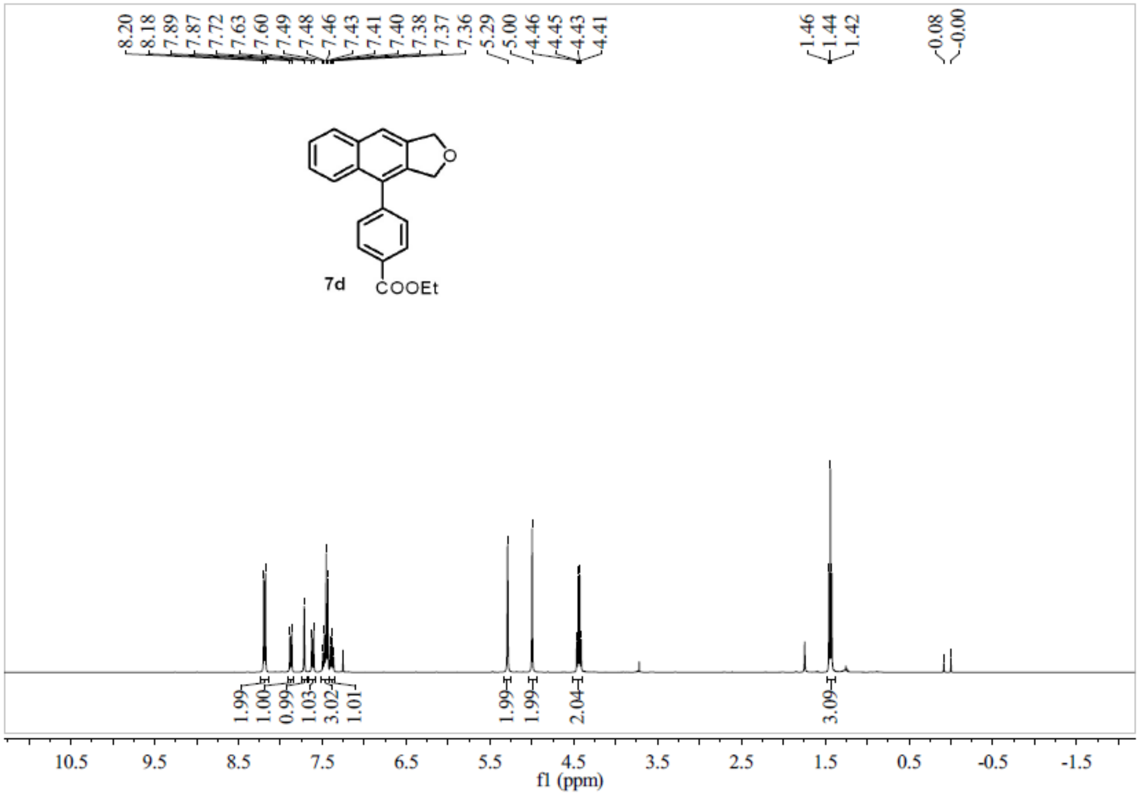

Supplementary Figure 76. ^{1}H NMR of **7d**

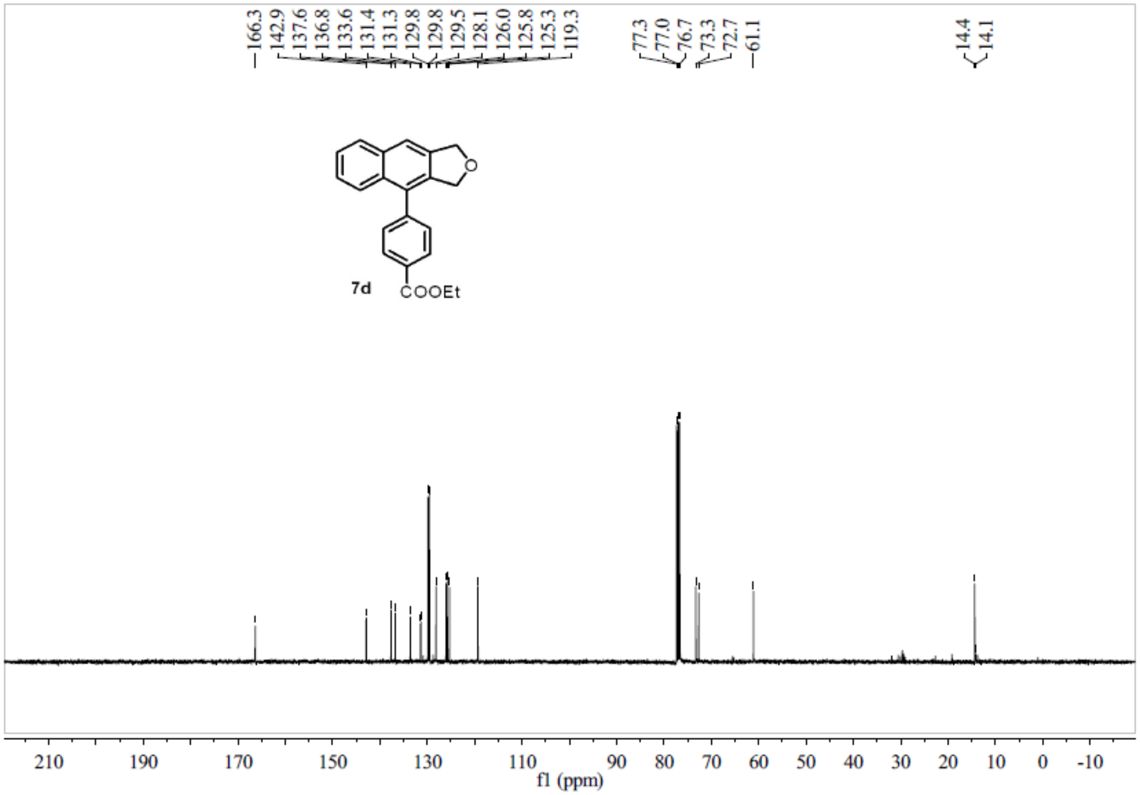

Supplementary Figure 77. ^{13}C NMR of **7d**

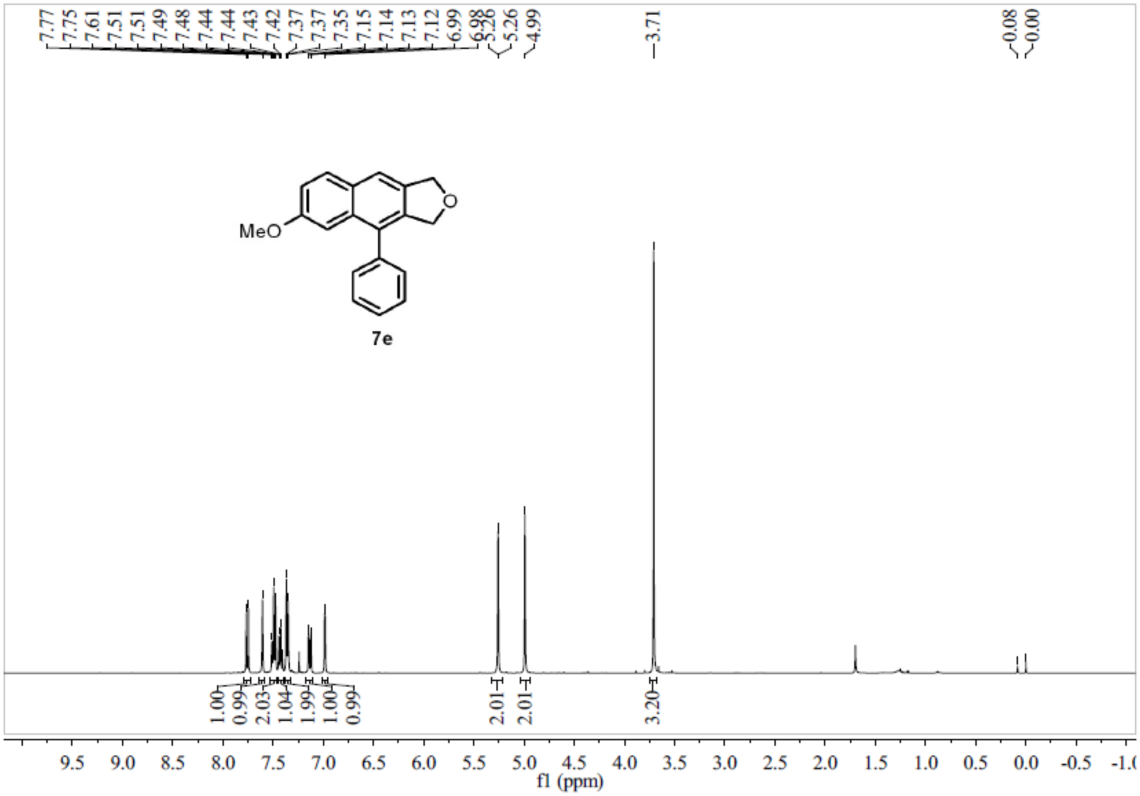

Supplementary Figure 78. ^{1}H NMR of **7e**

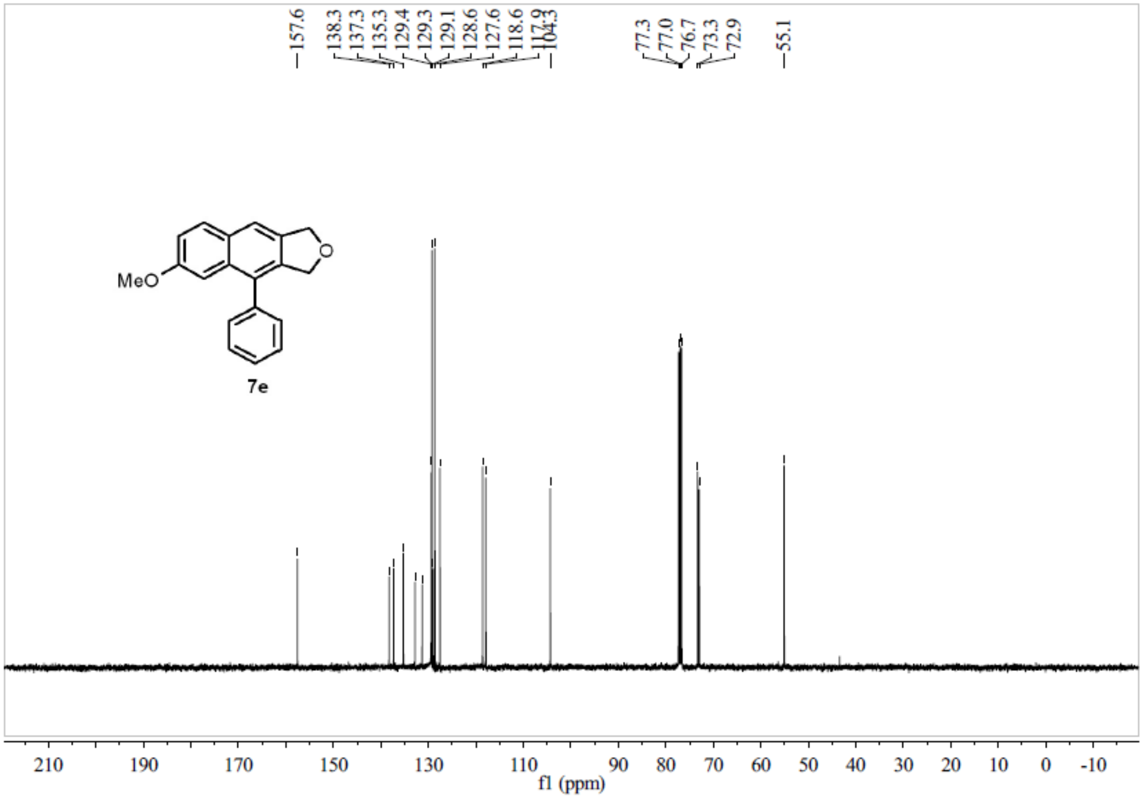

Supplementary Figure 79. ^{13}C NMR of **7e**

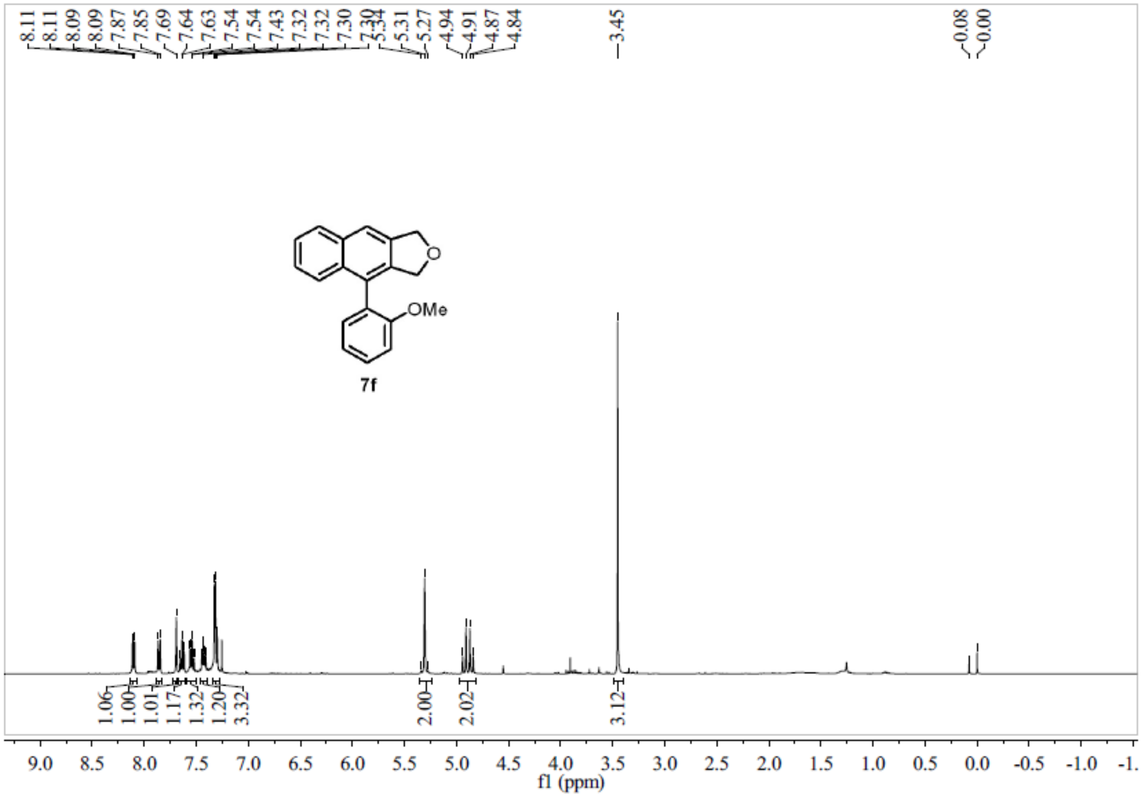

Supplementary Figure 80. ^{1}H NMR of **7f**

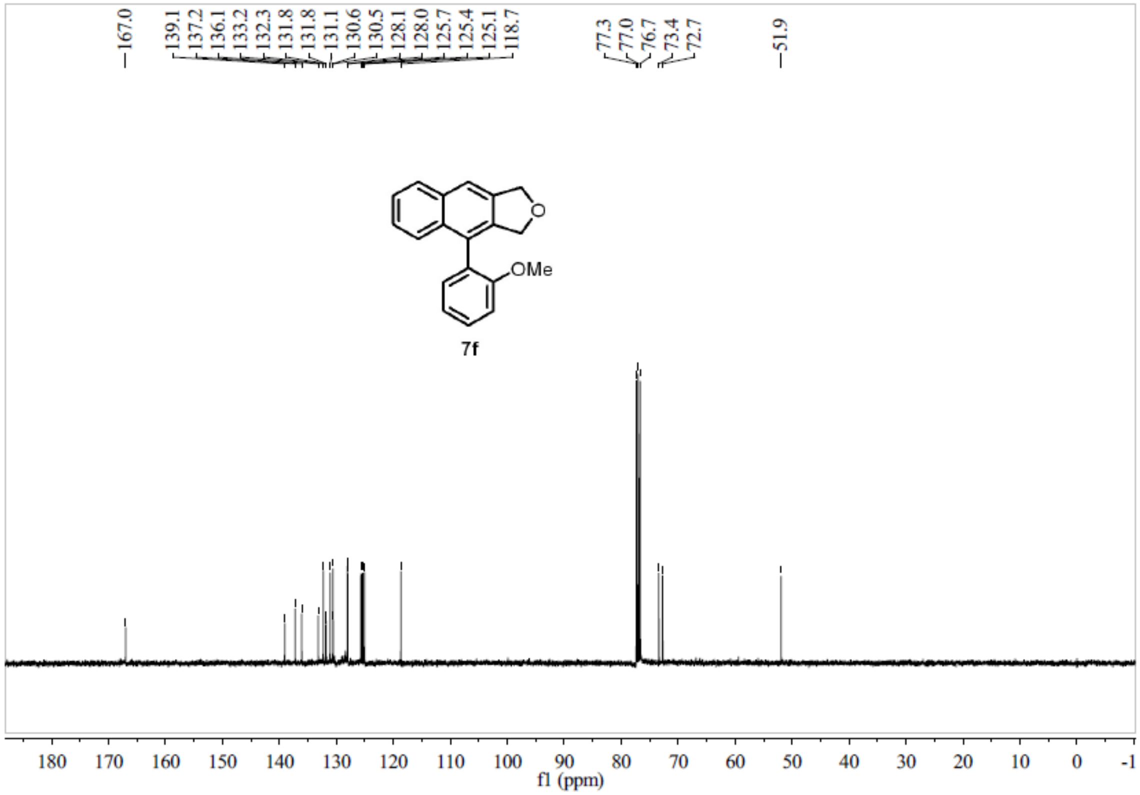

Supplementary Figure 81. ^{13}C NMR of **7f**

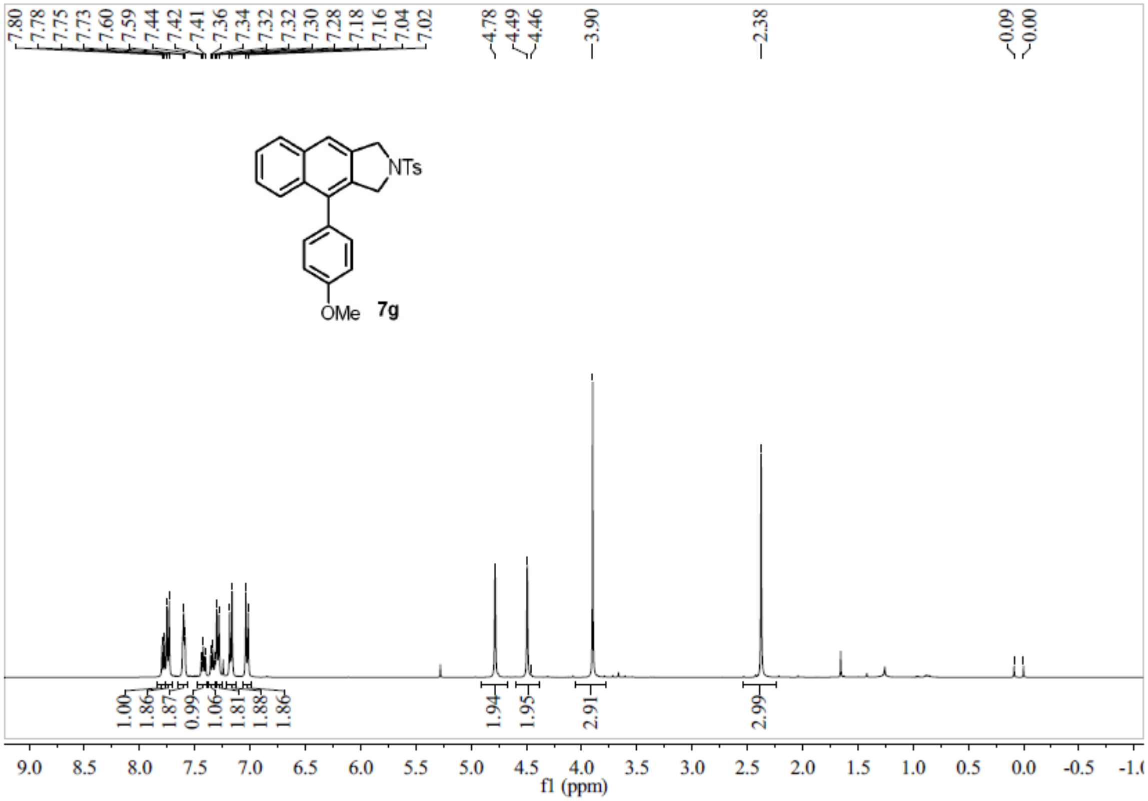

Supplementary Figure 82. ^{1}H NMR of **7g**

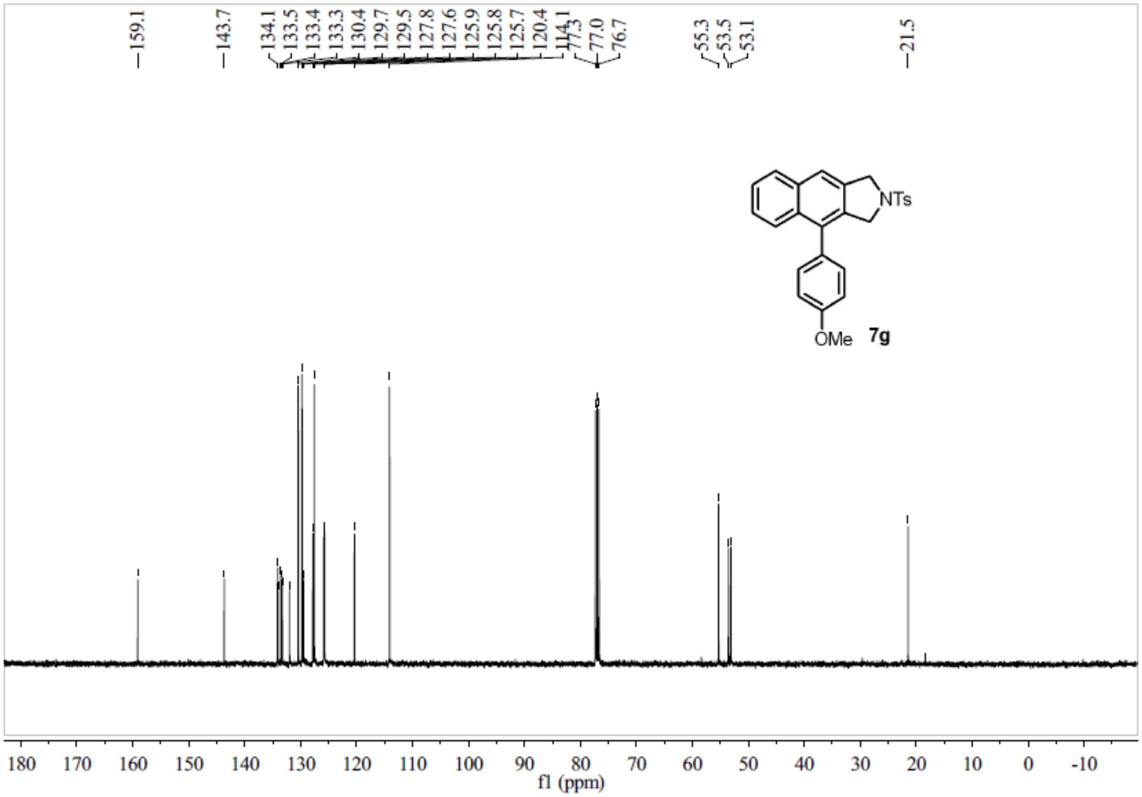

Supplementary Figure 83. ^{13}C NMR of **7g**

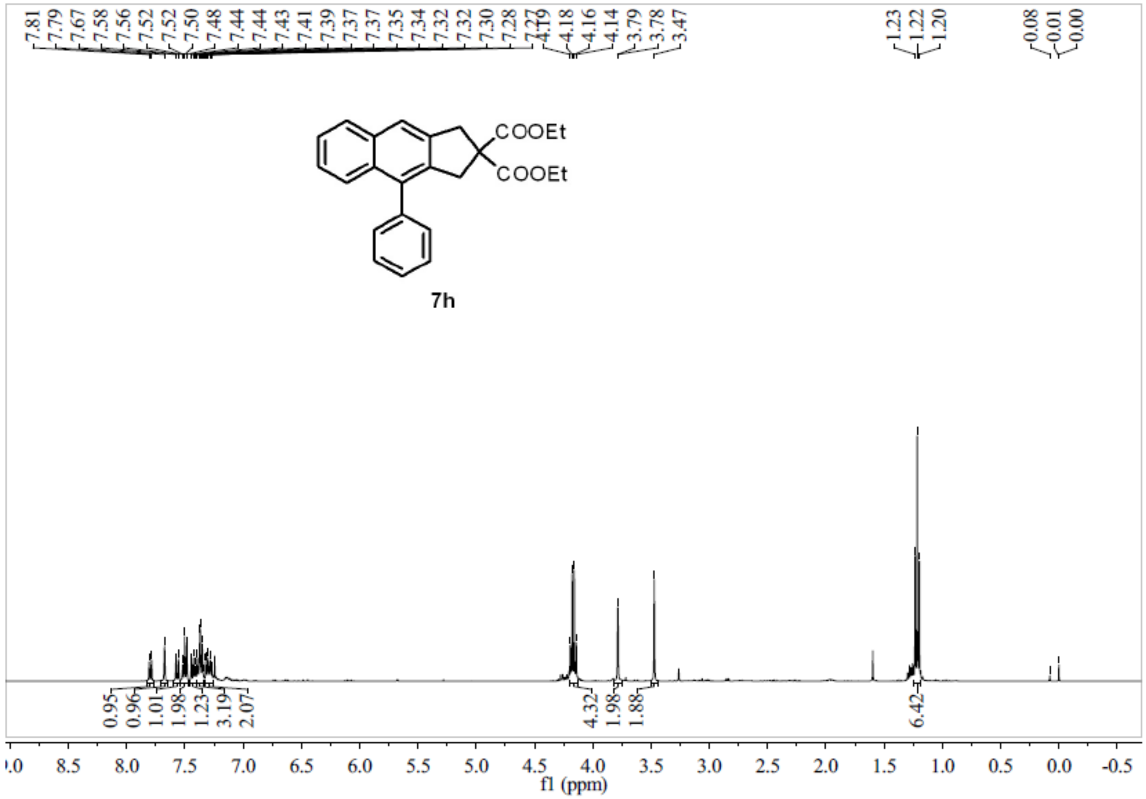

Supplementary Figure 84. ^{1}H NMR of **7h**

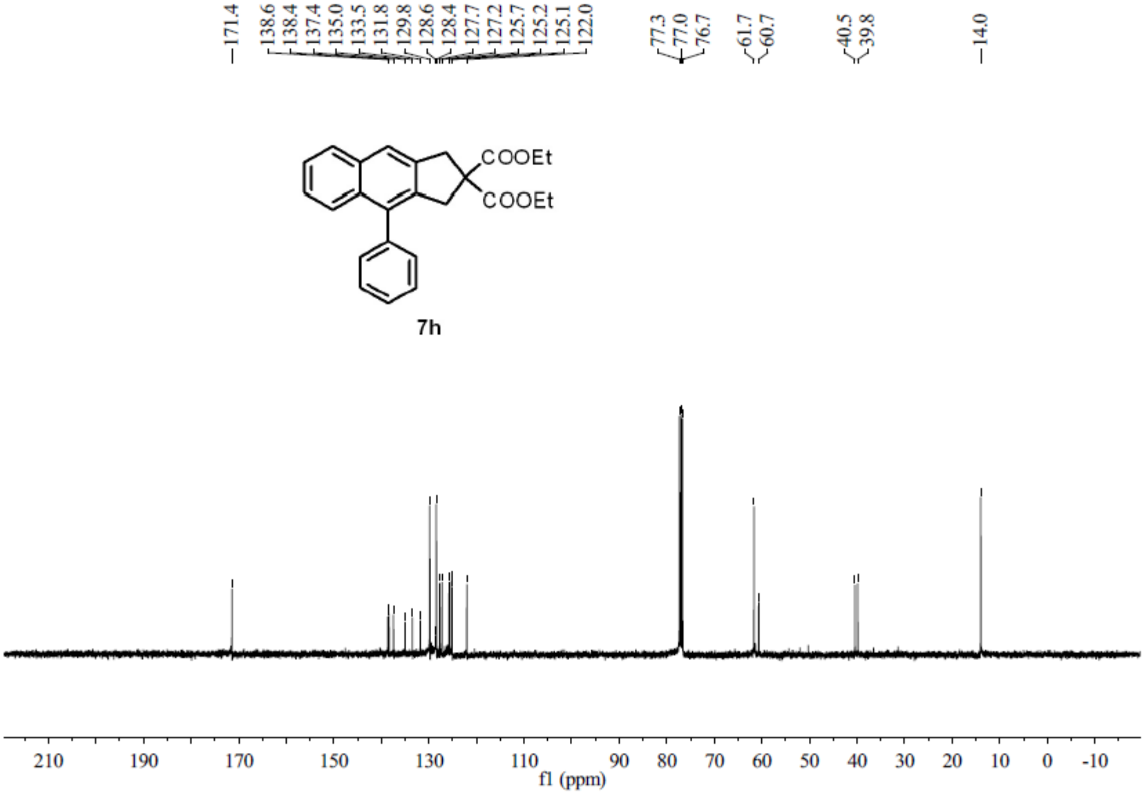

Supplementary Figure 85. ^{13}C NMR of **7h**

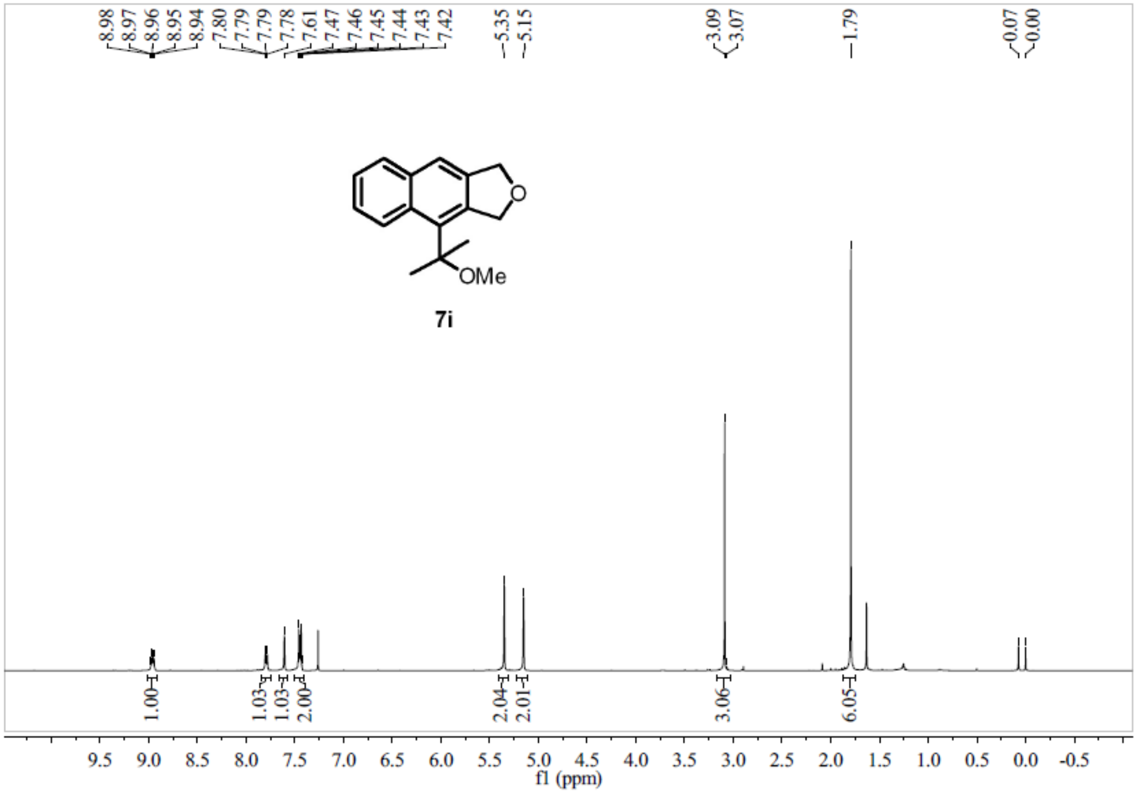

Supplementary Figure 86. ^{1}H NMR of **7i**

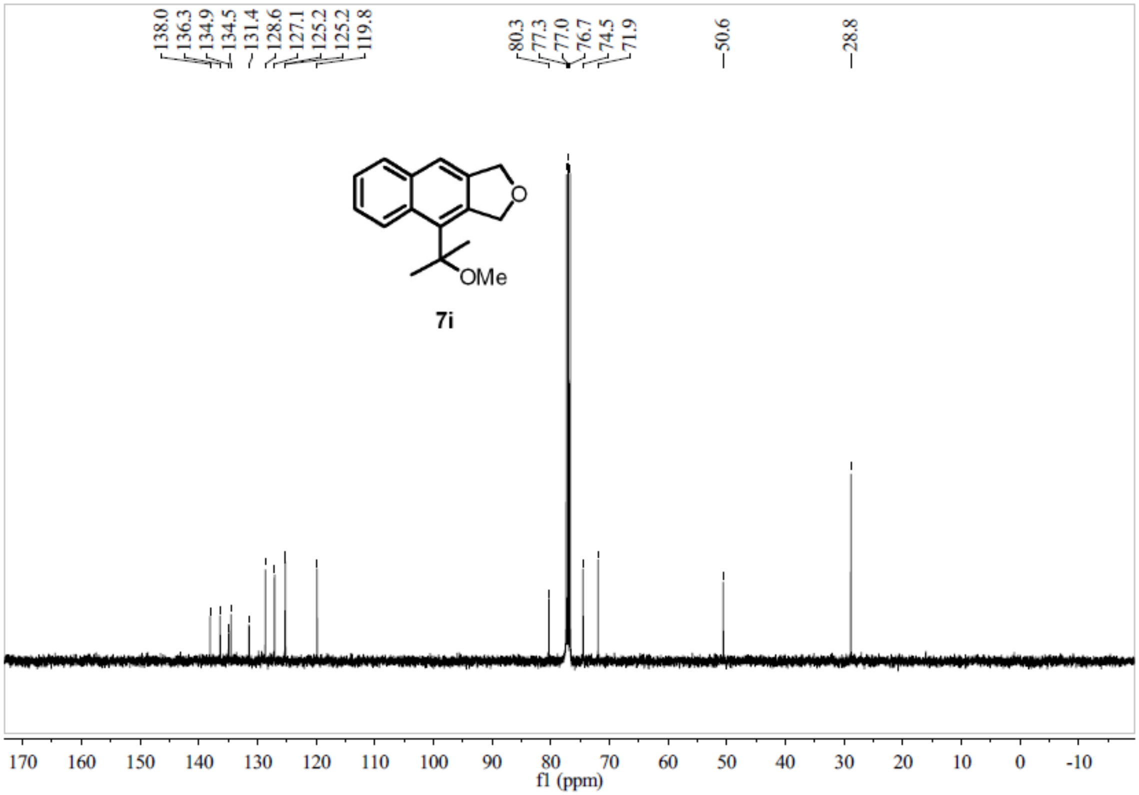

Supplementary Figure 87. ^{13}C NMR of **7i**

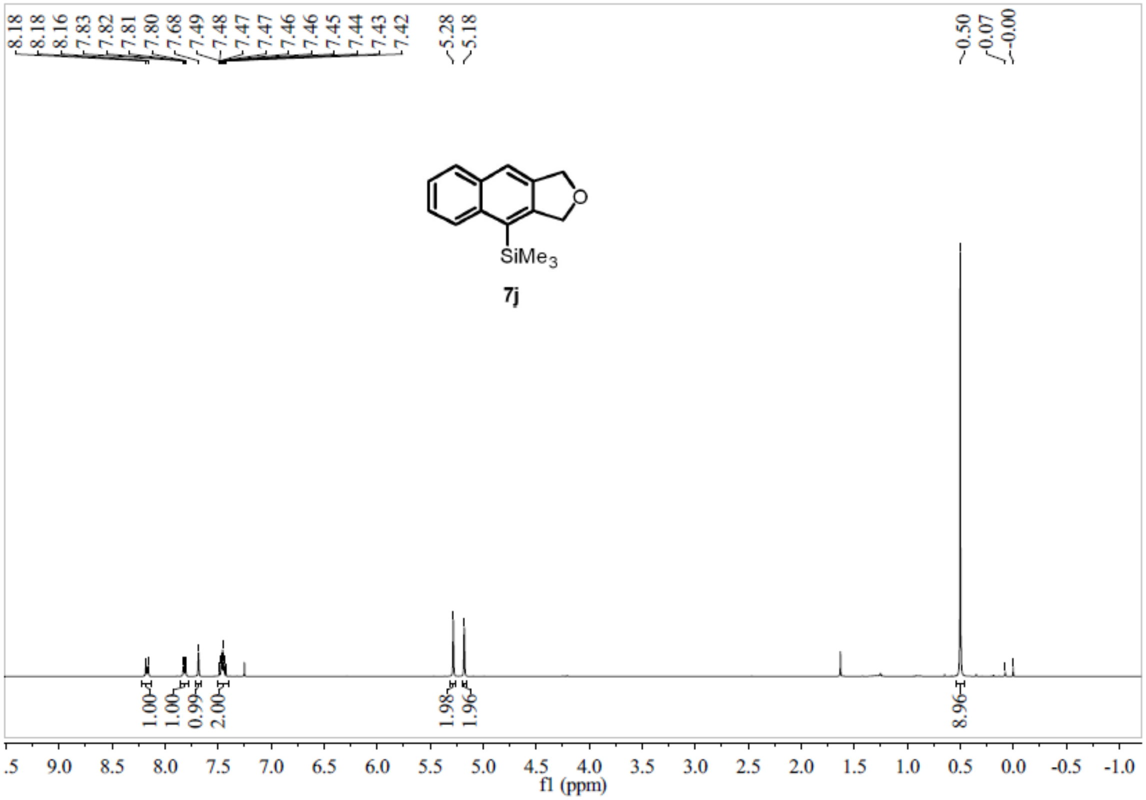

Supplementary Figure 88. ^{1}H NMR of **7j**

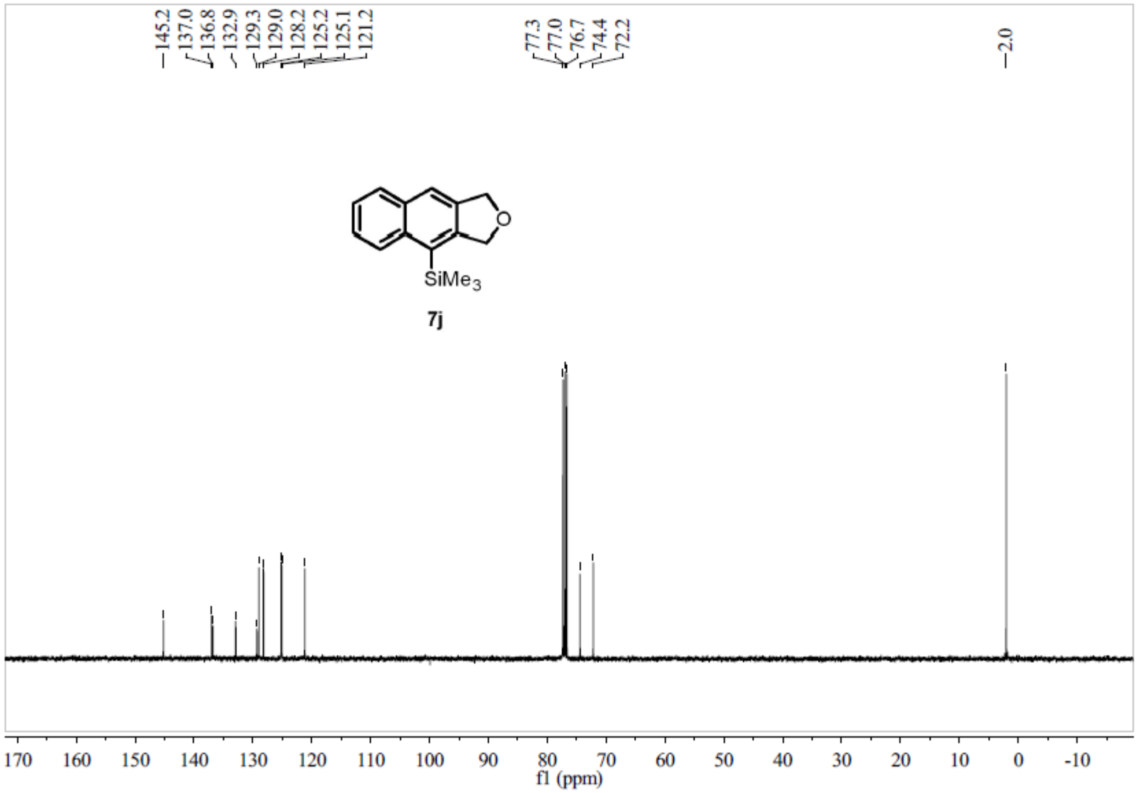

Supplementary Figure 89. ^{13}C NMR of **7j**

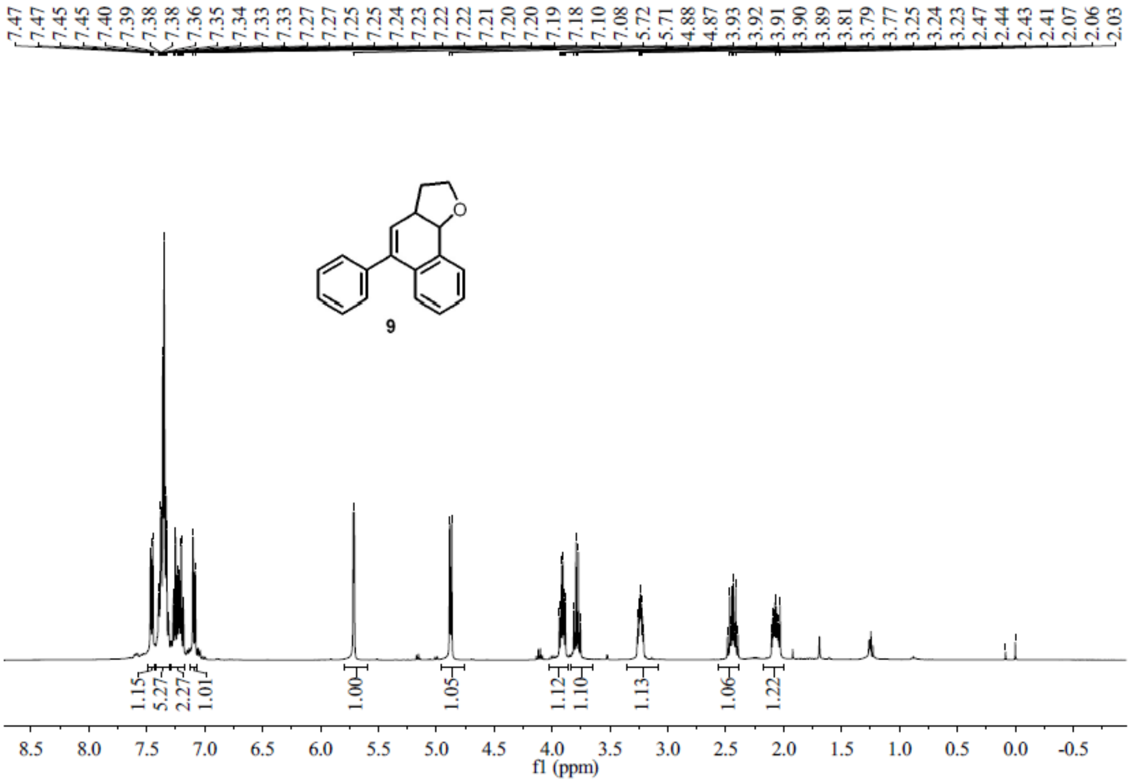

Supplementary Figure 90. ^{1}H NMR of **9**

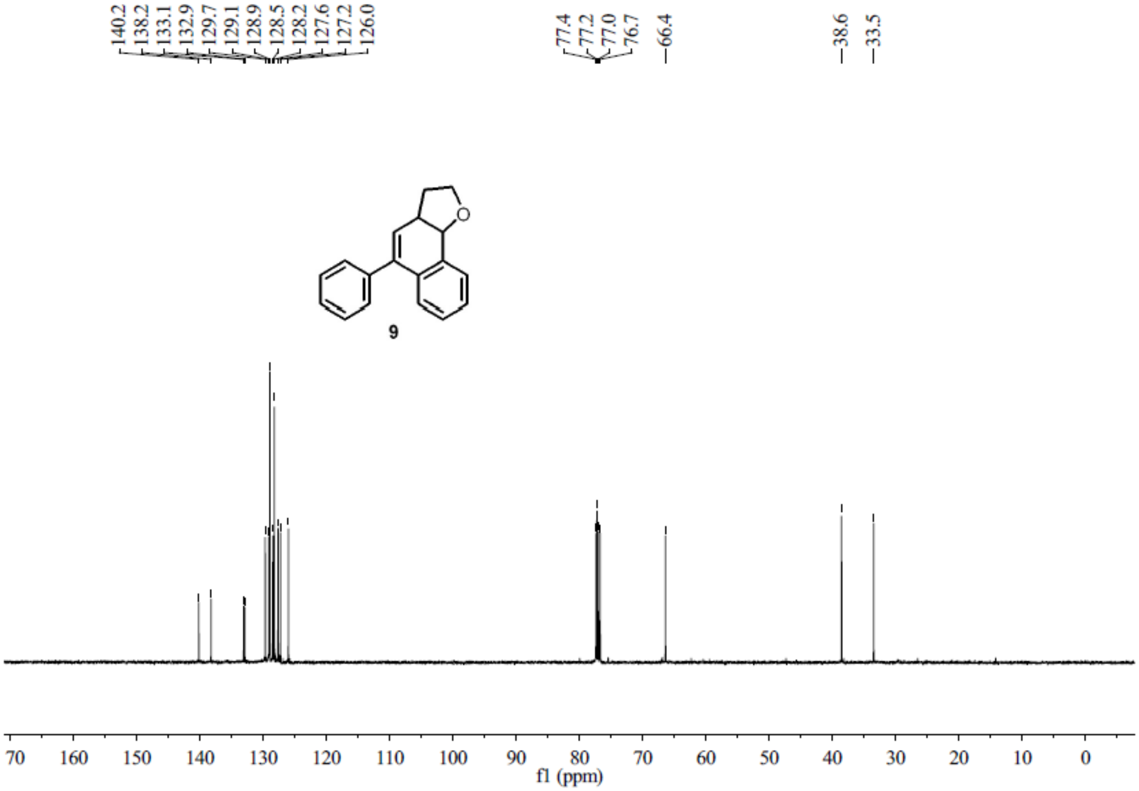

Supplementary Figure 91. ^{13}C NMR of **9**

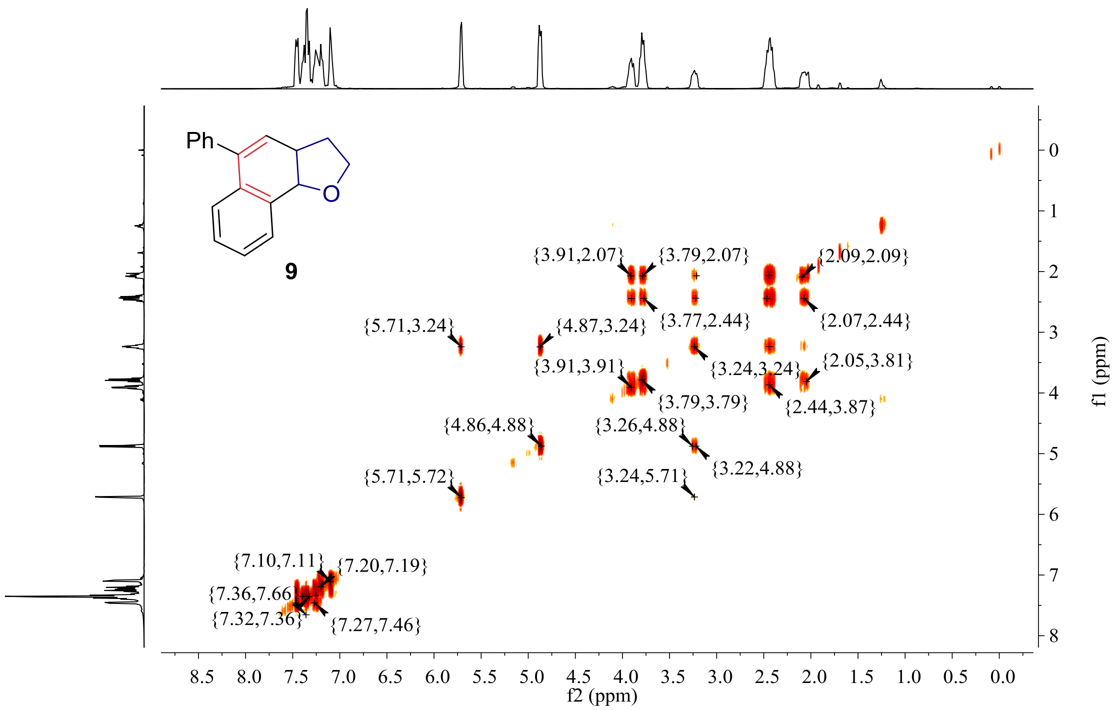

Supplementary Figure 92. ^{1}H-^{1}H COSY NMR of **9**

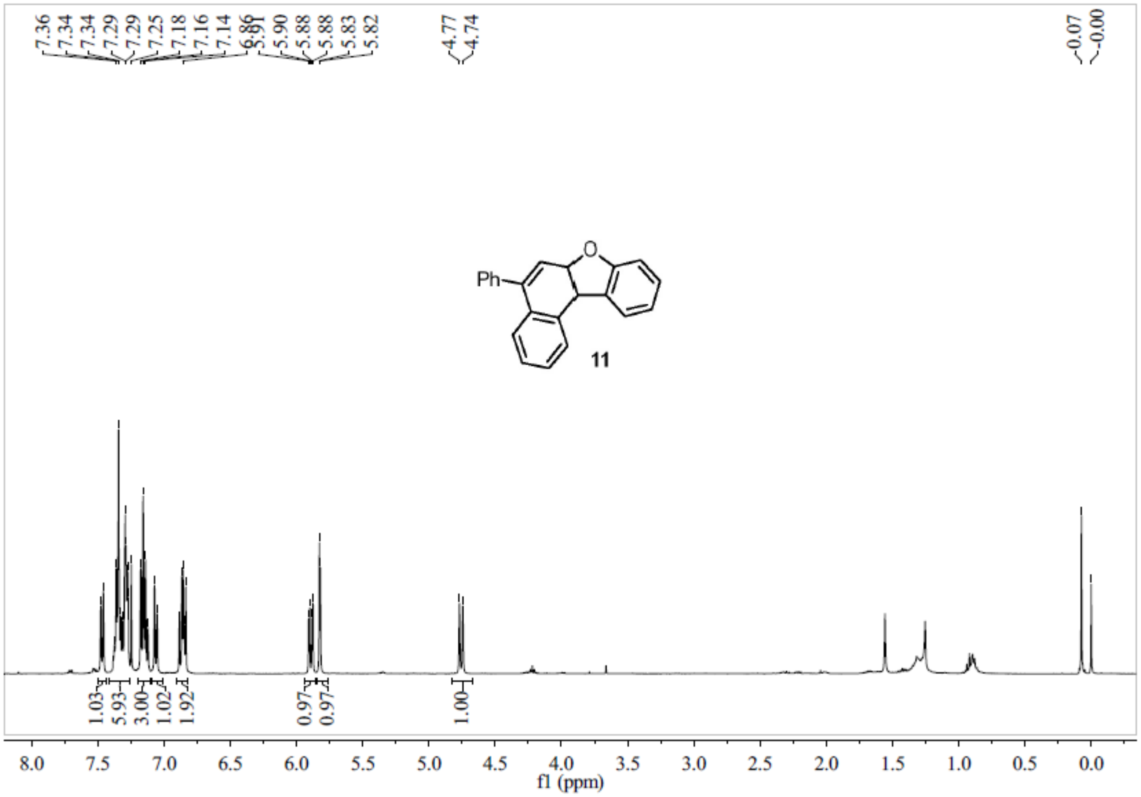

Supplementary Figure 93. ^{1}H NMR of **11**

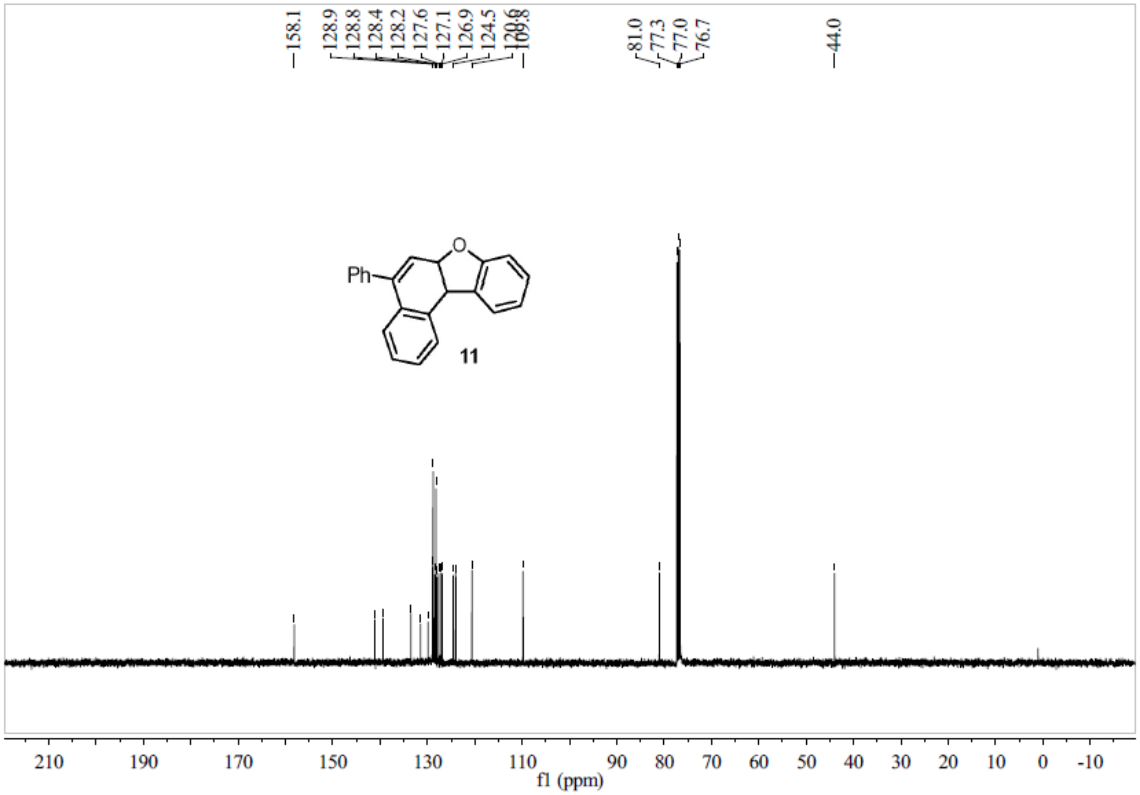

Supplementary Figure 94. ^{13}C NMR of **11**

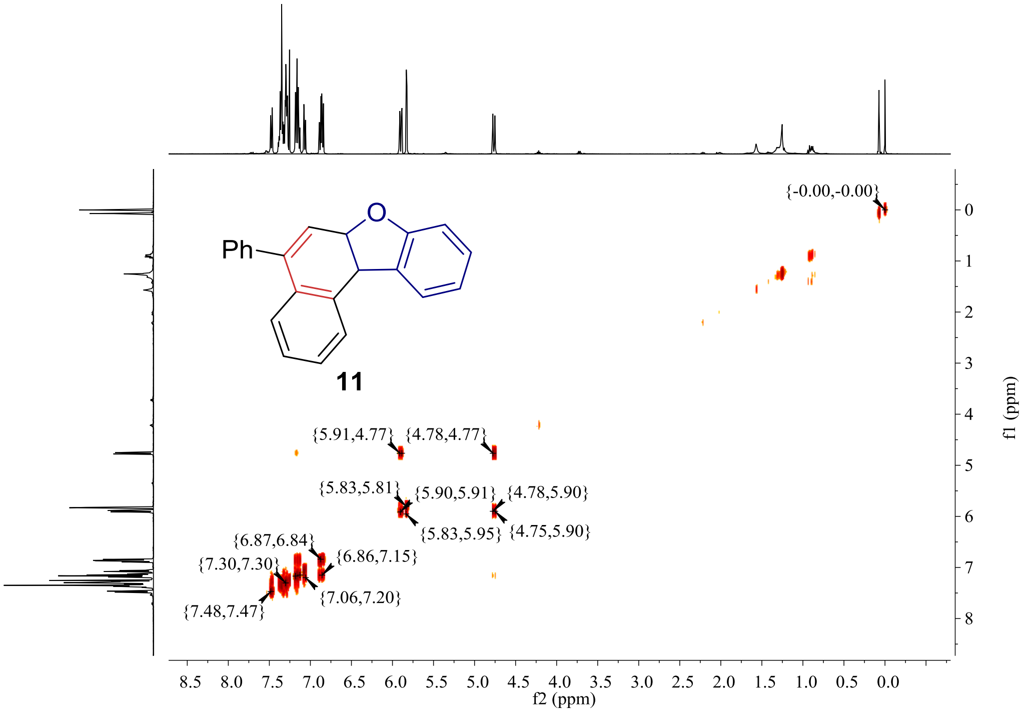

Supplementary Figure 95. ^{1}H-^{1}H COSY NMR of **11**

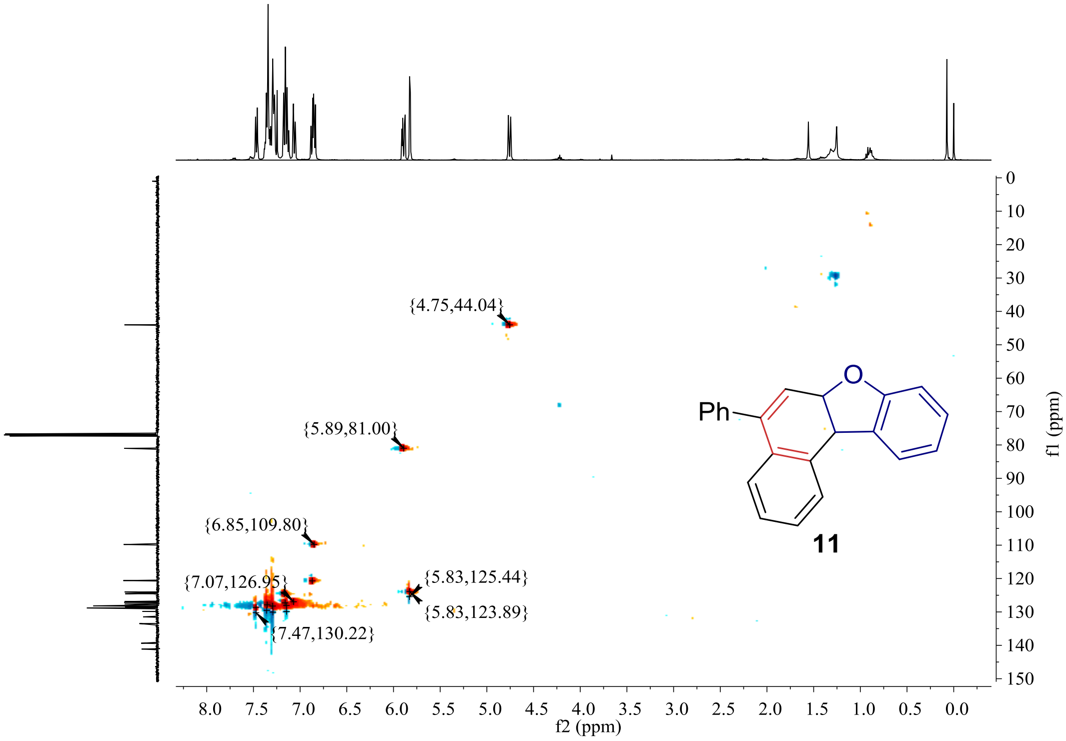

Supplementary Figure 96. HMQC spectra of **11**

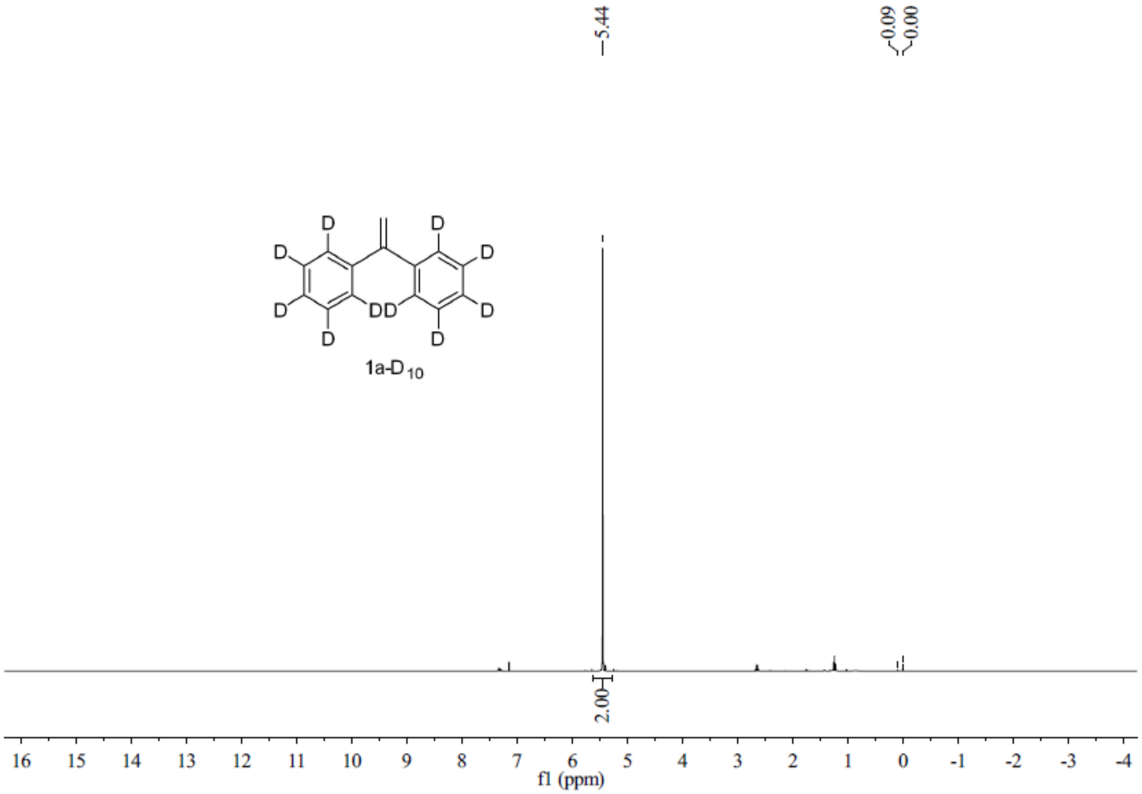

Supplementary Figure 97. ^{1}H NMR of **1a-D$_{10}$**

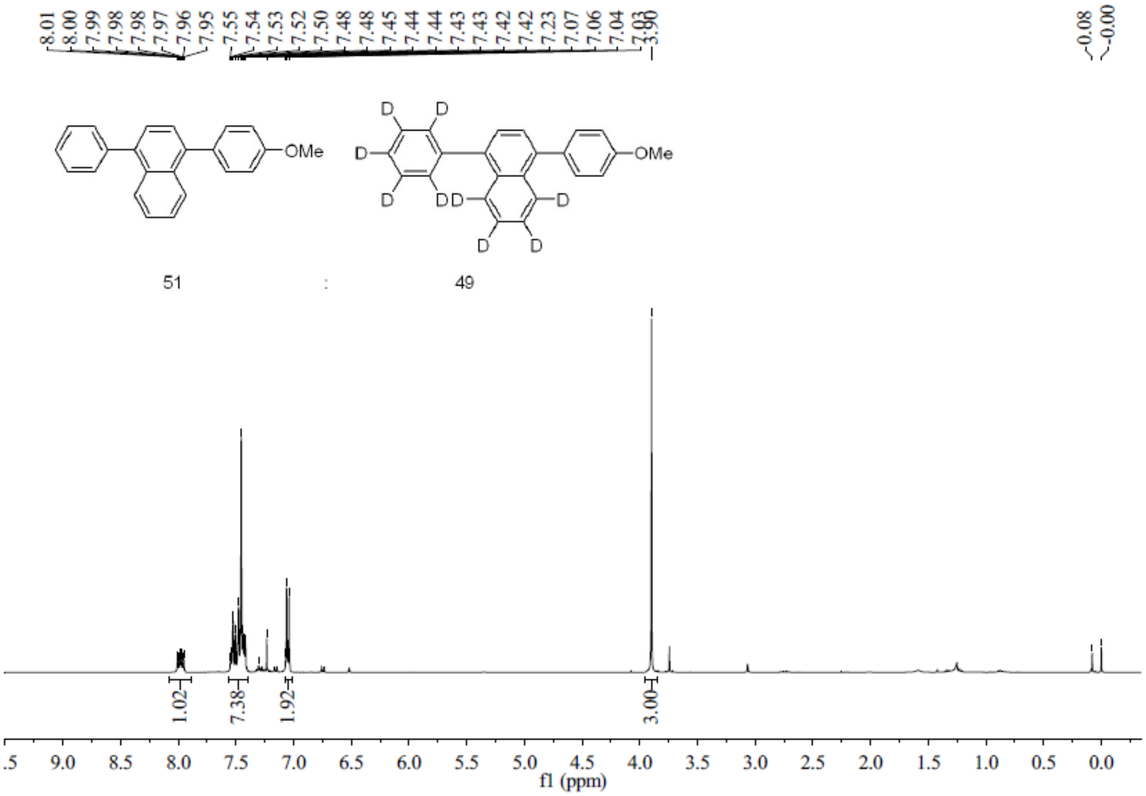

Supplementary Figure 98. The ^{1}H NMR of KIE experiment.

Supplementary Table 1. Optimization studies.[a]

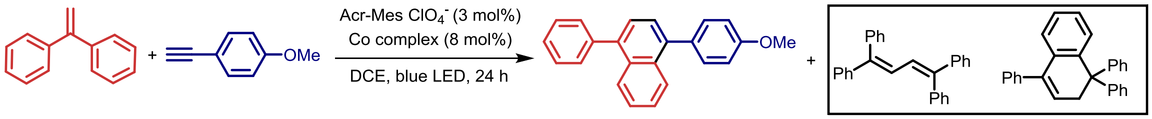

Entry	Cobalt Complex	**3aa**[b]	Dimer of **1a**[c]	H_2[d]
1	Co(dmgH)$_2$pyCl (**5S-1**)	66%	25%	35%
2	Co(dmgH)(dmgH$_2$)Cl$_2$ (**5S-2**)	66%	28%	39%
3	Co(dmgH)$_2$(4-NMe$_2$py)Cl (**5S-3**)	49%	20%	24%
4	Co(dmgH)$_2$(4-CO$_2$Mepy)Cl (**5S-4**)	69%	27%	49%
5	Co(dmgBF$_2$)$_2$(CH$_3$CN)$_2$ (**5S-5**)	67%	18%	45%
6	Co(dmgH)$_2$py$_2$PF$_6$ (**5**)	75%	25%	52%

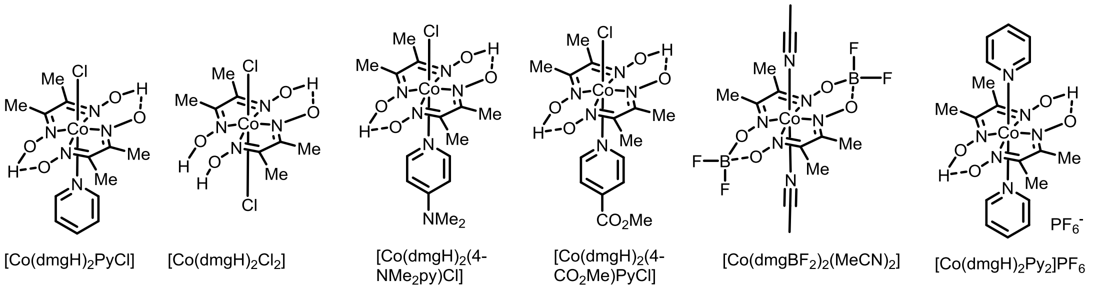

[a]Conditions: a DCE (5 mL) solution of **1a** (0.26 mmol, 1.3 equiv.), **2a** (0.2 mmol, 1 equiv.), Acr-Mes ClO_4^- (0.006 mmol, 3 mol%), cobaloxime catalyst (0.016 mmol, 8 mol%) was irradiated by blue LED for 24 h. [b]Yields were determined by FID-GC using dodecane as the internal standard. [c]The yields of dimer were based on 0.26 mmol scale. [d]The yields of H_2 were determined by TCD-GC using methane as the internal standard.

Supplementary Table 2. Optimization studies.[a]

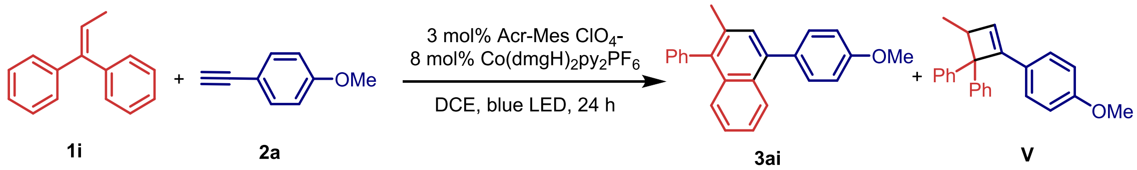

Entry	Concentration of **2a**	**3**	**V**
1	0.040 M	64%	18%
2	0.020 M	64%	18%
3	0.017 M	70%	4%
4	0.014 M	73%	2%

[a]Conditions: a DCE (5 – 14 mL) solution of **1a** (0.26 mmol, 1.3 equiv.), **2a** (0.2 mmol, 1 equiv.), Acr-Mes ClO_4^- (0.006 mmol, 3 mol%), Co(dmgH)$_2$py$_2$PF$_6$ (0.016 mmol, 8 mol%) was irradiated by blue LED for 24 h, yields were determined by FID-GC.

Supplementary Table 3. Control experiments.[a]

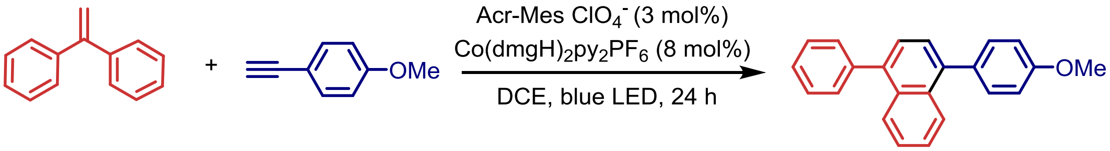

1a **2a** **3aa**

Entry	Changing from standard conditions	**3aa**
1	Standard condtions	75%
2	no photosensitizer	n.d.
3	no cobalt catalyst	n.d.
4	no light	n.d.

[a]Conditions: a DCE (14 mL) solution of **1a** (0.26 mmol, 1.3 equiv.), **2a** (0.2 mmol, 1 equiv.), Acr-Mes ClO_4^- (0.006 mmol, 3 mol%), $Co(dmgH)_2py_2PF_6$ (0.016 mmol, 8 mol%) was irradiated by blue LED for 24 h, yields were determined by FID-GC. n.d. = no desired products.

General Methods

Unless otherwise stated, analytical grade solvents and commercially available reagents were used without further purification. 9-Mesityl-10-methylacridinium perchlorate is purchased from Tokyo Chemical Industry (TCI). Dichloroethane (DCE) was dried and distilled over calcium hydride (CaH_2). Styrenes derivatives were prepared following Wittig reactions.[1] Ethynylbenzene derivatives were known compounds and prepared through Sonogashira reaction.[2]

All manipulations were carried out by using standard Schlenk techniques. All solvents were degassed prior to use. Thin layer chromatography (TLC) employed glass 0.25 mm silica gel plates. Flash chromatography columns were packed with 200−300 mesh silica gel in petroleum ether (bp. 60−90 $^{\circ}$C). Gradient flash chromatography was conducted eluting with a continuous gradient from petroleum ether to dichloromethane. All new compounds were characterized by ^{1}H NMR, ^{13}C NMR and HRMS. The known compounds were characterized by ^{1}H NMR and ^{13}C NMR. The ^{1}H and ^{13}C NMR spectra were recorded on a Bruker 400 MHz NMR spectrometer. The chemical shifts (δ) were given in part per million relative to internal tetramethyl silane (TMS, 0 ppm for ^{1}H), $CDCl_3$ (77.0 ppm for ^{13}C). High resolution mass spectra (HRMS) were measured with a Waters Micromass GCT instrument and accurate masses were reported for the molecular ion. HRMS were obtained using EI ionization, electron energy: 70 eV. Spectrum H_2 gas content was analyzed by gas chromatography (7890-II, Tianmei, China, TCD, argon as a carrier gas and 5 Å molecular sieve column, a thermal conductivity detector). The source of the blue LEDs is the LED lights made by ourselves. There is 3.0 cm distance between the reactor and LEDs. The photographs of this photochemical setup were show in the Supplementary Figure 1.

Experimental procedures

Preparation of $Co(dmgH)_2py_2PF_6$ [3].

Cobalt(II) nitrate hexahydrate (5.82 g, 0.02 mol) was dissolved in 200 mL of 80% aqueous methanol; the solution was treated with pyridine (14.24 g, 0.18 mol) and then with 4.64 g (0.04 mol) of dimethylglyoxime. The solution was stirred as air was pulled through the solution for 3.5 h. The solution became cloudy and was filtered to remove a fraction of pale pink material, which was discarded. The filtrate was treated with ammonium hexafluorophosphate (3.26 g, 0.02 mol), and the product precipitated as a light gold powder, which was collected and washed with water and methanol; yield 10.10 g (85%). The product was recrystallized from acetone by the addition of water as shiny, dark gold tablets, which were washed with water and methanol and dried in a vacuum desiccator. ^{1}H NMR is consistent with the literature.

Preparation of styrene-ynes derivatives[4]:

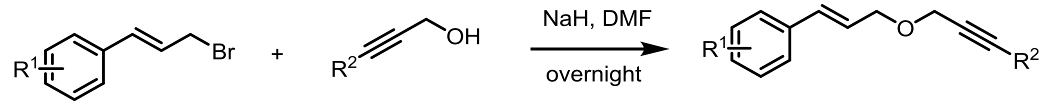

S1 **S2** **6**

Styrene-ynes **6** were prepared following literature procedures. NaH (60% dispersion in mineral oil, 0.34 g, 8.6 mmol) was taken in a dry two necked round-bottom flask and dry THF (25 mL). The reaction flask was cooled in an ice bath. Propargyl alcohol derivative **S2** (5.74 mmol) was added slowly dropwise, and the mixture was allowed to stir at 0 °C for 30 min. Then a solution of cinnamyl bromide (6.31 mmol) in dry THF (5 mL) was added slowly. Reaction was monitored by TLC and found to complete in 2 h. Excess NaH was quenched by adding few drops of water slowly. THF was evaporated, and the residue was taken in ethyl acetate (50 mL) and washed with water (40 mL) and then brine solution (50 mL). Organic layer was dried over anhydrous Na_2SO_4 and concentrated. The crude mixture was purified by column chromatography.

General procedure for oxidative [4+2] annulation of styrenes with alkynes:

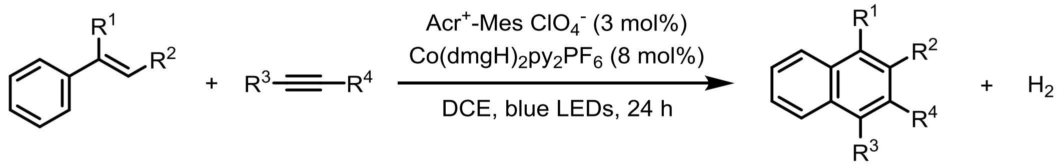

1 (1.3 equiv.) **2** (1.0 euqiv.) **3**

A schlenk tube equipped with a stir bar was loaded with 2.4 mg (3 mol%, 0.006 mmol) of Acr^+-Mes ClO_4^-, 9.5 mg (8 mol%, 0.016 mmol) of $Co(dmgH)_2py_2PF_6$, 0.26 mmol styrenes **1** and 0.2 mmol alkyne **2** in 14 mL degassed DCE under N_2 atmosphere. The solution was then stirred at room temperature under the irradiation of 12W blue LED lamp for 24 h. After the completion of reaction, the products was determined by TLC and H_2 can be detected by GC-TCD. The solvent was removed under reduced pressure by an aspirator, then the pure product was obtained by flash column chromatography on silica gel (eluent: petroleum ether/dichloromethane = 10:1) to afford corresponding naphthalene products **3**.

General procedure for intramolecular oxidative [4+2] annulation of styrene-ynes:

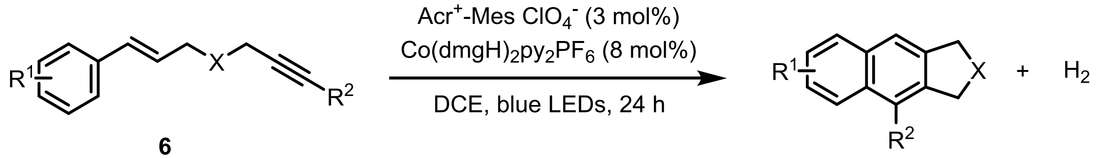

6 X = O, NTs **7**

A schlenk tube equipped with a stir bar was loaded with 2.4 mg (3 mol%, 0.006 mmol) of Acr^+-Mes ClO_4^-, 9.5 mg (8 mol%, 0.016 mmol) of $Co(dmgH)_2py_2PF_6$, 0.2 mmol styrene-ynes **6** in 5 mL degassed DCE under N_2 atmosphere. The solution was then stirred at room temperature under the irradiation of 12W blue LED lamp for 24 h. After the completion of reaction, the products were determined by TLC and H_2 can be detected

by GC-TCD. The solvent was removed under reduced pressure by an aspirator, then the pure product was obtained by flash column chromatography on silica gel (eluent: PE/EA = 10:1) to afford corresponding naphthalene products **7**.

Emission quenching experiments for Acr$^+$-Mes ClO_4^-.

Emission intensities were recorded using a HITACHI F-4500 Fluorescence Spectrometer. All Acr$^+$-Mes ClO_4^- solutions were excited at 450 nm and the emission intensity at 512 nm was observed. DCE was degassed with a stream of N_2 for 30 min and then moved to glove box. All the solutions were prepared in the glove box. In a typical experiment, the emission spectrum of a 5×10^{-4} M solution of Acr$^+$-Mes ClO_4^- in DCE was collected. Then, appropriate amount of quencher was added to the measured solution and the emission spectrum of the sample was collected.

Kinetics of isotopic effect experiments.

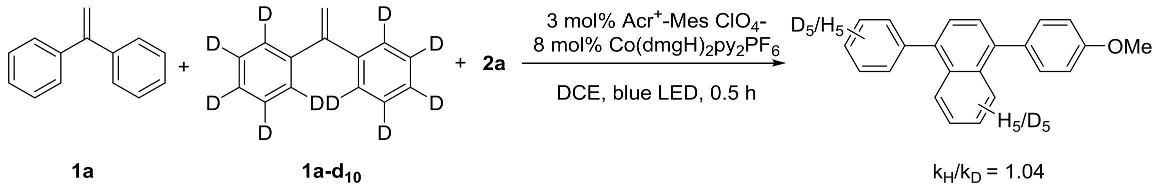

A schlenk tube equipped with a stir bar was loaded with 2.4 mg (3 mol%, 0.006 mmol) of Acr$^+$-Mes ClO_4^-, 9.5 mg (8 mol%, 0.016 mmol) of $Co(dmgH)_2py_2PF_6$, 0.13 mmol **1a,** 0.13 mmol **1a-d$_{10}$**, and 0.2 mmol **2a** in 14 mL degassed DCE under N_2 atmosphere. The solution was then stirred at room temperature under the irradiation of blue LED lamp for 0.5 h. The solvent was removed under reduced pressure by an aspirator, then the pure product was obtained by flash column chromatography on silica gel (eluent: petroleum ether/dichloromethane = 10:1) to afford the mixture of products **3aa** and **3aa-d$_{10}$** in combined 31% yield. Comparing the ^{1}H NMR spectra (Supplementary Figure 96), we found the ratio of **2aa**: **2aa-d$_{10}$** was 1.02 : 0.98. So the intermolecular KIE value was 1.04.

Analytical data of products

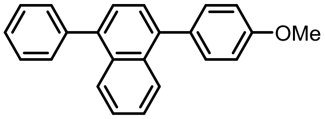

3aa

1-(4-Methoxyphenyl)-4-phenylnaphthalene (**3aa**)[5]; 46.5 mg (yield: 75%, 0.2 mmol scale), white solid. ^{1}H NMR (400 MHz, $CDCl_3$) δ 8.03 – 7.92 (m, 2H), 7.59 – 7.38 (m, 11H), 7.08 – 6.99 (m, 2H), 3.90 (s, 3H); ^{13}C NMR (100 MHz, $CDCl_3$) δ 158.9, 140.8, 139.5, 139.5, 133.1, 132.1, 131.9, 131.2, 130.1, 128.3, 127.2, 126.5, 126.4, 126.4, 126.4, 125.8, 125.7, 113.7, 55.4.

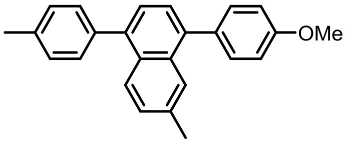

3ab

4-(4-Methoxyphenyl)-6-methyl-1-(p-tolyl)naphthalene (**3ab**); 39.9 mg, (yield: 59%, 0.2 mmol scale), white solid. ^{1}H NMR (400 MHz, $CDCl_3$) δ 7.88 (d, *J* = 8.6 Hz, 1H), 7.74 (s, 1H), 7.49 – 7.42 (m, 3H), 7.39 (dd, *J* = 11.3, 3.9 Hz, 3H), 7.31 (d, *J* = 7.9 Hz, 2H), 7.24 (d, *J* = 2.4 Hz, 1H), 7.08 – 7.01 (m, 2H), 3.91 (s, 3H), 2.46 (s, 3H), 2.43 (s, 3H); ^{13}C NMR (100 MHz, $CDCl_3$) δ 158.9, 139.4, 138.6, 138.1, 136.9, 135.4, 133.4, 132.3, 131.2, 130.2, 130.0, 129.0, 128.8, 128.0, 127.9, 127.0, 126.7, 126.4, 125.6, 125.3, 113.7, 55.4, 21.9, 21.3, 21.1. HRMS (EI) calcd for $C_{25}H_{22}O^+$ $[M]^+$, 338.1671, found: 338.1676.

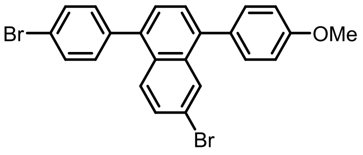

3ac

6-Bromo-1-(4-bromophenyl)-4-(4-methoxyphenyl)naphthalene (**3ac**); 51.2 mg, (yield: 55%, 0.2 mmol scale), white solid. ^{1}H NMR (400 MHz, $CDCl_3$) δ 8.12 (s, 1H), 7.75 (d, *J* = 9.0 Hz, 1H), 7.63 (d, *J* = 8.1 Hz, 2H), 7.51 – 7.37 (m, 7H), 7.06 (d, *J* = 8.4 Hz, 2H), 3.91 (s, 3H); ^{13}C NMR (100 MHz, $CDCl_3$) δ 159.2, 139.1, 139.1, 138.2, 133.4, 132.1 131.6, 131.6, 131.0, 130.2, 129.3, 128.6, 127.8, 127.4, 126.8, 121.7, 120.5, 114.0, 55.4. HRMS (ESI) calcd for $C_{23}H_{17}Br_2O^+$ $[M+H]^+$, 466.9641, found: 466.9641.

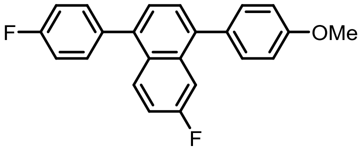

3ad

6-Fluoro-1-(4-fluorophenyl)-4-(4-methoxyphenyl)naphthalene (**3ad**); 44.0 mg (yield: 66%, 0.2 mmol scale), white solid. ^{1}H NMR (400 MHz, $CDCl_3$) δ 7.88 (dd, *J* = 9.3, 5.9 Hz, 1H), 7.60 (dd, *J* = 11.2, 2.6 Hz, 1H), 7.52 – 7.33 (m, 6H), 7.27 – 7.15 (m, 3H), 7.11 – 7.00 (m, 2H), 3.90 (s, 3H); ^{13}C NMR (100 MHz, $CDCl_3$) δ 162.72 (d, *J* = 164.6 Hz), 160.28 (d, *J* = 164.0 Hz), 159.1, 139.16 (d, *J* = 5.5 Hz), 138.5, 136.47 (d, *J* = 3.4 Hz), 133.29 (d, *J* = 8.7 Hz), 132.5, 131.54 (d, *J* = 8.0 Hz), 130.1, 129.0, 128.76 (d, *J* = 8.8 Hz), 127.4, 125.82 (d, *J* = 2.0 Hz), 116.04 (d, *J* = 25.0 Hz), 115.31 (d, *J* = 21.3 Hz), 113.9, 109.9 (d, *J* = 21.8 Hz), 55.4. HRMS (EI) calcd for $C_{23}H_{16}F_2O^+$ $[M]^+$, 346.1169, found: 346.1176.

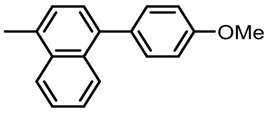

3ae

1-Methyl-3,4-dihydronaphthalen-2(1H)-one (**3ae**); 23.8 mg (yield: 48%, 0.2 mmol scale), white solid. ^{1}H NMR (400 MHz, $CDCl_3$) δ 8.05 (d, *J* = 8.3 Hz, 1H), 7.93 (d, *J* = 8.4 Hz, 1H), 7.52 (dd, *J* = 8.3, 1.3 Hz, 1H), 7.48 – 7.37 (m, 3H), 7.36 (d, *J* = 7.2 Hz, 1H), 7.30 (d, *J* = 7.1 Hz, 1H), 7.12 – 6.89 (m, 2H), 3.88 (s, 3H), 2.73 (s, 3H); ^{13}C NMR (100 MHz, $CDCl_3$) δ 158.8, 138.3, 133.5, 133.3, 132.8, 131.9, 131.2, 126.7, 126.6, 126.2, 125.5, 125.5, 124.4, 113.6, 55.3, 19.6. HRMS (EI) calcd for $C_{18}H_{16}O^+$ $[M]^+$, 248.1201, found: 248.1211.

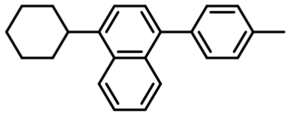

3af

1-Cyclohexyl-4-(p-tolyl)naphthalene (**3af**); 37.2 mg (yield: 62%, 0.2 mmol scale), white solid. ^{1}H NMR (400 MHz, $CDCl_3$) δ 8.18 (d, *J* = 8.5 Hz, 1H), 7.95 (d, *J* = 8.4 Hz, 1H), 7.51 (t, *J* = 7.5 Hz, 1H), 7.47 – 7.21 (m, 7H), 3.45 – 3.30 (m, 1H), 2.45 (s, 3H), 2.19 – 3.05 (m, 2H), 2.00 – 1.90 (m, 2H), 1.87 – 1.79 (m, 1H), 1.72 – 1.49 (m, 4H), 1.43 – 1.20 (m, 1H); ^{13}C NMR (100 MHz, $CDCl_3$) δ 143.1, 138.2, 138.2, 136.6, 132.0, 131.5, 130.1, 128.9, 127.0, 126.7, 125.4, 125.2, 123.4, 121.9, 39.3, 34.2, 27.3, 26.5, 21.2. HRMS (EI) calcd for $C_{23}H_{24}^+$ $[M]^+$, 300.1878, found: 300.1880.

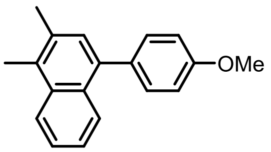

3ag

4-(4-Methoxyphenyl)-1,2-dimethylnaphthalene (**3ag**); 39.3 mg (yield: 75%, 0.2 mmol scale), white solid. ^{1}H NMR (400 MHz, $CDCl_3$) δ 8.08 (d, *J* = 8.5 Hz, 1H), 7.88 (d, *J* =

8.4 Hz, 1H), 7.55 – 7.46 (m, 1H), 7.44 – 7.32 (m, 3H), 7.24 (s, 1H), 7.04 – 6.96 (m, 2H), 3.88 (s, 3H), 2.63 (s, 3H), 2.51 (s, 3H); ^{13}C NMR (100 MHz, $CDCl_3$) δ 158.7, 137.5, 133.3, 133.0, 132.6, 131.2, 130.5, 130.4, 130.1, 126.5, 125.6, 124.5, 123.9, 113.6, 55.3, 20.8, 14.6. HRMS (EI) calcd for $C_{19}H_{18}O^+$ $[M]^+$, 262.1358, found: 262.1369.

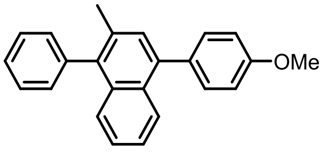

3ah

4-(4-Methoxyphenyl)-2-methyl-1-phenylnaphthalene (**3ah**)[6]; 47.3 mg (yield: 73%, 0.2 mmol scale), white solid. ^{1}H NMR (400 MHz, $CDCl_3$) δ 7.93 (dd, J = 7.1, 2.4 Hz, 1H), 7.58 – 7.41 (m, 6H), 7.39 – 7.28 (m, 5H), 7.05 (d, J = 8.6 Hz, 2H), 3.91 (s, 3H), 2.26 (s, 3H); ^{13}C NMR (100 MHz, $CDCl_3$) δ 158.9, 139.8, 139.0, 137.5, 133.3, 133.1, 132.7, 131.1, 130.2, 130.2, 129.7, 128.4, 127.0, 126.5, 125.9, 125.6, 124.7, 113.7, 55.4, 20.8.

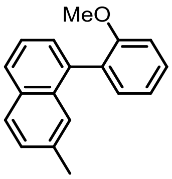

3ai

1-(2-Methoxyphenyl)-7-methylnaphthalene (**3ai**); 20.8 mg (yield: 42%, 0.2 mmol scale), white solid. ^{1}H NMR (400 MHz, $CDCl_3$) δ 7.85 – 7.74 (m, 2H), 7.48 – 7.40 (m, 2H), 7.36 (dd, J = 7.0, 1.2 Hz, 1H), 7.33 (s, 1H), 7.31 – 7.26 (m, 2H), 7.13 – 7.03 (m, 2H), 3.70 (s, 3H), 2.40 (s, 3H); ^{13}C NMR (100 MHz, $CDCl_3$) δ 157.3, 136.2, 135.3, 132.2, 132.0, 131.7, 129.7, 128.8, 127.9, 127.8, 127.4, 127.4, 125.2, 124.4, 120.5, 111.0, 55.6, 21.9. HRMS (EI) calcd for $C_{18}H_{16}O^+$ $[M]^+$, 248.1201, found: 248.1195.

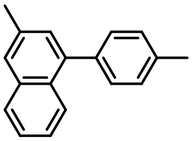

3aj

3-Methyl-1-(p-tolyl)naphthalene (**3aj**); 20.0 mg (yield: 43%, 0.2 mmol scale), white solid. ^{1}H NMR (400 MHz, $CDCl_3$) δ 7.86 (d, J = 8.4 Hz, 1H), 7.81 (dd, J = 8.2, 2.9 Hz, 1H), 7.62 (s, 1H), 7.49 – 7.42 (m, 1H), 7.42 – 7.36 (m, 3H), 7.30 (d, J = 7.8 Hz, 3H), 2.54 (s, 3H), 2.46 (s, 3H); ^{13}C NMR (100 MHz, $CDCl_3$) δ 140.0, 137.8, 136.8, 134.9, 134.1, 129.91, 129.87, 129.2, 128.9, 127.6, 126.4, 125.9, 125.7, 125.0, 21.7, 21.2. HRMS (EI) calcd for $C_{18}H_{16}^+$ $[M]^+$, 232.1252, found: 232.1257.

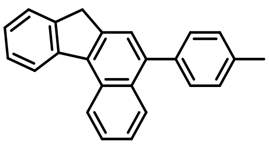

3ak

5-(p-Tolyl)-7H-benzo[c]fluorene (**3ak**); 34.3 mg (yield: 56%, 0.2 mmol scale), white solid. ^{1}H NMR (400 MHz, $CDCl_3$) δ 8.84 (d, *J* = 8.4 Hz, 1H), 8.42 (d, *J* = 7.8 Hz, 1H), 8.03 (d, *J* = 8.5 Hz, 1H), 7.71 – 7.58 (m, 3H), 7.55 – 7.45 (m, 2H), 7.43 (d, *J* = 8.0 Hz, 2H), 7.38 – 7.30 (m, 3H), 4.04 (s, 2H), 2.47 (s, 3H); ^{13}C NMR (100 MHz, $CDCl_3$) δ 144.2, 142.7, 141.8, 139.7, 138.2, 136.9, 135.4, 131.6, 130.1, 129.9, 129.0, 127.4, 126.9, 126.3, 125.7, 125.0, 124.9, 124.4, 123.9, 122.9, 37.8, 21.3. HRMS (EI) calcd for $C_{24}H_{18}^{+}$ $[M]^{+}$, 306.1409, found: 306.1418.

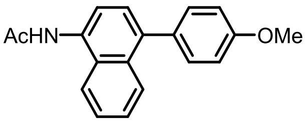

3al

N-(4-(4-Methoxyphenyl)naphthalen-1-yl)acetamide (**3al**); 32.0 mg (yield: 55%, 0.2 mmol scale), white solid. ^{1}H NMR (400 MHz, DMSO-d_6) δ 10.01 (s, 1H), 8.15 (d, *J* = 8.4 Hz, 1H), 7.84 (d, *J* = 8.4 Hz, 1H), 7.71 (d, *J* = 7.6 Hz, 1H), 7.57 (t, *J* = 7.3 Hz, 1H), 7.54 – 7.45 (m, 1H), 7.38 (dd, *J* = 8.0, 5.0 Hz, 3H), 7.09 (d, *J* = 8.6 Hz, 2H), 3.83 (s, 3H), 2.21 (s, 3H); ^{13}C NMR (100 MHz, DMSO-d_6) δ 169.2, 158.7, 136.6, 133.1, 132.2, 131.7, 131.0, 128.2, 126.6, 126.3, 125.9, 125.7, 123.3, 121.6, 114.0, 55.3, 23.6. HRMS (ESI) calcd for $C_{19}H_{18}NO_2^{+}$ $[M+H]^{+}$, 292.1332, found: 292.1331.

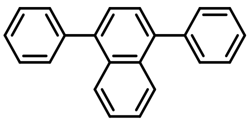

3ba

1,4-Diphenylnaphthalene (**3ba**)[7]; 26.9 mg (yield: 48%, 0.2 mmol scale), white solid. ^{1}H NMR (400 MHz, $CDCl_3$) δ 7.97 (dd, *J* = 6.4, 3.3 Hz, 2H), 7.61 – 7.36 (m, 14H); ^{13}C NMR (100 MHz, $CDCl_3$) δ 140.8, 139.8, 131.9, 130.1, 128.3, 127.2, 126.4, 126.4, 125.8.

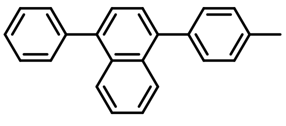

3bb

1-Phenyl-4-(p-tolyl)naphthalene (**3bb**)[7]; 30.6 mg (yield: 52%, 0.2 mmol scale), white solid.

^{1}H NMR (400 MHz, $CDCl_3$) δ 7.99 (dd, *J* = 6.7, 3.3 Hz, 1H), 7.96 (dd, *J* = 6.6, 3.3 Hz, 1H), 7.64 – 7.38 (m, 11H), 7.32 (d, *J* = 7.8 Hz, 2H), 2.46 (s, 3H); ^{13}C NMR (100 MHz, $CDCl_3$) δ 140.8, 139.8, 139.6, 137.8, 136.9, 131.9, 131.9, 130.1, 130.0, 129.0, 128.3,

127.2, 126.5, 126.42, 126.40, 126.3, 125.8, 125.7, 21.3.

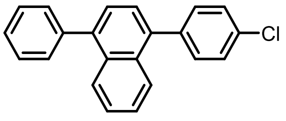

3bc

1-(4-Chlorophenyl)-4-phenylnaphthalene (**3bc**); 22.6mg (yield: 36%, 0.2 mmol scale), white solid. ^{1}H NMR (400 MHz, $CDCl_3$) δ 8.07 – 8.00 (m, 1H), 8.00 – 7.93 (m, 1H), 7.64 – 7.46 (m, 13H); ^{13}C NMR (100 MHz, $CDCl_3$) δ 140.6, 140.2, 139.2, 138.5, 133.3, 131.9, 131.7, 131.4, 130.1, 128.5, 128.3, 127.3, 126.49, 126.44, 126.39, 126.04, 126.00, 125.96. HRMS (EI) calcd for $C_{22}H_{15}Cl^+$ $[M]^+$, 314.0862, found: 314.0876.

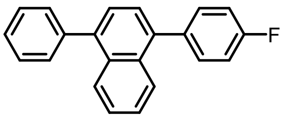

3bd

1-(4-Fluorophenyl)-4-phenylnaphthalene (**3bd**); 14.3 mg (yield: 24%, 0.2 mmol scale), white solid. ^{1}H NMR (400 MHz, $CDCl_3$) δ 7.97 (dd, *J* = 6.9, 2.9 Hz, 1H), 7.91 (dd, *J* = 6.9, 2.8 Hz, 1H), 7.57 – 7.36 (m, 11H), 7.20 (dd, *J* = 15.1, 6.4 Hz, 2H); ^{13}C NMR (100 MHz, $CDCl_3$) δ 162.26 (d, *J* = 246.2 Hz), 140.7, 140.0, 138.7, 136.7 (d, *J* = 3.3 Hz), 131.92, 131.90, 131.6 (d, *J* = 7.9 Hz), 130.1, 128.3, 127.3, 126.52, 126.45, 126.4, 126.1, 126.0, 125.9, 115.2 (d, *J* = 21.3 Hz). HRMS (EI) calcd for $C_{22}H_{15}F^+$ $[M]^+$, 298.1158, found: 298.1164.

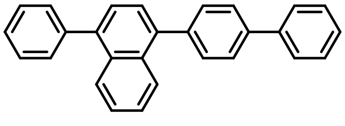

3be

1-([1,1'-Biphenyl]-4-yl)-4-phenylnaphthalene (**3be**); 64.0 mg (yield: 90%, 0.2 mmol scale), white solid. ^{1}H NMR (400 MHz, $CDCl_3$) δ 8.11 – 8.04 (m, 1H), 8.03 – 7.94 (m, 1H), 7.74 (d, *J* = 7.8 Hz, 2H), 7.70 (d, *J* = 7.9 Hz, 2H), 7.61 (d, *J* = 7.7 Hz, 2H), 7.57 – 7.41 (m, 11H), 7.38 (t, *J* = 7.3 Hz, 1H); ^{13}C NMR (100 MHz, $CDCl_3$) δ140.78, 140.75, 140.1, 139.9, 139.8, 139.4, 131.91, 131.85, 130.6, 130.1, 128.8, 128.3, 127.4, 127.3, 127.1, 127.0, 126.48, 126.47, 126.41, 126.35, 125.90, 125.88. HRMS (EI) calcd for $C_{28}H_{20}^+$ $[M]^+$, 356.1565, found: 356.1563.

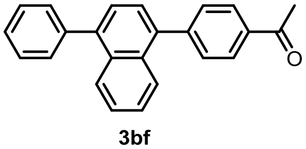

3bf

1-(4-(4-Phenylnaphthalen-1-yl)phenyl)ethan-1-one (**3bf**); 25.8 mg (yield: 40%, 0.2 mmol scale), white solid. ^{1}H NMR (400 MHz, $CDCl_3$) δ 8.15 – 8.08 (m, 2H), 7.98 (dd, *J* = 6.6,

3.1 Hz, 1H), 7.91 (dd, J = 6.6, 3.1 Hz, 1H), 7.69 – 7.61 (m, 2H), 7.56 – 7.42 (m, 9H), 2.70 (s, 3H); ^{13}C NMR (100 MHz, $CDCl_3$) δ 197.9, 145.8, 140.54, 140.50, 138.5, 135.9, 131.9, 131.5, 130.4, 130.0, 128.4, 128.3, 127.4, 126.5, 126.42, 126.37, 126.2, 126.0, 125.9, 26.7. HRMS (EI) calcd for $C_{24}H_{18}O^+$ [M]$^+$, 322.1358, found: 322.1368.

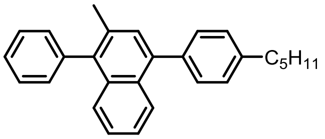

3bg

2-Methyl-4-(4-pentylphenyl)-1-phenylnaphthalene (**3bg**), 48.8 mg (yield: 67%, 0.2 mmol scale), white solid. ^{1}H NMR (400 MHz, $CDCl_3$) δ 7.99 – 7.88 (m, 1H), 7.55 – 7.41 (m, 6H), 7.37 (s, 1H), 7.35 – 7.28 (m, 6H), 2.89 – 2.59 (m, 2H), 2.25 (s, 3H), 1.72 (s, 2H), 1.50 – 1.31 (m, 4H), 1.04 – 0.82 (m, 3H); ^{13}C NMR (100 MHz, $CDCl_3$) δ 141.9, 139.8, 139.4, 138.0, 137.5, 133.3, 132.6, 130.2, 130.1, 129.9, 129.6, 128.4, 128.3, 127.0, 126.4, 126.0, 125.6, 124.7, 35.7, 31.6, 31.3, 22.6, 20.8, 14.1. HRMS (EI) calcd for $C_{28}H_{28}^+$ [M]$^+$, 364.2191, found: 364.2195.

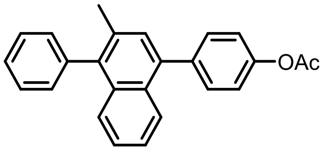

3bh

4-(3-Methyl-4-phenylnaphthalen-1-yl)phenyl acetate (**3bh**), 34.5 mg (yield: 49%, 0.2 mmol scale), white solid. ^{1}H NMR (400 MHz, $CDCl_3$) δ 7.94 (dd, J = 7.2, 2.3 Hz, 1H), 7.65 – 7.53 (m, 4H), 7.53 – 7.45 (m, 2H), 7.42 – 7.31 (m, 5H), 7.31 – 7.21 (m, 2H), 2.41 (s, 3H), 2.30 (s, 3H); ^{13}C NMR (100 MHz, $CDCl_3$) δ 169.7, 149.9, 139.7, 138.39, 138.37, 137.9, 133.2, 132.6, 131.1, 130.1, 130.0, 129.8, 128.4, 127.1, 126.5, 125.72, 125.70, 124.9, 121.4, 21.2, 20.8. HRMS (EI) calcd for $C_{25}H_{20}O_2^+$ [M]$^+$, 352.1463, found: 352.1456.

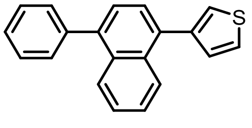

3bi

3-(4-Phenylnaphthalen-1-yl)thiophene (**3bi**), 28.6 mg (yield: 50%, 0.2 mmol scale), white solid. ^{1}H NMR (400 MHz, $CDCl_3$) δ 8.10 (d, J = 7.5 Hz, 1H), 7.96 (d, J = 7.4 Hz, 1H), 7.69 – 7.28 (m, 12H); ^{13}C NMR (100 MHz, $CDCl_3$) δ 141.1, 140.7, 139.9, 134.5, 132.1, 131.9, 130.1, 129.7, 128.3, 127.3, 126.48, 126.45, 126.4, 126.2, 126.0, 125.9, 125.3, 123.6. HRMS (EI) calcd for $C_{20}H_{14}S^+$ [M]$^+$, 286.0816, found: 286.0824.

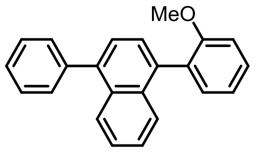

3bj

1-(2-Methoxyphenyl)-4-phenylnaphthalene (**3bj**)[6], 57.7 mg (yield: 93%, 0.2 mmol scale), white solid. ^{1}H NMR (400 MHz, $CDCl_3$) δ 7.95 (dd, *J* = 7.9, 1.8 Hz, 1H), 7.65 (dd, *J* = 7.7, 1.9 Hz, 1H), 7.57 – 7.53 (m, 2H), 7.52 – 7.47 (m, 2H), 7.46 (d, *J* = 3.0 Hz, 2H), 7.45 – 7.42 (m, 2H), 7.41 – 7.35 (m, 2H), 7.33 (dd, *J* = 7.4, 1.7 Hz, 1H), 7.17 – 6.98 (m, 2H), 3.73 (s, 3H); ^{13}C NMR (100 MHz, $CDCl_3$) δ 157.2, 140.9, 139.7, 136.5, 132.3, 132.0, 131.6, 130.2, 129.5, 129.0, 128.2, 127.1, 126.8, 126.7, 126.5, 126.2, 125.6, 125.5, 120.6, 110.9, 55.6.

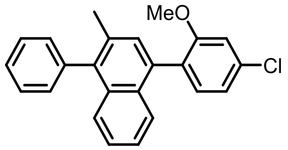

3bk

4-(4-Chloro-2-methoxyphenyl)-2-methyl-1-phenylnaphthalene (**3bk**), 35.1 mg (yield: 49%, 0.2 mmol scale), white solid. ^{1}H NMR (400 MHz, $CDCl_3$) δ 7.57 – 7.48 (m, 4H), 7.48 – 7.40 (m, 3H), 7.38 – 7.28 (m, 5H), 6.94 (d, *J* = 8.8 Hz, 1H), 3.73 (s, 3H), 2.25 (s, 3H); ^{13}C NMR (100 MHz, $CDCl_3$) δ 156.4, 139.7, 138.2, 134.6, 134.5, 132.9, 132.6, 131.7, 131.5, 130.22, 130.14, 129.9, 128.4, 127.0, 126.4, 125.9, 125.5, 124.7, 112.7, 112.5, 55.9, 20.8. HRMS (EI) calcd for $C_{24}H_{19}ClO^+$ $[M]^+$, 358.1124, found: 358.1136.

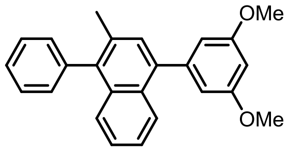

3bl

4-(4-Chloro-2-methoxyphenyl)-2-methyl-1-phenylnaphthalene (**3bl**), 35.1 mg (yield: 49%, 0.2 mmol scale), white solid. ^{1}H NMR (400 MHz, $CDCl_3$) δ 8.04 – 7.95 (m, 1H), 7.62 – 7.54 (m, 2H), 7.52 – 7.47 (m, 2H), 7.43 (d, *J* = 5.3 Hz, 1H), 7.41 – 7.33 (m, 4H), 6.75 (d, *J* = 2.3 Hz, 2H), 6.60 (t, *J* = 2.3 Hz, 1H), 3.89 (s, 6H), 2.30 (s, 3H); ^{13}C NMR (100 MHz, $CDCl_3$) δ 160.5, 142.8, 139.7, 139.3, 137.9, 133.2, 132.6, 130.2, 130.0, 129.3, 128.4, 127.0, 126.4, 125.9, 125.7, 124.9, 108.2, 99.4, 55.4, 20.8. HRMS (EI) calcd for $C_{25}H_{22}O_2^+$ $[M]^+$, 354.1620, found: 354.1619.

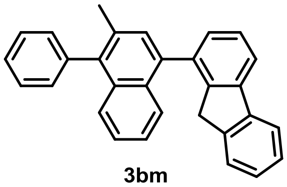

3bm

4-(4-Chloro-2-methoxyphenyl)-2-methyl-1-phenylnaphthalene (**3bm**), 39.7 mg (yield: 52%, 0.2 mmol scale), white solid. ^{1}H NMR (400 MHz, $CDCl_3$) δ 8.09 – 8.02 (m, 1H), 7.98 (d, *J* = 7.8 Hz, 1H), 7.93 (d, *J* = 7.5 Hz, 1H), 7.79 (d, *J* = 0.5 Hz, 1H), 7.70 – 7.45 (m, 8H), 7.44 – 7.36 (m, 5H), 4.07 (s, 2H), 2.35 (s, 3H); ^{13}C NMR (100 MHz, $CDCl_3$) δ 143.4, 143.3, 141.4, 140.8, 139.8, 139.7, 139.3, 137.7, 133.3, 132.6, 130.2, 129.7, 128.9, 128.4, 127.0, 126.8, 126.7, 126.5, 125.9, 125.6, 125.1, 124.8, 119.9, 119.6, 37.0, 20.8. HRMS (EI) calcd for $C_{30}H_{22}^+$ [M]$^+$, 382.1722, found: 382.1724.

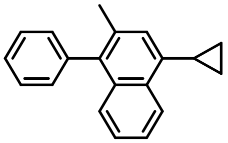

3bn

4-Cyclopropyl-2-methyl-1-phenylnaphthalene (**3bn**), 14.5 mg (yield: 28%, 0.2 mmol scale), white solid. ^{1}H NMR (400 MHz, $CDCl_3$) δ 8.42 (d, *J* = 8.4 Hz, 1H), 7.53 – 7.44 (m, 3H), 7.44 – 7.37 (m, 2H), 7.32 (dd, *J* = 15.7, 7.3 Hz, 1H), 7.28 – 7.15 (m, 3H), 2.47 – 2.29 (m, 1H), 2.19 (s, 3H), 1.22 – 0.97 (m, 2H), 0.82 (q, *J* = 4.7 Hz, 2H); ^{13}C NMR (100 MHz, $CDCl_3$) δ 140.0, 138.1, 136.6, 133.0, 132.6, 131.9, 130.3, 128.3, 126.9, 126.7, 126.7, 125.5, 124.6, 124.2, 20.8, 13.3, 6.3. HRMS (EI) calcd for $C_{20}H_{18}^+$ [M]$^+$, 258.1409, found: 258.1404.

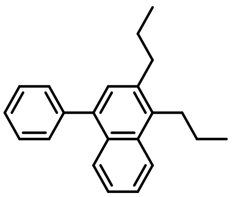

3bo

4-Phenyl-1,2-dipropylnaphthalene (**3bo**)[8], 26.3 mg (yield: 45%, 0.2 mmol scale), colorless oil. ^{1}H NMR (400 MHz, $CDCl_3$) δ 8.09 (d, *J* = 8.6 Hz, 1H), 7.87 (d, *J* = 7.9 Hz, 1H), 7.51 – 7.44 (m, 5H), 7.44 – 7.38 (m, 1H), 7.37 – 7.32 (m, 1H), 7.26 (s, 1H), 3.21 – 3.00 (m, 2H), 2.89 – 2.59 (m, 2H), 1.89 – 1.61 (m, 4H), 1.14 (t, *J* = 7.3 Hz, 3H), 1.03 (t, *J* = 7.3 Hz, 3H); ^{13}C NMR (100 MHz, $CDCl_3$) δ 141.1, 138.0, 137.0, 135.0, 132.5, 130.6, 130.2, 129.5, 128.1, 127.0, 126.6, 125.5, 124.5, 124.3, 35.8, 30.5, 24.8, 24.4, 14.8, 14.4.

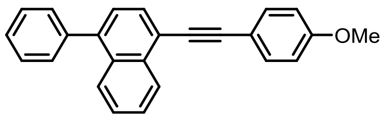

3bp

1-((4-Methoxyphenyl)ethynyl)-4-phenylnaphthalene (**3bp**), 21.4 mg (yield: 32%, 0.2 mmol scale), white solid. ^{1}H NMR (400 MHz, $CDCl_3$) δ 8.53 (d, *J* = 8.2 Hz, 1H), 7.93 (d, *J* = 8.4 Hz, 1H), 7.78 (d, *J* = 7.4 Hz, 1H), 7.64 – 7.55 (m, 3H), 7.54 – 7.35 (m, 7H), 7.00 – 6.82 (m, 2H), 3.84 (s, 3H); ^{13}C NMR (100 MHz, $CDCl_3$) δ 159.7, 140.6, 140.3, 133.5, 133.1, 131.4, 130.0, 129.6, 128.3, 127.4, 126.6, 126.5, 126.44, 126.416, 126.409, 120.7, 115.5, 114.1, 94.6, 86.4, 55.3. HRMS (EI) calcd for $C_{25}H_{18}O^+$ $[M]^+$, 334.1358, found: 334.1354.

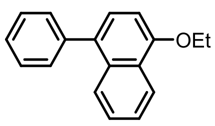

3bq

1-Ethoxy-4-phenylnaphthalene (**3bq**), 30.0 mg (yield: 60%, 0.2 mmol scale), white solid. ^{1}H NMR (400 MHz, $CDCl_3$) δ 8.38 (dd, *J* = 8.3, 0.8 Hz, 1H), 7.91 – 7.78 (m, 1H), 7.66 – 7.35 (m, 7H), 7.30 (d, *J* = 7.9 Hz, 1H), 6.83 (d, *J* = 7.9 Hz, 1H), 4.22 (q, *J* = 7.0 Hz, 2H), 1.55 (t, *J* = 7.0 Hz, 3H); ^{13}C NMR (100 MHz, $CDCl_3$) δ 154.2, 140.9, 132.4, 132.4, 130.2, 128.2, 126.9, 126.8, 126.4, 125.72, 125.66, 125.0, 122.3, 104.2, 63.7, 14.9. HRMS (EI) calcd for $C_{18}H_{16}O^+$ $[M]^+$, 248.1201, found: 248.1207.

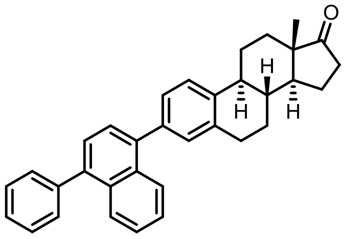

3br

(8R,9S,13S,14S)-13-methyl-3-(4-phenylnaphthalen-1-yl)-6,7,8,9,11,12,13,14,15,16-decahydro-17H-cyclopenta[a]phenanthren-17-one (**3br**), 58.4 mg (yield: 64%, 0.2 mmol scale), white solid. ^{1}H NMR (400 MHz, $CDCl_3$) δ 8.09 – 7.92 (m, 2H), 7.63 – 7.39 (m, 10H), 7.34 (d, *J* = 8.0 Hz, 1H), 7.29 (s, 1H), 3.16 – 2.78 (m, 2H), 2.64 – 2.48 (m, 2H), 2.42 (td, *J* = 11.0, 3.9 Hz, 1H), 2.25 – 1.93 (m, 4H), 1.82 – 1.65 (m, 2H), 1.62 – 1.46 (m, 3H), 1.42 (s, 1H), 0.96 (s, 3H); ^{13}C NMR (100 MHz, $CDCl_3$) δ 221.0, 140.8, 139.62, 139.59, 138.7, 138.2, 136.4, 131.87, 131.86, 130.7, 130.1, 128.2, 127.6, 127.2, 126.4, 126.3, 125.8, 125.7, 125.2, 50.5, 48.0, 44.4, 38.2, 35.9, 31.6, 29.5, 26.9, 26.6, 25.7, 21.6, 13.9. HRMS (ESI) calcd for $C_{34}H_{33}O^+$ $[M+H]^+$, 457.2526, found: 457.2525.

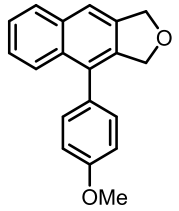

7a

4-(4-Methoxyphenyl)-1,3-dihydronaphtho[2,3-c]furan (**7a**)[9], 41.9 mg (yield: 76%, 0.2 mmol scale), white solid. ^{1}H NMR (400 MHz, $CDCl_3$) δ 7.85 (d, J = 8.1 Hz, 1H), 7.71 (d, J = 8.4 Hz, 1H), 7.67 (s, 1H), 7.51 – 7.42 (m, 1H), 7.41 – 7.34 (m, 1H), 7.32 – 7.25 (m, 2H), 7.07 – 6.99 (m, 2H), 5.29 (s, 2H), 5.03 (s, 2H), 3.89 (s, 3H); ^{13}C NMR (100 MHz, $CDCl_3$) δ 159.0, 137.6, 136.9, 133.6, 132.2, 132.0, 130.5, 130.2, 128.0, 125.7, 125.6, 125.5, 118.5, 114.0, 73.4, 72.9, 55.3.

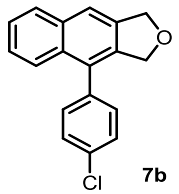

7b

4-(4-Chlorophenyl)-1,3-dihydronaphtho[2,3-c]furan (**7b**), 45.9 mg (yield: 82%, 0.2 mmol scale), white solid. ^{1}H NMR (400 MHz, $CDCl_3$) δ 7.87 (d, J = 8.1 Hz, 1H), 7.70 (s, 1H), 7.63 (d, J = 8.4 Hz, 1H), 7.52 – 7.43 (m, 3H), 7.42 – 7.35 (m, 1H), 7.31 – 7.26 (m, 2H), 5.28 (s, 2H), 5.00 (s, 2H); ^{13}C NMR (100 MHz, $CDCl_3$) δ 137.6, 136.9, 136.5, 133.7, 133.6, 131.6, 131.2, 130.8, 128.9, 128.1, 126.0, 125.7, 125.3, 119.1, 73.3, 72.7. HRMS (EI) calcd for $C_{18}H_{13}ClO^{+}$ $[M]^{+}$, 280.0655, found: 280.0664.

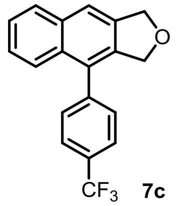

7c

4-(4-(trifluoromethyl)phenyl)-1,3-dihydronaphtho[2,3-c]furan (**7c**), 37.7 mg (yield: 60%, 0.2 mmol scale), white solid. ^{1}H NMR (400 MHz, $CDCl_3$) δ 7.89 (d, J = 8.1 Hz, 1H), 7.77 (d, J = 8.0 Hz, 2H), 7.73 (s, 1H), 7.58 (d, J = 8.4 Hz, 1H), 7.53 – 7.45 (m, 3H), 7.44 – 7.36 (m, 1H), 5.30 (s, 2H), 5.00 (s, 2H); ^{13}C NMR (100 MHz, $CDCl_3$) δ 142.0, 137.7, 136.9, 133.6, 131.4, 130.9, 129.90, 129.88 (q, J = 32.5 Hz), 128.2, 126.1, 125.9, 125.6 (q, J = 3.7 Hz), 125.2, 124.1 (q, J = 273.1), 119.5, 73.3, 72.6. HRMS (EI) calcd for $C_{19}H_{13}F_{3}O^{+}$ $[M]^{+}$, 314.0918, found: 314.0928.

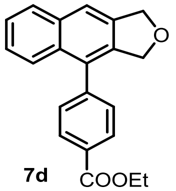

7d

Ethyl 4-(1,3-dihydronaphtho[2,3-c]furan-4-yl)benzoate (**7d**), 38.2 mg (yield: 60%, 0.2 mmol scale), white solid. ^{1}H NMR (400 MHz, $CDCl_3$) δ 8.19 (d, *J* = 8.3 Hz, 2H), 7.88 (d, *J* = 8.1 Hz, 1H), 7.72 (s, 1H), 7.61 (d, *J* = 8.4 Hz, 1H), 7.51 – 7.43 (m, 3H), 7.41 – 7.35 (m, 1H), 5.29 (s, 2H), 5.00 (s, 2H), 4.44 (q, *J* = 7.1 Hz, 2H), 1.44 (t, *J* = 7.1 Hz, 3H); ^{13}C NMR (100 MHz, $CDCl_3$) δ 166.4, 142.9, 137.7, 136.8, 133.6, 131.4, 131.3, 129.84, 129.81, 129.5, 128.2, 126.0, 125.8, 125.3, 119.3, 73.3, 72.7, 61.1, 14.4, 14.1. HRMS (EI) calcd for $C_{21}H_{18}O_3^+$ $[M]^+$, 318.1256, found: 318.1264.

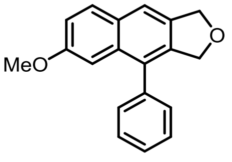

7e

6-Methoxy-4-phenyl-1,3-dihydronaphtho[2,3-c]furan (**7e**)[9], 42.5 mg (yield: 77%, 0.2 mmol scale), white solid. ^{1}H NMR (400 MHz, $CDCl_3$) δ 7.76 (d, *J* = 9.0 Hz, 1H), 7.61 (s, 1H), 7.53 – 7.46 (m, 2H), 7.45 – 7.39 (m, 1H), 7.39 – 7.33 (m, 2H), 7.14 (dd, *J* = 8.9, 2.5 Hz, 1H), 6.98 (d, *J* = 2.4 Hz, 1H), 5.26 (d, *J* = 0.7 Hz, 2H), 4.99 (s, 2H), 3.71 (s, 3H); ^{13}C NMR (100 MHz, $CDCl_3$) δ 157.6, 138.3, 137.3, 135.3, 132.8, 131.3, 129.4, 129.3, 129.1, 128.7, 127.6, 118.6, 117.9, 104.3, 73.3, 72.9, 55.1. HRMS (EI) calcd for $C_{19}H_{16}O_2^+$ $[M]^+$, 276.1150, found: 276.1160.

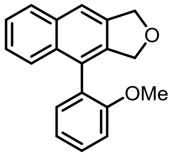

7f

4-(2-Methoxyphenyl)-1,3-dihydronaphtho[2,3-c]furan, (**7f**), 30.3 mg (yield: 55%, 0.2 mmol scale), white solid. ^{1}H NMR (400 MHz, $CDCl_3$) δ 8.10 (dd, *J* = 7.8, 1.2 Hz, 1H), 7.86 (d, *J* = 8.2 Hz, 1H), 7.69 (s, 1H), 7.64 (td, *J* = 7.5, 1.4 Hz, 1H), 7.54 (td, *J* = 7.7, 1.3 Hz, 1H), 7.46 – 7.40 (m, 1H), 7.31 (dd, *J* = 9.0, 2.4 Hz, 3H), 5.35 – 5.24 (m, 2H), 4.89 (q, *J* = 12.8 Hz, 2H), 3.45 (s, 3H); ^{13}C NMR (100 MHz, $CDCl_3$) δ 167.0, 139.1, 137.2, 136.1, 133.2, 132.3, 131.9, 131.8, 131.1, 130.6, 128.1, 128.0, 125.7, 125.4, 125.1, 118.7, 73.4, 72.7, 51.9. HRMS (EI) calcd for $C_{19}H_{16}O_2^+$ $[M]^+$, 276.1150, found: 276.1155.

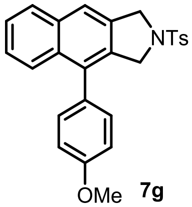

7g

4-(4-methoxyphenyl)-2-tosyl-2,3-dihydro-1H-benzo[f]isoindole (**7g**), 60 mg (yield: 70%, 0.2 mmol scale), white solid. ^{1}H NMR (400 MHz, $CDCl_3$) δ 7.79 (d, *J* = 8.1 Hz, 1H), 7.74 (d, *J* = 8.2 Hz, 2H), 7.60 (d, *J* = 4.5 Hz, 2H), 7.42 (t, *J* = 7.1 Hz, 1H), 7.33 (dd, *J* = 11.3, 4.1 Hz, 1H), 7.29 (d, *J* = 8.1 Hz, 2H), 7.17 (d, *J* = 8.6 Hz, 2H), 7.03 (d, *J* = 8.6 Hz, 2H), 4.78 (s, 2H), 4.47 (d, *J* = 13.6 Hz, 2H), 3.90 (s, 3H), 2.38 (s, 3H); ^{13}C NMR (100 MHz, $CDCl_3$) δ 159.1, 143.7, 134.1, 134.0, 133.5, 133.4, 133.3, 132.0, 130.4, 129.8, 129.5, 127.8, 127.5, 125.9, 125.8, 125.7, 120.4, 114.1, 55.3, 53.5, 53.1, 21.5. HRMS (ESI) calcd for : $C_{26}H_{24}NO_3S^+$ $[M+H]^+$, 430.1471, found: 430.1472.

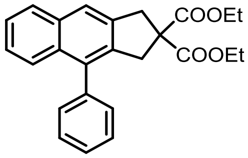

7h

Diethyl 4-phenyl-1,3-dihydro-2H-cyclopenta[b]naphthalene-2,2-dicarboxylate (**7h**)[10], 66.7 mg, (yield: 86%, 0.2 mmol scale), white solid. ^{1}H NMR (400 MHz, $CDCl_3$) δ 7.80 (d, *J* = 8.1 Hz, 1H), 7.67 (s, 1H), 7.57 (d, *J* = 8.4 Hz, 1H), 7.51 (dd, *J* = 10.1, 4.5 Hz, 2H), 7.46 – 7.40 (m, 1H), 7.40 – 7.34 (m, 3H), 7.32 – 7.26 (m, 2H), 4.17 (q, *J* = 7.1 Hz, 4H), 3.79 (d, *J* = 0.5 Hz, 2H), 3.47 (s, 2H), 1.22 (t, *J* = 7.1 Hz, 6H); ^{13}C NMR (100 MHz, $CDCl_3$) δ 171.4, 138.6, 138.4, 137.4, 135.0, 133.4, 131.8, 131.6, 129.8, 128.4, 127.7, 127.2, 125.7, 125.2, 125.1, 122.0, 61.7, 60.7, 40.3, 39.8, 14.0.

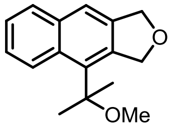

7i

4-(2-Methoxypropan-2-yl)-1,3-dihydronaphtho[2,3-c]furan (**7i**), 27.1 mg, (yield: 56%, 0.2 mmol scale), colorless oil. ^{1}H NMR (400 MHz, $CDCl_3$) δ 9.01 – 8.92 (m, 1H), 7.79 (dd, *J* = 6.1, 3.5 Hz, 1H), 7.61 (s, 1H), 7.50 – 7.40 (m, 2H), 5.35 (s, 2H), 5.15 (s, 2H), 3.08 (d, *J* = 8.3 Hz, 3H), 1.79 (s, 6H); ^{13}C NMR (100 MHz, $CDCl_3$) δ 138.1, 136.3, 134.9, 134.5, 131.4, 128.6, 127.1, 125.24, 125.21, 119.8, 80.3, 74.5, 71.9, 50.6, 28.8. HRMS (EI) calcd for $C_{16}H_{18}O_2^+$ $[M]^+$, 242.1307, found: 242.1317.

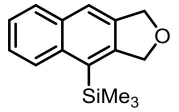

7j

(1,3-Dihydronaphtho[2,3-c]furan-4-yl)trimethylsilane (**7j**), 26.6 mg, (yield: 55%, 0.2 mmol scale), colorless oil. ^{1}H NMR (400 MHz, $CDCl_3$) δ 8.22 – 8.14 (m, 1H), 7.82 (dd, *J* = 6.5, 3.0 Hz, 1H), 7.68 (s, 1H), 7.51 – 7.40 (m, 2H), 5.28 (s, 2H), 5.18 (s, 2H), 0.50 (s, 9H); ^{13}C NMR (100 MHz, $CDCl_3$) δ 145.2, 137.0, 136.8, 132.9, 129.3, 129.0, 128.2, 125.2, 125.1, 121.2, 74.4, 72.2, 2.0. HRMS (EI) calcd for $C_{15}H_{18}OSi^+$ $[M]^+$, 242.1127, found: 242.1125.

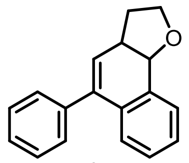

9

5-Phenyl-2,3,3a,9b-tetrahydronaphtho[1,2-b]furan (**9**), 17.4 mg, (yield: 35%, 0.2 mmol scale), colorless oil. General procedure: a schlenk tube equipped with a stir bar was loaded with 2.4 mg (3 mol%, 0.006 mmol) of Acr^+-Mes ClO_4^-, 9.5 mg (8 mol%, 0.016 mmol) of $Co(dmgH)_2py_2PF_6$, 0.2 mmol 1,1-diphenylethene and 1 mmol 2,3-dihydrofuran in 5 mL degassed DCE under N_2 atmosphere. The solution was then stirred at room temperature under the irradiation of 12W blue LED lamp for 24 h. ^{1}H NMR (400 MHz, $CDCl_3$) δ 7.47 (dd, *J* = 7.2, 1.4 Hz, 1H), 7.43 – 7.33 (m, 5H), 7.27 – 7.19 (m, 2H), 7.10 (d, *J* = 7.5 Hz, 1H), 5.73 (d, *J* = 3.1 Hz, 1H), 4.89 (d, *J* = 6.7 Hz, 1H), 3.93 (td, *J* = 8.2, 4.6 Hz, 1H), 3.80 (q, *J* = 7.7 Hz, 1H), 3.27 (ddd, *J* = 10.7, 7.6, 4.1 Hz, 1H), 2.47 (dq, *J* = 12.2, 8.1 Hz, 1H), 2.10 (ddd, *J* = 15.7, 7.7, 4.2 Hz, 1H); ^{13}C NMR (100 MHz, $CDCl_3$) δ 140.2, 138.3, 133.1, 132.9, 129.7, 129.1, 128.9, 128.5, 128.2, 127.6, 127.3, 126.0, 77.3, 66.4, 38.6, 33.5. HRMS (EI) calcd for $C_{18}H_{16}O^+$ $[M]^+$, 248.1201, found: 248.1205.

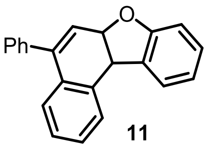

11

5-Phenyl-6a,11b-dihydronaphtho[2,1-b]benzofuran (**11**), 37.9 mg (yield: 64%, 0.2 mmol scale), white solid. General procedure: a schlenk tube equipped with a stir bar was loaded with 2.4 mg (3 mol%, 0.006 mmol) of Acr^+-Mes ClO_4^-, 9.5 mg (8 mol%, 0.016 mmol) of $Co(dmgH)_2py_2PF_6$, 0.26 mmol 1,1-diphenylethene and 0.2 mmol benzofuran in 5 mL degassed DCE under N_2 atmosphere. The solution was then stirred at room temperature under the irradiation of 12W blue LED lamp for 24 h. ^{1}H NMR (400 MHz, $CDCl_3$) δ 7.47 (d, *J* = 7.4 Hz, 1H), 7.40 – 7.26 (m, 6H), 7.15 (dd, *J* = 14.6, 7.4 Hz, 3H), 7.06 (d, *J* = 7.6 Hz, 1H), 6.92 – 6.79 (m, 2H), 5.89 (dd, *J* = 10.1, 3.2 Hz, 1H), 5.82 (d, *J* = 3.1 Hz, 1H), 4.76 (d, *J* = 10.1 Hz, 1H); ^{13}C NMR (100 MHz, $CDCl_3$) δ 158.1, 141.1, 139.4, 133.5,

131.5, 129.9, 128.9, 128.8, 128.5, 128.2, 128.0, 127.6, 127.1, 126.9, 124.6, 124.0, 120.6, 109.8, 81.0, 44.0. HRMS (EI) calcd for $C_{22}H_{16}O^+$ $[M]^+$, 296.1201, found: 296.1206.

Supplementary References

1 Liwosz, T. W. & Chemler, S. R. Copper-Catalyzed Oxidative Amination and Allylic Amination of Alkenes. *Chem.–Eur. J.* **19**, 12771-12777 (2013).

2 Dateer, R. B., Shaibu, B. S. & Liu, R.-S. Gold-Catalyzed Intermolecular [4+2] and [2+2+2] Cycloadditions of Ynamides with Alkenes. *Angew. Chem., Int. Ed.* **51**, 113-117 (2012).

3 Gerli, A. & Marzilli, L. G. Costa-type B12 models: synthesis, structural characterization, and electrochemistry of chloro derivatives. *Inorg. Chem.* **31**, 1152-1160 (1992).

4 Gudla, V. & Balamurugan, R. Synthesis of Arylnaphthalene Lignan Scaffold by Gold-Catalyzed Intramolecular Sequential Electrophilic Addition and Benzannulation. *J. Org. Chem.* **76**, 9919-9933 (2011).

5 Dikova, A., Cheval, N. P., Blanc, A., Weibel, J.-M. & Pale, P. Aryl and heteroaryl nosylates as stable and cheap partners for Suzuki–Miyaura cross-coupling reactions. *Tetrahedron* **72**, 1960-1968 (2016).

6 Chen, Z., Shou, W. & Wang, Y. One-Pot Synthesis of 1,4-Diarylnaphthalenes via a Wittig-Horner Reaction/[4+2] Cycloaddition/Dehydrogenation Sequence. *Synthesis* **2009**, 1075-1080 (2009).

7 Li, P.-F., Yi, C.-B., Ren, S.-J. & Qu, J. Ring-Opening/Expansion Rearrangement of Cycloprop[2,3]inden-1-ols Catalyzed by p-Toluenesulfonic Acid. *Adv. Synth. Catal.* **358**, 2088-2092 (2016).

8 Kabalka, G. W., Ju, Y. & Wu, Z. A New Titanium Tetrachloride Mediated Annulation of α-Aryl-Substituted Carbonyl Compounds with Alkynes: A Simple and Highly Efficient Method for the Regioselective Synthesis of Polysubstituted Naphthalene Derivatives. *J. Org. Chem.* **68**, 7915-7917 (2003).

9 Kudoh, T. *et al.* Intramolecular Anionic Diels−Alder Reactions of 1-Aryl-4-oxahepta-1,6-diyne Systems in DMSO. *J. Am. Chem. Soc.* **129**, 4939-4947 (2007).

10 Shibata, T., Fujiwara, R. & Takano, D. Thermal and Au(I)-Catalyzed Intramolecular [4+2] Cycloaddition of Aryl-Substituted 1,6-Diynes for the Synthesis of Biaryl Compounds. *Synlett* **2005**, 2062-2066 (2005).
